# G6PD testing and radical cure for *Plasmodium vivax* in Cambodia: A mixed methods implementation study

**DOI:** 10.1371/journal.pone.0275822

**Published:** 2022-10-20

**Authors:** Soy Ty Kheang, Rosemarie Ridley, Eng Ngeth, Por Ir, Pengby Ngor, Siv Sovannaroth, Dysoley Lek, Somaly Phon, Neeraj Kak, Shunmay Yeung

**Affiliations:** 1 The Center for Health and Social Development (HSD), Phnom Penh, Cambodia; 2 National Institute of Public Health (NIPH), Phnom Penh, Cambodia; 3 Clinical Research Department, Faculty of Infectious and Tropical Diseases, London School of Hygiene and Tropical Medicine (LSHTM), London, United Kingdom; 4 National Malaria Control Program, The National Center for Parasitology, Entomology and Malaria Control (CNM), Phnom Penh, Cambodia; Menzies School of Health Research, AUSTRALIA

## Abstract

**Introduction:**

Cambodia aims to eliminate malaria by 2025, however tackling *Plasmodium vivax* (*P*.*v*) presents multiple challenges. The prevalence of glucose-6-phosphate dehydrogenase (G6PD) deficiency has prevented the deployment of 8-aminoquinolones for “radical cure”, due to the risk of severe haemolysis. Patients with *P*. *vivax* have therefore continued to experience recurrent relapses leading to cumulative health and socioeconomic burden. The recent advent of point of care testing for G6PD deficiency has made radical cure a possibility, however at the time of the study lack of operational experience and guidance meant that they had not been introduced. This study therefore aimed to design, implement and evaluate a new care pathway for the radical cure of *P*.*vivax*.

**Methods:**

This implementation study took place in Pursat province, Western Cambodia. The interventions were co-developed with key stakeholders at the national, district, and local level, through a continuous process of consultations as well as formal meetings. Mixed methods were used to evaluate the feasibility of the intervention including its uptake (G6PD testing rate and the initiation of primaquine treatment according to G6PD status); adherence (self-reported); and acceptability, using quantitative analysis of primary and secondary data as well as focus group discussions and key informant interviews.

**Results:**

The co-development process resulted in the design of a new care pathway with supporting interventions, and a phased approach to their implementation. Patients diagnosed with *P*.*v* infection by Village Malaria Workers (VMWs) were referred to local health centres for point-of-care G6PD testing and initiation of radical cure treatment with 14-day or 8-week primaquine regimens depending on G6PD status. VMWs carried out follow-up in the community on days 3, 7 and 14. Supporting interventions included training, community sensitisation, and the development of a smartphone and tablet application to aid referral, follow-up and surveillance. The testing rate was low initially but increased rapidly over time, reflecting the deliberately cautious phased approach to implementation. In total 626 adults received G6PD testing, for a total of 675 episodes. Of these 555 occurred in patients with normal G6PD activity and nearly all (549/555, 98.8%) were initiated on PQ14. Of the 120 with deficient/intermediate G6PD activity 61 (50.8%) were initiated on PQ8W. Self-reported adherence was high (100% and 95.1% respectively). No severe adverse events were reported. The pathway was found to be highly acceptable by both staff and patients. The supporting interventions and gradual introduction were critical to success. Challenges included travel to remote areas and mobility of *P*.*v* patients.

**Conclusion:**

The new care pathway with supporting interventions was highly feasible with high levels of uptake, adherence and acceptability in this setting where high prevalence of G6PD deficiency is high and there is a well-established network of VMWs. Scaling up of the *P*.*v* radical cure programme is currently underway in Cambodia and a decline in reduction in the burden of malaria is being seen, bringing Cambodia a step closer to elimination.

## Introduction

### Plasmodium vivax in Cambodia

Among the countries in the Greater Mekong Subregion (GMS), Cambodia has the second highest malaria burden [1, 2]. Considerable progress has been made in reducing malaria in the past decade, with incidence declining from an estimated 7.4 cases/1000 population in 2006 to 1.9 cases/1000 population in 2019 [2, 3], and the country aims to achieve malaria elimination by 2025 [4]. However, more than 11 million individuals remain at risk of malaria and considerable barriers to elimination still exist [3, 5].

Firstly, malaria in this region is transmitted by female *Anopheles* mosquitoes residing in the forest with most transmission occurring during the rainy season (June-November) [5]. The key risk factor for malaria infection is therefore forest-going, which is primarily undertaken by adults, mainly males, involved in logging and construction in remote forested locations [4–6]. Secondly, both *Plasmodium vivax* (*P*.*v*) and *Plasmodium falciparum* (*P*.*f*) are endemic. Over the last two decades a huge amount of technical and financial resources have been invested in malaria control and elimination by the Cambodian government and international and local partners. Most interventions have focused on decreasing *P*.*f* burden and transmission, largely because the Cambodian-Thai border has been at the epicentre of multi-drug resistant *P*.*f* [7]. As a result, there has been a substantial decline in *P*.*f*. and by 2018, *P*.*f* comprised only 27% of malaria cases [3, 5]. Now, as Cambodia and its neighbours strive towards malaria elimination, increasing attention in being paid to the neglected and more difficult challenge of tackling *P*.*v*.

Unlike *P*.*f*, *P*.*v* forms hypnozoites, hepatic-stage parasites unaffected by artemisinin combination therapies (ACTs). Hypnozoites can lie dormant, and depending on the strain, reactivate repeatedly weeks, months or years after the initial infection. This may result in repeated relapses of febrile illness, leading to cumulative risk of complications including detrimental economic, educational and mental health outcomes [8, 9]. By disproportionately affecting primary wage-earners, relapses of *P*.*v* malaria result in a substantial economic impact perpetuating a cycle of poverty and poor health. The collective direct and indirect cost of a single *P*.*v* inoculation event over the years can be devastating.

Preventing relapses is critical to reducing malaria burden and to achieving elimination, especially in settings such as in Cambodia where the strains of *P*.*v* cause frequent relapses and where most symptomatic episodes are due to relapses rather than new infections [10, 11]. Preventing relapses could reduce incidence by more than half and would also reduce the human reservoir of parasites, and therefore transmission intensity [10].

### Radical cure

Eradication of hypnozoites to prevent relapses is known as “radical cure” [12]. Currently this requires treatment with a 14 day course of primaquine in patients with normal (≥80%) glucose-6-phosphate dehydrogenase (G6PD) activity [13, 14]. However, in individuals with G6PD deficiency (<30% activity) or intermediate deficiency (30–80% activity), an X-linked genetic disorder, the standard primaquine course can precipitate potentially life-threatening haemolysis requiring blood transfusions (which are difficult to access in low-resource settings and carry their own risks), and death if severe [15]. For those with G6PD deficiency, an adjusted regimen of primaquine can be given, once weekly over eight weeks which carries a lower risk of haemolysis, but effectiveness is more uncertain [13]. The risk of haemolysis has been a major barrier to providing radical cure in patients and achieving malaria elimination in Asia, by causing widespread fear and hesitancy to prescribe primaquine [12, 16]. Poor adherence to the long treatment courses can also be problematic, although directly observed therapy (DOT) and patient counselling can help [17, 18]. Crucially, without accessible point-of-care testing, G6PD status cannot be determined to guide treatment and reduce risk of complications.

### G6PD testing

The prevalence of G6PD deficiency (G6PDd) is estimated at 10–19% in males in Cambodia [19–23]. Therefore, although the national malaria treatment guidelines have recommended primaquine for the radical cure of *P*.*v*, they have also mandated that primaquine should only be administered with prior G6PD testing [12]. However, until recently testing for G6PD deficiency, either qualitatively (using fluorescent spot tests) or quantitatively (using spectrophotometric or cytochemical assays) has required laboratory facilities and equipment; refrigeration for reagent; and trained personnel, making them unsuitable for use in the remote rural settings where malaria patients are to be found [24, 25]. Therefore, in reality it has not been possible to offer individuals with *P*.*v* infection radical cure with primaquine resulting in an ongoing burden of repeated relapses [5, 12]. The recent commercialisation of both qualitative and quantitative point of care tests (POCTs) for G6PD has opened the possibility of changing this reality.

These POCTs do not require laboratory facilities, equipment or trained laboratory personnel and are quick and relatively easy to carry out. The qualitative lateral flow tests are performed by applying capillary blood obtained by finger prick to the sample well [26]. Results can be read after 10 minutes from the reading window, which stays colourless for G6PD deficient individuals but turns purple when normal G6PD activity is detected [26]. They can be used to detect reduced G6PD activity in males, but are considerably less sensitive and specific in females [27], because they are unable to accurately identify the intermediate levels of deficiency that heterozygous females may exhibit [24, 27, 28]. Determining G6PD status in females therefore requires quantitative tests [24, 29]. Quantitative POCTs are available in the form of “biosensors”, electronic handheld devices into which disposable testing strips or cartridges are inserted [30, 31]. Capillary blood is added, and results are provided as a quantitative read-out on the device screen in under five minutes. Some biosensors (e.g STANDARD^TM^ Biosensor) simultaneously measure haemoglobin [30], whilst others (e.g CareStart^TM^ Biosensor) require that a separate test of the haemoglobin level which is required in order to interpret results [31].

### Study rationale

At the time of this study, the Cambodian national malaria guidelines recommended radical cure with primaquine but only if preceded by a G6PD test. Lack of operational experience and guidance meant that these guidelines had not been implemented. The VIGTARC (*Plasmodium Vivax* G6PD Testing and Radical Cure) study aimed to address this gap by developing, implementing and evaluating a new care pathway for radical cure with primaquine, with preceding point-of-care G6PD testing and supporting interventions, tailored to the existing Cambodian healthcare system.

## Materials and methods

### Study design and setting

This was a mixed methods implementation study [32] comprising of the co-development and implementation of the intervention; and an evaluation using quantitative and qualitative methods. These are described in more detail below.

**Fig 1:**
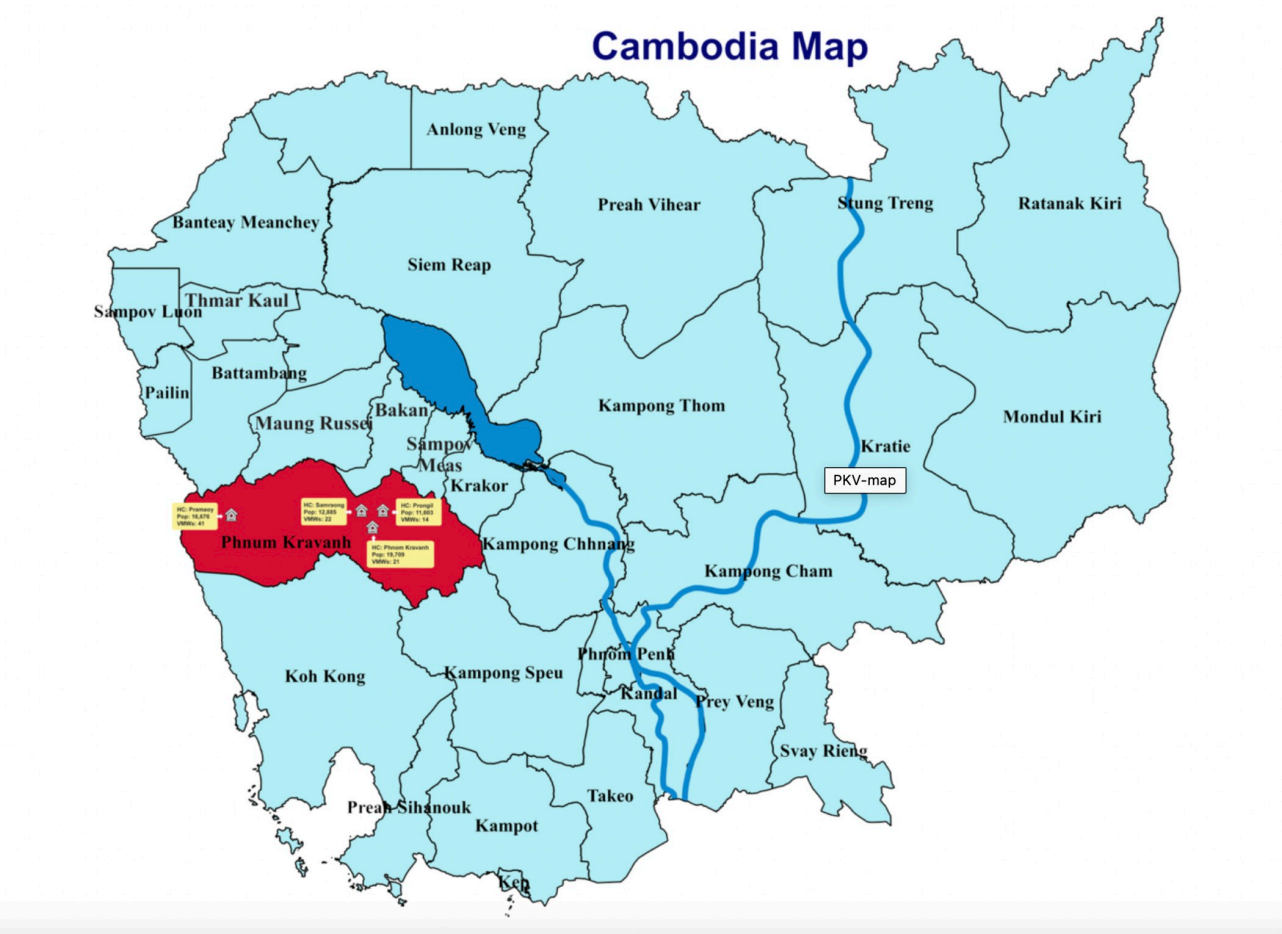
Map of Cambodia showing the four study HCs selected in Pursat province. This image is reproduced under a CC BY 4.0 license from https://hsdcambodia.org/malaria/.

The study took place in Pursat province in Western Cambodia, a hilly forested area on the border with Thailand and close to where artemisinin-resistant *P*.*f* malaria was first identified in 2008 [7, 33]. It has therefore been the focus of intense malaria control and elimination efforts. Pursat was selected because it had one of the highest incidence rates of *P*.*v* in the country [2]. Within Pursat province, the four health centres (HCs) with the highest incidence of *P*.*v* in the preceding year (2018) were purposively selected for this study (Fig 1). The distances between HCs and villages within their catchment areas are shown in S1 Table. In Pursat there has been an active ongoing *P*.*f* malaria elimination project lead by National Center for Malaria Control of Cambodia (CNM) and a non-governmental organisation. This includes additional support of the Village Malaria Workers (VMWs), key players in the national malaria control efforts [34]. Amongst other roles, the VMWs (two per village) are trained to diagnose malaria using RDTs (SD Malaria Antigen *P*.*f*/*P*.*v*) and provide treatment with 3-day artesunate-mefloquine for *P*.*f*, *P*.*v* and mixed (*P*.*f* and *P*.*v*) infections as per national guidelines.

### Intervention design and implementation

#### Process for the development of interventions

The intervention consisted of a core intervention (the care pathway) and supportive interventions. Central to the development of the interventions was the ethos that they had to be integrated into the existing health care infrastructure so that they could be rapidly and sustainably scaled up at a national level. The interventions were co-developed with key stakeholders including the CNM, other partners, local malaria control programme personnel and health facility staff through a continuous two-way exchange of information between the field team, research team, CNM and other partners via face to face, phone, text and email interactions, as well as more formal meetings at the national and local levels. This resulted in the design of the interventions, and the approach to implementing them, being closely informed by need and the experiences and findings from the research quickly being taken up into practice and incorporated into the national scale-up [35]. The key issues that were considered and the outcomes of the discussions are summarised in the results.

### Evaluation

Mixed methods were used to evaluate feasibility including uptake and adherence which were mainly assessed quantitively, and acceptability which was also assessed qualitatively.

Sources of quantitative data included: study data which was compiled by project staff from extracted VMW and HC records and entered into Excel databases; and data from Cambodia’s Malaria Information System (MIS). Analyses were conducted in Stata 16. Uptake was evaluated through the G6PD testing rate and the proportion of patients prescribed correct treatment according to G6PD status. The G6PD testing rate was calculated as the number of G6PD tests conducted during the ‘full launch’ period according to study. Patient adherence to prescribed medicines was measured as the proportion of patients completing the full primaquine course as self-reported by patients to the VMW divided by the number of *P*.*v* episodes recorded in MIS data over the same period in study areas.

Baseline characteristics of eligible participants who were tested for G6PD between July 2019 (study launch) until December 2020 (time of data extraction) was summarised using mean [SD], or median (IQR). Exact binomial (Clopper-Pearson) confidence intervals were calculated for estimates. Baseline prevalence of anaemia was calculated using World Health Organization (WHO) criteria [36].

Other outcomes included the prevalence of normal, intermediate, and deficient G6PD status amongst *P*.*v* patients, and risk of adverse events. Prevalence of different G6PD statuses were calculated using results of the quantitative tests for females, and qualitative tests for males as per the patient pathway determined by the study and described in results. Risk of adverse events was calculated as the proportion of patients who initiated primaquine that reported an adverse event requiring intervention or treatment cessation, which was monitored by VMWs at each follow-up appointment according to the patient pathway.

Acceptability of the new care pathway was also evaluated qualitatively through Focus Group Discussions (FGDs) and Key Informant Interviews (KIIs). These were conducted from August to November 2020. Four groups of participants from study areas (VMWs, HC staff, community leaders, and ex-patients who had received primaquine treatment) were purposively selected. Sampling strategy and interview format varied by participant group (Table 1).

**Table 1:** Sampling frame for the qualitative data collection.

Participant group	Sampling procedure	Data collection method	Total number of participants (number invited)
Village malaria workers (VMWs)	VMWs from selected villages*.	2 FGDs	18 (20)
Patients	Two patients who had received G6PD testing and primaquine, randomly selected from each of 10 villages*.	2 FGDs	17 (20)
HC staff	Three staff randomly selected from each study HC. Staff were excluded if they had never participated in VIGTARC activities.	12 KIIs	12 (12)
Community leaders	List of village chiefs and vice-chiefs/community leaders obtained for ten villages*; one individual randomly selected from each.	10 KIIs	10 (10)

HC = health centre. VMW = village malaria worker. FGD = focus group discussion. KII = Key informant interviews. G6PD = glucose-6-phosophate dehydrogenase. VIGTARC = *Plasmodium vivax* G6PD testing and Radical Cure Study.

*Villages randomly selected from study areas using probability proportional to size, depending on the number of patients recorded to have enrolled into the VIGTARC study from each village.

The qualitative research team comprised two external interviewers (both male, with undergraduate degrees in social science) and three research assistants (one male and two female; all experienced in community-based research projects) employed by the Center for Health and Social Development (HSD), all Cambodian. Topic guides were developed to guide discussion to subjects of interest determined *a priori* after discussion with stakeholders to consider which information would be most useful in informing future implementation (S2 Table). These included the acceptability of the new pathway, accessibility, experience using different G6PD tests and key challenges. Participants were invited to interview via phone. Interviews, which lasted 60–90 minutes, were conducted in local community halls (for VMWs, patients and community leaders) or private rooms in HCs (for HC staff). Travel costs were re-imbursed. Prior to interview, participants were informed of study aims verbally and in writing, and informed consent obtained. Consent and interview proceedings were conducted in Khmer, the local language. Interviews were audio-recorded with contemporaneous field notes, then translated afterwards into English by an HSD research assistant. Translations were verified by the Project Investigator (PhD MPH MD).

Thematic content analysis [37] was performed using NVivo 12. Data were coded by RR (MSc MBBS) using an abductive approach, to ensure that themes identified *a priori* for study objectives were evaluated, whilst allowing other themes to be added inductively as they became apparent through examination of data [37]. Themes selected *a priori* were chosen through review of previous literature which identified potential challenges and benefits, or through identifying interesting quantitative findings which required contextualisation. For example, a substantial reduction in malaria cases over time was observed during the study and was therefore explored. Initial coding lists and key recurring themes identified are listed in S3 Table. Key themes related to quantitative findings included in the main results have been described under relevant subheadings, with other key themes described in the supplement.

### Ethical approval

Ethical approval was granted by the LSHTM Ethics Committee (Reference 21990) and the National Ethics Committee for Health Research of Cambodia (Reference 044). The COREQ [38] and StaRI [39] checklists were adhered to (S4 and S5 Tables, respectively).

## Results

In this section we describe the final design of the care pathway and supporting interventions, preceded by a summary of the key considerations that arose during the process. We then present the results of the evaluation.

### The care pathway

In the process of co-developing the interventions and the approach to implementation, several key issues were considered. Many of these issues were decisions that needed to be incorporated into a “standard of care” such as the schedule for following up patients. As mentioned in the methods, these issues and options were presented and discussed between the research team, CNM, provincial and operational district malaria programme officers, and other stakeholders through in person meetings, email exchanges and phone calls. A summary of the issues, are presented in Table 2, along with what was finally implemented. Resources such as the training materials are included in S1 Appendix.

**Table 2:** Table summarising key considerations that arose during the process of designing the intervention.

Aspect of intervention	Question or issue	Considerations	What we did and why	Reflections
G6PD testing	Who should carry out the test: HC staff and/or VMW?	• Would VMWs be capable and willing to carry out? • Would health center staff have the time and be willing to carry out? • Who in the health centre? All staff or just specific individuals?	HC staff only (~ 2 staff/HC): *HC staff were willing*. *It was more possible to monitor use of G6PD tests*. *It was felt important that confidence and experience was gained at the HC level first*, *to ensure no unforeseen challenges in a less monitored environment*. *A clear system for referral from the community was developed which helped to support this approach*	
	Where should patients be tested? In the HCs and/or in the community?	• Related to issue above • Were storage conditions suitable in the community?	At the HCs only: *Testing was being carried out by HC staff only*. *A refrigerator was installed at each HC to ensure appropriate storage*. *There were concerns that temperatures would sometimes exceed 30°C in the villages which could compromise accuracy of G6PD tests*.	
	Who should be tested, and when?	• Only patients presenting with symptomatic *P*.*v* malaria or also asymptomatic “at-risk” patients? ◦ E.g if presenting at health centre with *P*.*f* malaria ◦ Mass screening in villages with high-incidence of *P*.*v*	Patients presenting with symptomatic *P*.*v* malaria only: *Symptomatic patients were the highest priority group who would benefit immediately from testing*. *There were insufficient resources*, *mainly in terms of staff time to organize and implement additional screening*.	
	Which test should be used? Quantitative and/or qualitative tests and which product?	• What is being recommended by experts? • Which tests is there the best evidence for? • What is available?	Initially Carestart^TM^ RDT for males, and Carestart^TM^ biosensors for females, then eventually SD biosensors for both males and females: *At the time of implementing study there was a lack of consensus expert opinion and delays in getting procured RDTs into the country in a timely manner*, *therefore the project started with the tests that were available in country*. *In the second year of the project*, *following CNM guidelines and the arrival of SD biosensors*, *there was a switch to the latter*.	
Primaquine treatment	Where should Primaquine be dispensed and by whom? At the HC (by HC staff) or in the community (by VMW)	• How is ASMQ (the first-line antimalarial treatment) supplied, dispensed and administered? • Are VMWs willing and capable of safely providing primaquine?	Primaquine course initiated at the HC by HC staff, but often with the help of the VMW: *Although the VMWs manage most patients with malaria in the community*, *including the administration of ASMQ*, *it was felt important that the HC staff were involved*, *at least initially*, *in the initiation of primaquine treatment*. *This was to ensure that it was provided with the appropriate explanations*. *As VMWs often accompanied the patients to the HC*, *they gradually took on this responsibility*.	It would have been good to monitor % of time patients who came to HC, came with VMW
Referral/ Linkage of care	By what means should the VMWs refer *P*.*v* patients to the HC for G6PD testing? (eg paper forms, SMS text, telephone, other)	• What was the existing means of communication between VMWs and the HC? • What were the CNMs plans with regards data on cases, testing, referrals etc?	Digitally using a tablet and smartphone-based app and in-person: *CNM already had in place the digital MIS*. *Health centres had been provided with tablets with the MIS app uploaded and VMWs were being supplied with smartphones*. *The study team therefore developed a P*.*v-specific module which was integrated into the app*. *HC staff and VMWs were trained in its use*. *Additionally*, *although not required to*, *the VMWs often physically accompanied the patients that they were referring to the HC*.	
	Any additional documentation?	• Mobile population, how to facilitate continuity of care? • Would a patient held card get lost? • What kind of information should be recorded?	A paper-based patient card (see S2 Appendix)	It would be useful to measure how often patients brought their patient card to them
Follow-up	Frequency and intensity	• What was ideal in terms of monitoring for side effects, and for monitoring adherence. • What was feasible for the VMWs to undertake?	On days 3, 7 and 14 the VMW visited patient, asked about side effects and self-reported adherence: *Three visits was the agreed compromise in terms of burden on the VMWs and need for close monitoring of adherence and side effects*. *For checking adherence*, *checking at the completion of the course (14 days) was felt to be important*, *plus a check within the first few days (day 3 or 4)*. *During the 8-week course*, *adherence was checkedin the first few days*, *then weekly*. *Side effects were reviewed daily on the 14-day course; on the 8-week course they were reviewed daily 3 days*, *then weekly until course completion*. *Checking around the expected nadir of haemoglobin (day 7–10) was felt to be important*.	
	What information to record and how?	• What is the manageable and useful?	Minimal data was collected on forms adapted from the one that VMWs were already using (see S3 Appendix).	
	Renumeration of VMW	• What is affordable and sustainable?	• Monthly meeting: $5 per-diem + $2–3 (rate) transportation (based on the distance) • Follow up visits: $2 per-diem + $ transportation (D4, D7, D14) • Prepaid phone card: $2 per month	
Females	To include at the beginning or not?	• Important to be equitable • Capacity of project staff, health centre staff and VMWs to implement multiple approaches at the same time safely • Availability of reliable G6PD testing • Proportion of patients who were females	Initially only males were referred for G6PD testing and radical cure then after 5 months, after additional training for HC staff and VMWs, females were also included: *There was some uncertainty about the reliability of the available qualitative sensor*. *Given the prevailing concerns with regards primaquine we felt it was important that the gaining of experience and confidence was paramount*. *It was therefore decided that for the first couple of months only adult males would be referred for G6PD testing and radical cure*, *whilst explaining to female patients that this would be made available to them shortly*. *Based on MIS data of the previous 18 months*, *the proportion of P*.*v cases that were female was 9*.*3%*.	It would have been good to formally document how the female patients and health care workers felt about this approach
Mobile/ smartphone application	Content and format	• What information should be recorded by the VMW, and by the HC	Basic patient details entered by VMW on each *P*.*v* patient, which get automatically uploaded so available to HC staff where patient is referred. HC staff enter results of G6PD tests and any treatment started. Follow-up dates ARE automatically generated with alerts being sent to VMWs.	
Training and community sensitisation	Content and format	• What information to include • Frequency, duration etc of training	See S1 Appendix for details.	

G6PD = glucose-6-phosphate dehydrogenase. HC = health centre. VMW = village malaria worker. P.f = *Plasmodium falciparum*. P.v RDT = rapid diagnostic test. ASMQ = artesunate-mefloquine. CNM = National Center for Malaria Control, Cambodia. MIS = Malaria information systems. SD = STANDARD^TM^.

**Fig 2:**
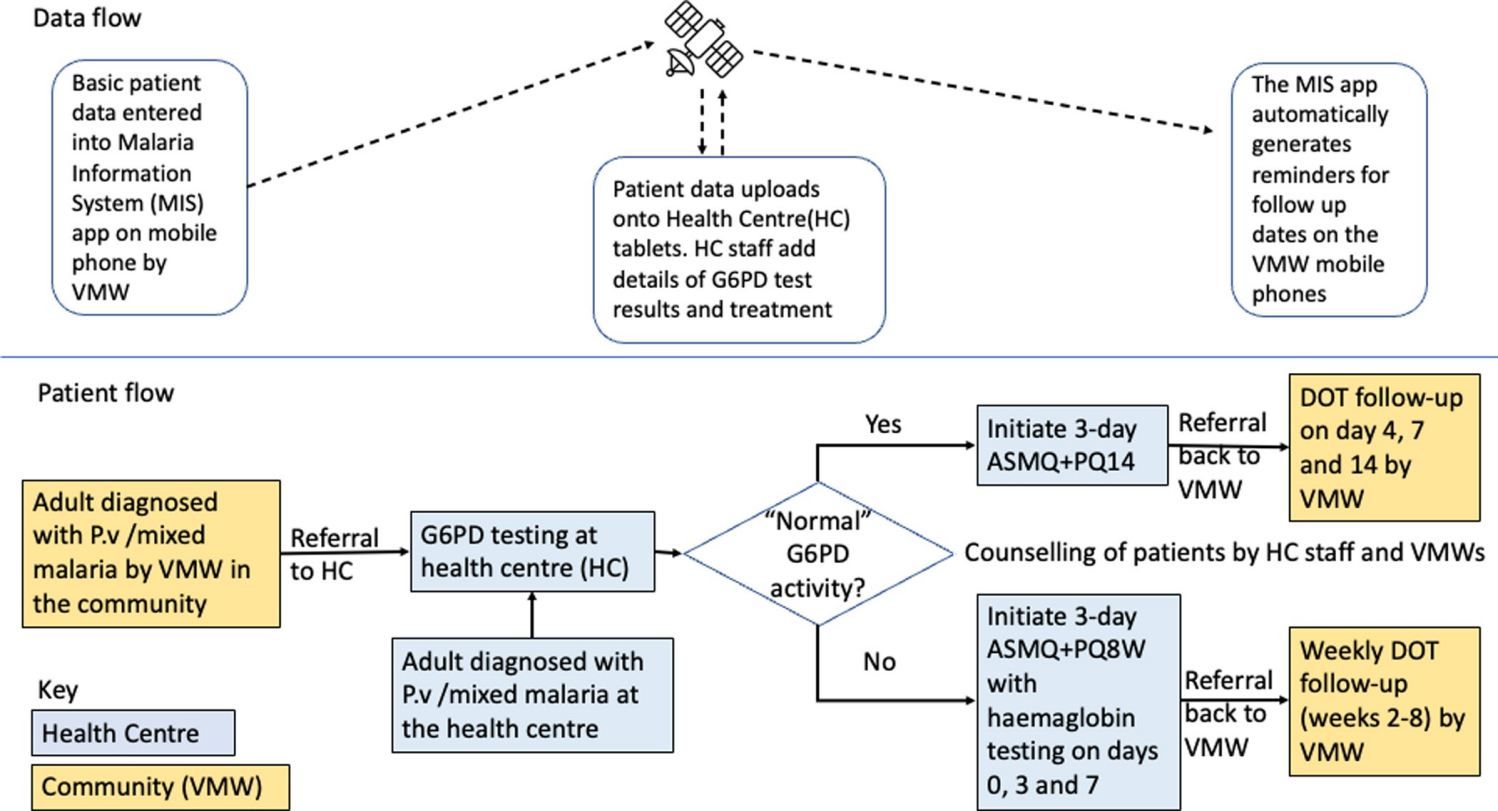
The new care pathway implemented in study areas. *P*.*v* = *Plasmodium vivax*. VMW = village malaria worker. G6PD = glucose-6-phosphate dehydrogenase. ASMQ = artesunate-mefloquine. PQ14 = 14-day primaquine course. PQ8W = 8-week primaquine course. DOT = directly observed therapy. MIS = Malaria Information Systems (of the National Malaria Center of Cambodia). *Diagnosed by SD Malaria Antigen *Plasmodium falciparum*/*Plasmodium vivax* rapid diagnostic test. ^†^By quantitative test (CareStart^TM^ Biosensor or STANDARD^TM^ Biosensor) for females, and by qualitative (CareStart^TM^ RDT) +/- quantitative test for males; patients were excluded from testing/treatment if pregnant/breastfeeding, aged <15 years, haemoglobin <9 g/dL, body weight <20kg. ^‡^By STANDARD^TM^ Biosensor or by HemoCue® test.

Fig 2 illustrates the final design of the new care pathway that was implemented. In brief, adults diagnosed with *P*.*v* or mixed malaria could enter the care pathway through either referral by VMWs (who screened for eligible individuals that presented to them in the community), or through HC screening (if patients presented to HCs directly). Exclusion criteria were: pregnancy/breastfeeding, children aged <15 years (because parental consent required), declining to consent, severe clinical illness on presentation, and weight <20kg. Those excluded were treated as per national guidelines with ASMQ alone [14].

HCs were initially supplied with qualitative lateral flow tests (CareStart^TM^ RDT) and the quantitative CareStart^TM^ Biosensor tests and test strips, and then later with the STANDARD^TM^ Biosensor tests and test strips. HC staff were trained to carry out quantitative testing with a biosensor for female patients and qualitative tests on male patients; how to record, interpret (based on thresholds recommended in national treatment guidelines [14]) and use the result to guide primaquine therapy (see S1 Appendix for training materials and events delivered to HC staff and/or VMWs). A limited number of biosensors and strips were also made available for use on male patients so that the HC staff preference for different types of tests could be explored. They were asked to record the result, but not to use it to guide treatment.

Patients with normal G6PD results were counselled according to a script (S1 Appendix) and initiated on standard primaquine treatment of 14 daily doses (PQ14), then referred to the VMW for follow-up on days 3, 7 and 14. On follow-up days, the patient was directly observed taking primaquine and asked about side effects including dark urine, looking pale, feeling more tired or breathless (see S3 Appendix for follow-up form). On the other days, the VMWs were instructed to call the patients to check self-reported adherence and ask about side effects. VMWs were given $2 per follow-up visit ($6 for three visits) plus cost of transport, plus $2 per month for pre-paid telephone card.

The 8-week primaquine course (PQ8W), administered under DOT weekly for 8 weeks, was later introduced for those with G6PDd. Primaquine dosing was based on weight (S6 Table). Patients found to have a haemoglobin level <9.0 g/dL (measured using STANDARD^TM^ Biosensor or by HemoCue^®^ test) were not given primaquine. Adverse events were monitored at weekly DOT sessions.

#### Gradual (phased) implementation

**Fig 3:**
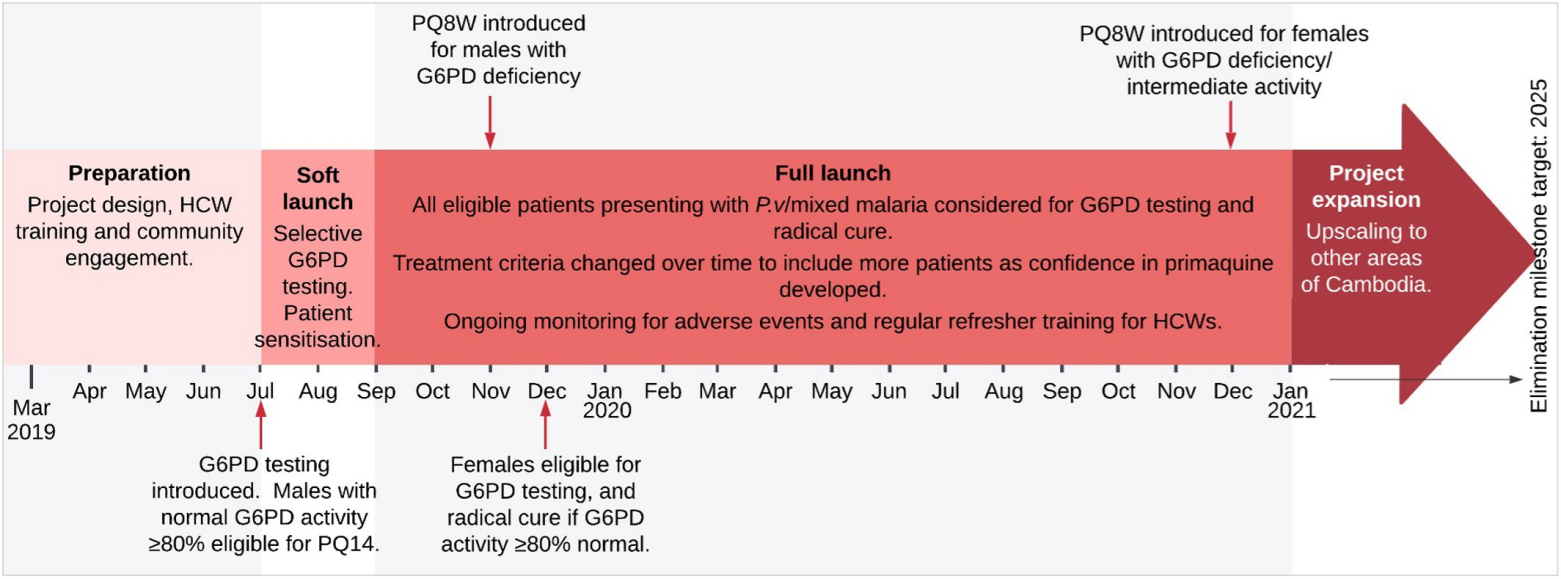
Timeline of implementation of the G6PD testing and radical cure programme. G6PD = glucose-6-phosphate dehydrogenase. *P*.*v* = *Plasmodium vivax*. PQ14 = 14-day primaquine course. PQ8W = 8-week primaquine course. HCW = healthcare worker (including village malaria workers and health centre staff).

Due to a history of hesitancy using primaquine in Cambodia, it was decided that a cautious phased approach should be used in implementing the care pathway to allow the VMWs and HC staff to familiarise themselves with the processes, to monitor carefully for adverse events, and so everyone could gain confidence in the safety of the approach (Fig 3). A ‘soft launch’ took place between July to August 2019 which allowed community sensitisation and development of healthcare worker (HCW) confidence in using G6PD tests and primaquine. At this stage only adult male patients were referred for G6PD testing and only those who tested not G6PD deficient were given 14-day primaquine treatment and initially followed up daily. ‘Full launch’ began on 1^st^ September 2019, with a reduction in the number of follow up visits, and the gradual inclusion of women and G6PD deficient males.

#### Supporting interventions

Supporting interventions included HCW training, community sensitisation, mobile app development and an emergency preparedness plan.

#### Training and community sensitisation

Training of HCWs, and the sensitisation of patients and the community were essential components of the intervention. The training covered *P*.*v* and relapses; radical cure with primaquine; G6PD deficiency and the risk of haemolysis; and G6PD testing. HC staff received training on performing G6PD tests, interpreting results, counselling patients, and choosing appropriate treatment. Refresher and additional training sessions were conducted tailored to the different audiences with various levels of detail and additional modules e.g on carrying out and interpreting different types of G6PD tests and on how to use the mobile/tablet application (described below). Training materials included some developed by CNM and partners and some developed by the project team. Details of the schedule for training, workshops and community sensitisation events are included in S1 Appendix.

#### Digital technology and patient card for communication and real-time surveillance

To facilitate referral, follow up, communication and real-time malaria surveillance, a *P*.*v* module was developed by the study team and integrated into the existing CNM Malaria Information Systems (MIS) tablet/smartphone application. Tablets were provided to each HC, and smartphones to each VMW, accompanied by initial training and ongoing support. The app was used to record patient details, test results and medicines prescribed. It also automatically provided VMWs with the follow-up schedule and sent reminder notifications on follow-up days (see S4 Appendix for screenshots).

Additionally, all participants were provided with a paper patient card in which their G6PD status was recorded along with any antimalarial treatment prescribed (S2 Appendix). They were encouraged to keep the card carefully and present it to HCWs. Patients who re-presented with *P*.*v* having previously completed a course of primaquine were eligible for referral back to HCs for re-treatment with primaquine, based on their recorded G6PD status.

#### Emergency preparedness plan

Finally, an emergency preparedness plan, including referral pathway, was established in case of complications requiring medical intervention. This included a clear line of referral from the VMW to the referral hospital, training, and preparation of facilities and equipment at the provincial referral hospital to receive patients for blood transfusion if needed.

### Evaluation

#### Description of participants

666 patients received G6PD testing between July 2019 and December 2020. Of these, 626 were eligible for inclusion and are included in the analysis. Of the excluded patients, most (39/40) were children <15 years, and one was a pregnant woman.

Of the 626 participants, two thirds (409/626) 65.3% of the participants were referred by VMWs; the remaining 34.7% (217/626) presented directly to HCs. All were diagnosed with *P*.*v* monoinfection. Most participants (576/626, 92.0%) were male. The median age at enrolment was 26 years (IQR 19–34). 620/620 (100.0%) patients self-reported previous *P*.*v*/mixed infection(s), and 15.2% (94/620) reported suffering ≥10 previous *P*.*v*/mixed malaria episodes in their lifetime (Table 3). Of the 626 participants, 49 had repeat presentations giving a total of 675 episodes.

**Table 3:** Characteristics of the study population (N = 626).

Variable	Category	n (%), mean [SD], or median (IQR)
Sex		
	Male	576 (92.0%)
	Female	50 (8.0%)
Health centre		
	Kravanh	200 (32.0%)
	Promoy	172 (27.5%)
	Samrong	167 (26.7%)
	Prongil	87 (13.9%)
Age (years)		26 (19.0–34.0)
Weight (kg)		56.5 [9.3]
Type of infection*		
	*P*.*v* monoinfection	625 (100.0%)
	*Missing data*	*1*
Mobile vs non-mobile		
	Non-mobile	522 (83.4%)
	Mobile	104 (16.6%)
History of previous malaria infection reported by patients		
	No	0 (0.0%)
	Yes	620 (100.0%)
	Previous *P*.*v* monoinfection	620 (100.0%)
	Previous *P*.*f* monoinfection	7 (1.1%)
	Previous mixed (*P*.*f* and *P*.*v*)	2 (0.3%)
	*Missing data*	*6*
Number of previous *P*.*v*/mixed malaria infections reported by patients		
	0	0 (0.0%)
	1–3	329 (53.1%)
	4–6	143 (23.1%)
	7–9	54 (8.7%)
	≥10	94 (15.2%)
	*Missing data*	*6*
Location of *P*.*v* diagnosis		
	Community (by VMW)	409 (65.3%)
	Health centre	217 (34.7%)

*P*.*v* = *Plasmodium vivax*. *P*.*f* = *Plasmodium falciparum*. VMW = village malaria worker.

*Species of malaria diagnosed at presentation. No participants were found to have mixed malaria infection (both *P*.*v* and *P*.*f*).

Baseline haemoglobin was measured in 453/626 (72.4%) participants using Hemocue® or STANDARD^TM^ test results. Prevalence of anaemia was 35.8% (95%CI 31.5–40.3%) according to WHO classification [36]. Coverage and results of different haemoglobin tests are shown in S7 Table.

#### Uptake and adherence

**Fig 4:**
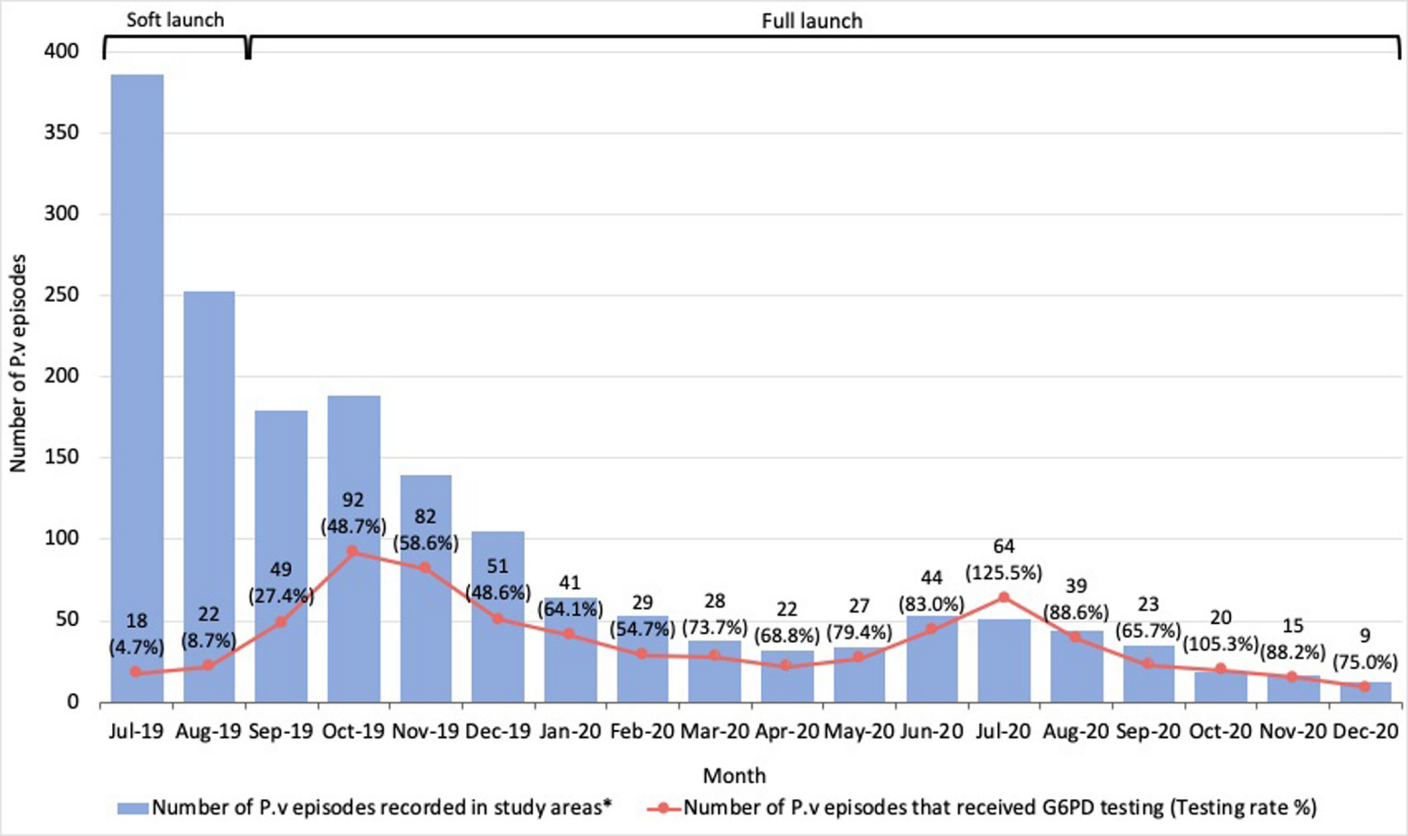
Number of *P*.*v* episodes and G6PD testing rate in study areas, by month. P.v = *Plasmodium vivax*. G6PD = glucose-6-phosphate dehydrogenase. *According to national MIS data. ^†^G6PD testing using qualitative test (CareStart^TM^ rapid diagnostic test) for males and quantitative test (CareStart^TM^ Biosensor or STANDARD^TM^ Biosensor) for females.

Of the 626 eligible patients that received G6PD testing, 40 were tested during the ‘soft launch’; the remaining 586 patients were tested during the ‘full launch’ period (September 2019 to December 2020) and had a total of 635 *P*.*v* episodes. From the MIS data, 1065 *P*.*v*/mixed malaria episodes were recorded in the study areas during this period. The overall testing rate was therefore estimated at 59.6% (635 *P*.*v* episodes that received G6PD testing divided by 1065 *P*.*v* episodes recorded in study areas), and gradually increased over time (Fig 4). Of note, this increase in testing rate is explained not only by the increase in the number of cases tested for G6PD but also a dramatic decline in the number of *P*.*v*, shortly after the soft launch of the project (S5 Appendix).

The relatively low initial testing rate reflects the deliberately slow introduction, with VMWs only referring patients who after careful counselling were ready and willing to participate. The main reasons that patients did not want to participate were because of the difficulty in committing to the 14 days of follow up and because of the economic imperative for them to work, including travelling back to the forest. Of note, the testing rate exceeded 100% in July 2020 and October 2020. When data were explored in greater detail, this was partly attributable to patients coming from villages outside of the HC catchment areas (S1 Fig). There was also qualitative evidence that some individuals waited several days before presenting to HCs after referral by VMWs, which could mean they were tested in the month following their initial diagnosis (S6 Appendix). For patients who were referred by VMWs, all completed referral and received G6PD testing at HCs.

Although no formal comparison was conducted between HCs, G6PD testing rates varied across sites and were 47.3% at Kravanh, 61.2% at Prongil, 66.5% at Promoy and 77.5% at Samrong. The number of *P*.*v* episodes and G6PD testing rate over time is shown for each HC in S2 Fig.

**Fig 5:**
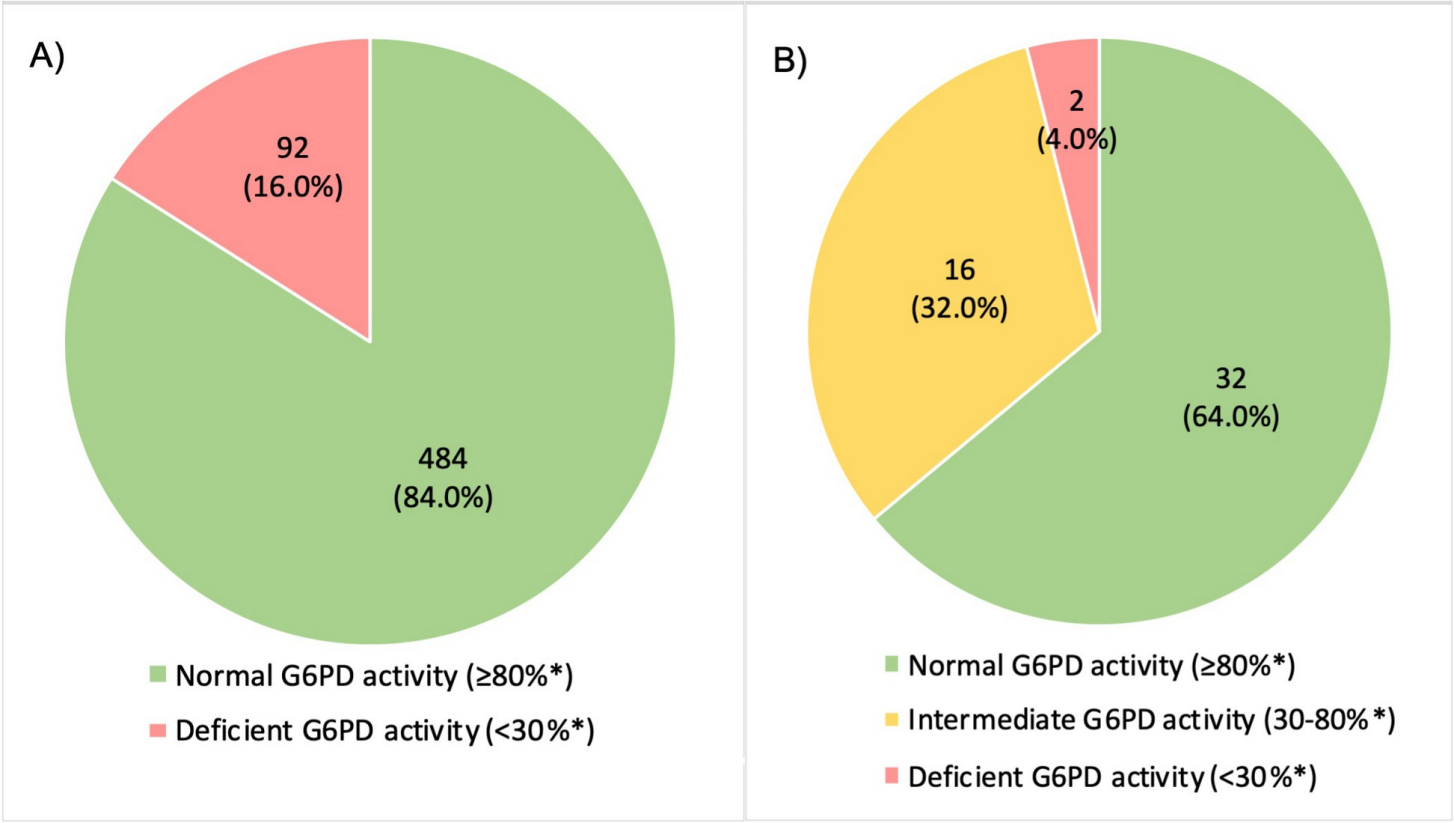
G6PD status of study participants. Number (%) of patients with normal, intermediate and deficient levels of G6PD activity in A) males tested for G6PD status (N = 576) and B) females tested for G6PD status (N = 50). G6PD = glucose-6-phosphate dehydrogenase. *% residual G6PD activity, measured using CareStart^TM^ rapid diagnostic test for male patients and either CareStart^TM^ Biosensor or STANDARD^TM^ Biosensor test for female patients.

All 50 (100%) female patients received quantitative testing and all 576 (100%) male patients received qualitative RDTs as per study guidelines. 16.0% (92/576) of males tested G6PD deficient by RDT; prevalence of intermediate G6PD status and deficient G6PD status in females were 32.0% (16/50) and 4.0% (2/50) respectively (Fig 5). 197/576 males (34.2%) also received quantitative testing. The study aims did not include a formal comparison of the qualitative and quantitative G6PD test results, but qualitatively there appeared to good agreement (S3 Fig).

**Fig 6:**
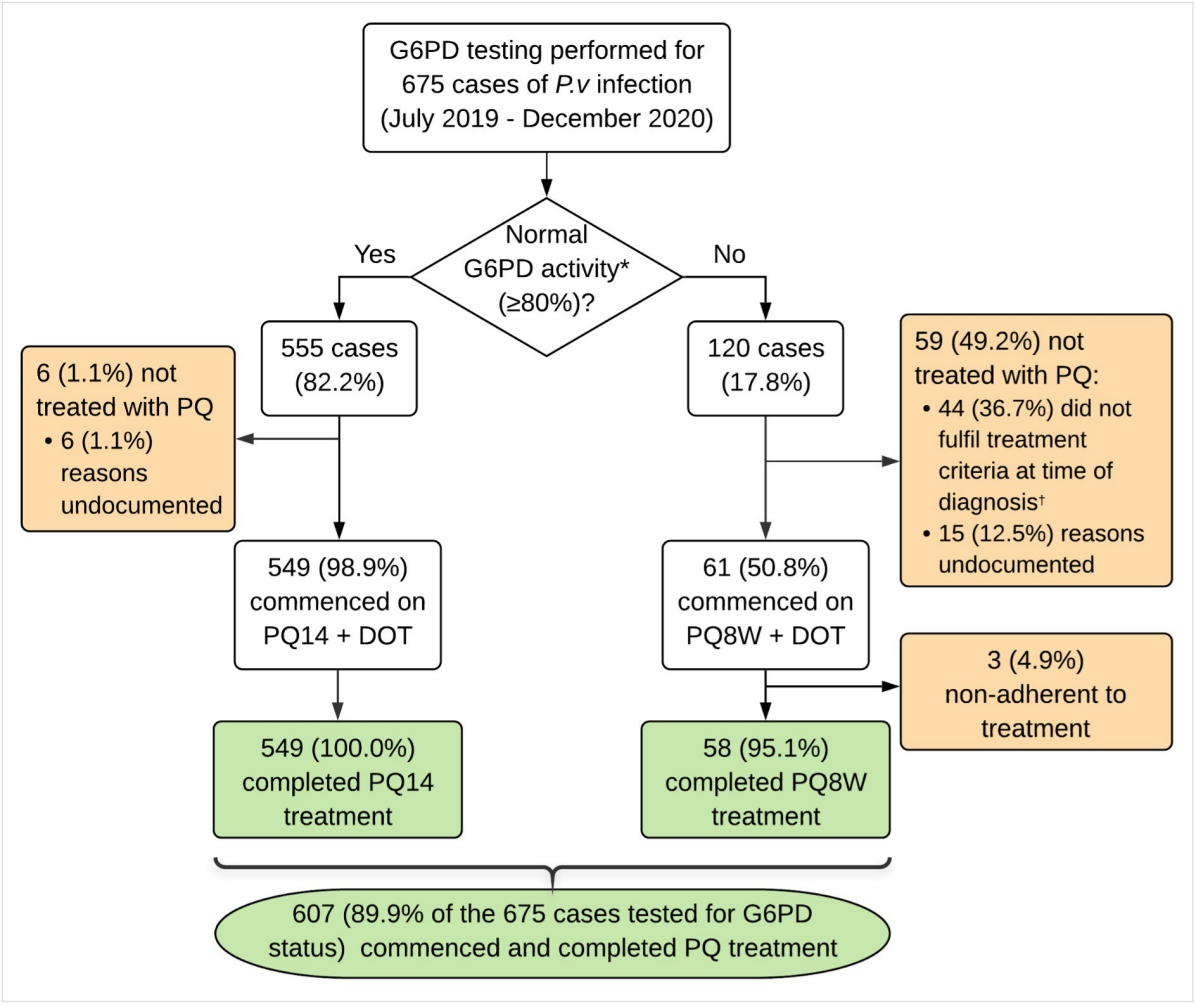
Progression of participants through the care pathway. G6PD = glucose-6-phosphate dehydrogenase. PQ = primaquine. PQ14 = 14-day primaquine. PQ8W = 8-week primaquine. DOT = directly observed therapy. *G6PD normal activity (≥80%) determined by CareStartT^M^ RDT for males, and CareStart^TM^ Biosensor (≥6.0 U/g Hb) or STANDARD^TM^ Biosensor (≥6.0 U/g Hb) for females. ^†^Treatment criteria changed over time, as shown in Fig 3.

Of 675 episodes, 610 were appropriately initiated on primaquine treatment (Fig 6). This included 549/555 (98.8%) with normal G6PD activity initiated on PQ14, of which 549 (100.0%) completed the course, and 61/120 (50.8%) with deficient/intermediate G6PD activity initiated on PQ8W, of which 58 (95.1%) completed the course. In total, 607/675 (89.9%) were initiated on primaquine and were fully adhered to treatment.

No severe adverse events requiring medical intervention and/or primaquine cessation occurred following primaquine treatment. Between days 0 and 7 of PQ8W treatment, mean haemoglobin decreased by 1.8 g/dL (13.4%). S7 Appendix further describes haemoglobin trends.

### Acceptability

Overall, a recurring theme in all participant groups was the request for the project to be extended. Below the perceptions about different aspects of the intervention are explored.

#### G6PD testing

Experience of using G6PD tests varied amongst HC staff interviewed. Overall, quantitative tests were preferred over qualitative. Qualitative tests took longer, could present ambiguous colour results that were difficult to interpret and were inadequate for females; these disadvantages outweighed the fact that qualitative tests were often reported as technically easier to perform.

*Actually*, *the RDT test is not clear like biosensor test*. *Sometimes*, *when we tested using it*, *the result showed the colour unclearly*. *So*, *sometimes it is hard for us to diagnose whether the result is deficient or normal*. *But biosensor test is good because it shows G6PD clearly and haemoglobin clearly as well*.

*HC05*, *HC Staff*

Both VMWs and HC staff expressed concerns about the capacity for VMWs to correctly perform and interpret G6PD tests due to high variation in skills and knowledge, and lack of reliable cold storage in villages.

#### Primaquine uptake, adherence and perceived efficacy

Patients were highly motivated to receive testing and radical cure, because of the economic impacts of recurrent relapses. Treated patients frequently encouraged peers to participate and counselling was described as important for encouraging uptake and achieving high intervention coverage.

*After that [the treated patient] told another patient to come here for radical cure like him/her as well*. *This is benefit*. *Before radical cure*, *he/she was relapsed frequently once a month and after radical cure with primaquine*, *he/she forced their relatives and co-worker who was sick for treatment as well*. *This is lot of cases*.

*HC11*, *HC Staff*

Many patients needed to travel into forests to earn income, however treatment required staying in villages for follow-up. The primary reason for patients declining primaquine was felt to be the prioritisation of short-term economic benefits of returning to work quickly. For a small minority, travel difficulties and/or travel costs were barriers, however traveling to HCs for testing was considered easy for most. VMWs frequently accompanied them to facilitate referral.

*There was a patient who said that he committed not to be treated with it [primaquine]*. *He was in debt… Therefore*, *he needed to go to forest every day [to work]… They don’t have times for this medicine drinking*.

*V[Unknown]*, *VMW*

Although some patients described being sceptical of efficacy initially, all perceived primaquine was highly effective in preventing *P*.*v* relapse at interview. Perceived efficacy was also described through its indirect impact on other areas of life, especially in reducing the negative effects on socioeconomic status and health.

*At the beginning*, *I didn’t believe this medicine*. *I heard from VMW who said that nowadays [we have] the medicine for* P.v *radical cure for 14 days treatment*, *but we hadn’t believed yet*. *Now we treat with it and [it] can heal [for] many months now*, *from 2019 until now*. *I never reoccurred or relapsed with it*. *Therefore*, *I just want to say “[I] Like this medicine which can make me heal from malaria and I have time to work to support my life”*.

*P01*, *Ex-patient*

Interview participants universally felt that malaria incidence had steeply declined in study areas from 2019 to 2020. Although the purpose of this study was not to demonstrate effectiveness, incidence in study areas was explored; findings support interviewee perceptions of substantial reduction in incidence (S5 Appendix). Most participants felt the cause was multifactorial. Four key contributors were identified: restrictions on forest-going, radical cure availability, establishment of VMWs and utilisation of bite-avoidance (prevention) methods. HCWs felt that historically, a large proportion of cases comprised a small number of individuals who relapsed repeatedly and often. They extrapolated that by treating a relatively small number of patients to prevent relapses, a dramatic reduction in incidence had resulted. Perceived efficacy in reducing incidence motivated HCWs to encourage patient uptake.

*In previous times*, *the rate of the malaria increased because of the same cases which relapsed one time a month which accounted for 12 times or 7 or 8 times per year; so*, *these cases were only one patient; therefore*, *the cases had increased*. *Nowadays*, *when we treat patient(s) with radical cure*, *we don’t see the same person*. *It means that we see only the new case(s) or new infection(s) because we don’t see the former* P.v *patients who already were treated… Therefore*, *we have seen that the cases have dropped down a lot; and the programme is good for them*.

*HC08*, *HC Staff*

The care pathway was widely perceived as very beneficial and manageable for HCWs and patients alike. DOT follow-up was highlighted as a major strength that improved adherence and safety and strengthened VMW-patient relationships. Patients were happy to be followed up at home as they could continue home-based work. The PQ14 DOT follow-up schedule (day 3, day 7, day 14) was described as adequate and sufficient by 10/12 (83.3%) HC staff and 18/18 (100.0%) VMWs interviewed; 2/12 (16.7%) HC staff felt more sessions would benefit. However, many VMWs conducted extra follow-up in addition to the 3-day schedule, either via phone or in person with the aim of ensuring adherence and/or safety. Therefore, patients actually received varying amounts of follow-up.

Attending follow-up was sometimes challenging for VMWs due to travel costs and poor road conditions, especially during the rainy season to remote communities. For particularly hard-to-reach patients, HCWs suggested follow-up could be improved by establishing VMWs specifically for these areas, enabling follow-up via phone (which some VMWs had performed if travel was impractical), and/or reimbursing travel costs. Aside from occasional phone follow-up and extra DOT sessions delivered by VMWs, high fidelity to the proposed care pathway was described by HCWs and patients.

No interviewed patients reported non-adherence; they felt the long treatment duration was acceptable even if they couldn’t work during it, because of substantial long-term opportunity costs caused by recurrent relapses. Mobile patients were felt to be at increased risk of non-adherence, as they travelled frequently.

*The weakness is [the] mobile patient*. *It is hard to convince them to use radical cure 14 days*, *8 weeks*. *Because of the duration*, *they could not adhere*. *Therefore*, *this is the weakness of the project*.

*HC12*, *HC Staff*

#### Safety

Primaquine was widely perceived as safe. None of those interviewed had witnessed or experienced serious adverse events, however risk of haemolysis was acknowledged. HCWs expressed that correct usage of G6PD tests, patient counselling, timely drug administration and regular follow-up mitigated primaquine-associated risks. Mild adverse events were felt to occur initially and self-resolve between days 3 and 5 of PQ14 treatment. Many considered these early symptoms to be artesunate-mefloquine-related (rather than primaquine-related), from previous experience with artesunate-mefloquine. Patients were mostly asymptomatic whilst taking primaquine exclusively.

#### Supportive interventions

Generally, the use of mobile phones and tablets to document and track referrals was very well-received, although some VMWs expressed difficulty in using the smartphone.

*V06*: *The difficulty is smart phone*.

*V04*: *I don’t know to use it*.

*V06*: *Not very good at using*. *Forgetting*.

*V09*: *Be careful*, *they stop buying for using…*

*VMW discussion*

Full results of the thematic analysis including additional key themes (HCW training; Data management; Supply and procurement; Tafenoquine) are described in S6 Appendix.

## Discussion

These findings provide good evidence for feasibility of integrating a new care pathway for providing point-of-care G6PD testing and radical cure with primaquine in Cambodia. Along with other initiatives [40], our study is providing operational experience to help with the ongoing scaling up of G6PD testing (by SD biosensor) and primaquine in the country which is based on the care pathway implemented in this study including a similar follow-up schedule on days 3, 7 and 14 of treatment. Of note, the CNM decided to only implement the SD biosensor rather than rapid tests. The main reported reasons being the preference for clarity of the numeric result rather than *t*he different colours on the test band, which has been a challenge for staff to read or interpret; and because the biosensor provides a haemoglobin result at the same time with G6PD result.

The interventions in this study were deliberately implemented gradually in a phased approach, to allow HCWs to gain familiarity with guidelines, proficiency in performing and interpreting G6PD tests and for both them and the community to gain confidence in the use of primaquine. Although this meant that the initial testing rate was low and some groups were not initially included, gaining experience and confidence in this manner was important prior to including more complex subgroups such as females, who require quantitative testing. This was important in the Cambodian context, where there is a background of primaquine hesitancy and early errors could have shattered public and HCW confidence. Using this gradual approach, no patients were prescribed an incorrect treatment course and excellent safety outcomes were observed. The high levels of acceptability achieved are reflected in the qualitative interviews where participants recurrently expressed that their support for the programme stemmed from witnessing others who had received safe and effective treatment, even when they had been initially unsure.

G6PD testing was only undertaken at HCs in this study, and not in the community. Although a few participants did feel that the need travel to HCs did limit access, shifting G6PD testing and the initiation of primaquine treatment fully into community does not seem viable within the current infrastructure; VMWs and HC staff both felt VMWs were unequipped to perform G6PD testing effectively.

A very high proportion (89.9%) of cases who were tested for G6PD status were prescribed and completed a course of primaquine. Follow up schedule on days 3, 7, and 14 was both feasible and widely accepted. Only three patients failed to complete a treatment course, all of whom had been initiated on PQ8W treatment. Other studies have also documented challenges of adherence to long treatment courses [41, 42], and in this setting was most likely due to the opportunity costs associated with staying in villages for follow-up.

No haemolysis or severe adverse events were observed, consistent with patient and HCW perceptions of treatment safety. We provide less evidence for safety in PQ8W compared to PQ14 treatment, due to smaller sample size and loss to follow-up of three patients, possibly resulting in attrition bias.

### Limitations

This was a pragmatic implementation research study, aimed to provide operational guidance to inform the imminent implementation and scale up of G6PD testing within the Cambodian health care settings. Implementation of the programme was largely undertaken by existing HC staff and VMWs, with the small project team playing a limited role. As VMWs were responsible for both ensuring adherence and monitoring it, there is a possibility that adherence has been overestimated. Whilst a controlled trial may have produced a higher level of evidence, there was neither the time nor the resources to implement such a study. For similar reasons, we were not able to collect some process outcomes such as formal assessments of training and community sensitisation, and patient card retention. It would be useful to collect such data in future studies.

The qualitative interviews were designed to allow open and honest engagement, through delivery in private settings by trained interviewers that were not part of local communities. However, the presence of interviewers who had been recruited and trained by HSD potentially influenced responses. Participants may have a personal stake in seeing the project continue and therefore over-emphasize benefits. It is worth noting however that these vested interests of patients and community leaders are contingent on interventions being perceived as effective. VMWs (who work on a voluntary basis) also would gain very little, as the programme increases their workload.

Study areas were purposively selected for their high burden of *P*.*v* cases and it is possible that outcomes may differ in low-burden settings where awareness of *P*.*v* and perception of importance of radical cure may differ. In this model of care, community volunteers played a central role in the care pathway. This was possible because a well-established network of VMWs was already place and actively involved in malaria case management and surveillance. In countries with different health systems, the care pathway piloted in this study may not be appropriate.

The sample size of participants treated with PQ8W and the number of females participants was relatively small; the latter reflecting the general epidemiology and their later inclusion in the study. Further studies will be important to ensure that the care pathway and supporting interventions are appropriately tailored to this group.

## Conclusion and recommendations

The ongoing experience and findings from the study fed directly into the CNM’s scaling up of radical cure for *P*.*v* across Cambodia, which began several months after the start of this study. This included the care pathway, the schedule for follow up, the implementation of the MIS app and other supportive interventions including training of HC staff and VMWs and community sensitisation. The phased implementation of the interventions was important in building confidence and ensuring acceptability in this context where historically there has been much concern about primaquine use. As experience and confidence is gained at the level of the national malaria control programme, the implementation may be accelerated. However, we would still advocate ensuring that the speed of implementation matches the local readiness of HC staff, VMWs and the community. In terms of testing, both HC staff and VMWs felt that testing should be carried out by HC staff. The latter also expressed an overall preference for the quantitative biosensor tests. This finding coincided with a decision by WHO and the CNM to only implement the SD biosensor and not qualitative RDT results. In terms of treatment, we had limited data on women and on the 8-weekly primaquine course. Gathering more information on these cohorts would be very helpful. Of note, whilst single-dose tafenoquine (TQ) may be an option in the future, for safety reasons it is likely that a follow up of patients would still be required, quantitative G6PD testing is mandatory and TQ is not recommended with blood-stage ACT and thus not a current option for Cambodia. Finally, we recommend establishing specific strategies for particularly hard-to-reach patients, for example phone follow-up by VMWs or assigning specific VMWs to remote areas.

### Concluding statement

Interviewees’ descriptions of huge socioeconomic repercussions of recurrent relapses reiterates the importance of establishing radical cure, as does the high prevalence of anaemia in this vivax-affected population. Over 11 million individuals in Cambodia continue to be at risk of *P*.*v* and the associated health and socioeconomic impacts [3]. There is good evidence to support feasibility and acceptability of the proposed care pathway in this rural, low-resource setting with high population prevalence of G6PD deficiency and anaemia, where good uptake was achieved despite historical primaquine hesitancy [12, 16]. Rapid scaling up of the *P*.*v* radical cure programme in Cambodia is currently underway and is resulting in a reduction in *P*.*v* burden and a step towards elimination [35].

## Supporting information

S1 Table Distance from study health centers (HCs) to villages within their catchment area. (DOCX)

S2 Table Topics of interest included in topic guides for discussion during interviews, identified *a priori*. (DOCX)

S3 Table Initial coding lists, key themes and discarded themes identified through thematic content analysis. (DOCX)

S4 Table The Consolidated Criteria for Reporting Qualitative Research (COREQ): 32-item checklist [38]. (DOCX)

S5 Table Standards for Reporting Implementation Studies: The StaRI checklist [39]. (DOCX)

S6 Table Recommended weight-based primaquine doses. (DOCX)

S7 Table Coverage and results of different baseline haemoglobin tests in study population. (DOCX)

S1 Fig The total number of G6PD tests performed, by month, and the proportion that were performed on patients from a villages within the catchment area of the HC, outside the catchment area, or whose villages are unknown (undocumented). (DOCX)

S2 Fig Number of P.v episodes and G6PD testing rate by month, for A) Kravanh HC, B) Promoy HC, C) Samrong HC, and D) Prongil HC.
(DOCX)

S3 Fig Comparison of qualitative and quantitative G6PD test results. (DOCX)

S1 Appendix Healthcare worker training and education materials, and methods of delivery. (DOCX)

S2 Appendix G6PD patient card. (DOCX)

S3 Appendix Paper forms used by HC staff and VMWs to collect and store patient information. (DOCX)

S4 Appendix Screenshots of the *P*. *vivax* module, integrated into the National Center for Malaria Control’s MIS (Malaria Information Systems) mobile app. (DOCX)

S5 Appendix Trends in malaria incidence in study areas. (DOCX)

S6 Appendix Findings from thematic content analysis of qualitative interviews. (DOCX)

S7 Appendix Analysis of haemoglobin trends in the 8-week primaquine (PQ8W) course [43]. (DOCX)
