# Supplementary material for: G6PD testing and radical cure for Plasmodium vivax in Cambodia: A mixed methods implementation study
Source: PLoS One. 2022 Oct 20;17(10):e0275822. doi: 10.1371/journal.pone.0275822 (PMC9584508; doi:10.1371/journal.pone.0275822)
Supplement: S1 Table — (DOCX) [file pone.0275822.s001.docx]

**S1 Table:** Distance from study health centers (HCs) to villages within their catchment area.

| **Name of HC** | **Name of village within catchment area of HC** | **Distance from village to HC** | **Distance from OD to HC** | **Distance from PHD to HC** |
| --- | --- | --- | --- | --- |
| Phnom Kravanh HC |  |  | 0 km | 32 km |
|  | Krouchmar | 2 km |  |  |
|  | Leach | 2 km |  |  |
|  | Pichban | 2 km |  |  |
|  | Paen | 3 km |  |  |
|  | Kultortoeung | 3 km |  |  |
|  | Bohbouy | 4 km |  |  |
|  | Santrae | 4 km |  |  |
|  | Preysmach | 4 km |  |  |
|  | Preykhlong | 4 km |  |  |
|  | Sbovrik | 5 km |  |  |
|  | Tanok | 5 km |  |  |
|  | Baktrakuon | 6 km |  |  |
|  | Srae Popeay | 13 km |  |  |
|  | Ksetborey | 15 km |  |  |
|  | Mulrokat | 15 km |  |  |
|  | Vealvong | 15 km |  |  |
|  | Mulchah (Annex) | 35 km |  |  |
|  | Meat (Annex) | 18 km |  |  |
|  | Teuk Lo Eok (Annex) | 15 km |  |  |
|  | Ream Ray (Annex) | 25 km |  |  |
|  | Dong Pul (Annex) | 22 km |  |  |
|  | Cheu (Annex) | 33 km |  |  |
|  | Khsaing (Annex) | 35 km |  |  |
|  | Reang Khvav (Annex) | 46 km |  |  |
|  | Patchnang (Annex) | 39 km |  |  |
|  | O Dar (Annex) | 42 km |  |  |
|  | Ampil (Annex) | 52 km |  |  |
| Prongil HC |  |  | 8 km | 24 km |
|  | Kampaeng | 2 km |  |  |
|  | O Srav | 2 km |  |  |
|  | Svaypak | 3 km |  |  |
|  | Prongil | 4 km |  |  |
|  | Samraongyea | 6 km |  |  |
|  | O Baktra | 6 km |  |  |
|  | Say | 25 km |  |  |
| Samrong HC |  |  | 8 km | 42 km |
|  | Praekmouy | 3 km |  |  |
|  | Praekpir | 5 km |  |  |
|  | Praekbei | 4 km |  |  |
|  | O Heng | 4 km |  |  |
|  | Samraongmouy | 6 km |  |  |
|  | Samraongpir | 9 km |  |  |
|  | O Preal | 7 km |  |  |
|  | Tadaes | 15 km |  |  |
|  | Veal | 25 km |  |  |
|  | Angkrong | 30 km |  |  |
|  | Rorveang | 35 km |  |  |
|  | Anlongpaen | 13 km |  |  |
|  | Vealnintrea (annex) | 22 km |  |  |
| Promoy HC |  |  | 108 km | 142 km |
|  | Pramaoy | 4 km |  |  |
|  | Tumpor | 13 km |  |  |
|  | Phcheukchrum | 12 km |  |  |
|  | Chheutealchrum | 13 km |  |  |
|  | Taporngpong | 62 km |  |  |

HC = Health center. PHD = Provincial Health Department. OD = Operational District (The OD for all four health centres included in the study was Phnom Kravanh OD).
