# Supplementary material for: G6PD testing and radical cure for Plasmodium vivax in Cambodia: A mixed methods implementation study
Source: PLoS One. 2022 Oct 20;17(10):e0275822. doi: 10.1371/journal.pone.0275822 (PMC9584508; doi:10.1371/journal.pone.0275822)
Supplement: S2 Table — (DOCX) [file pone.0275822.s002.docx]

**S2 Table:** Topics of interest included in topic guides for discussion during interviews, identified *a priori*. “X” indicates where topic was included for the participant group.

| **Topic of interest** | **HC staff** | **VMWs** | **Patients** | **Community leaders** |
| --- | --- | --- | --- | --- |
| Understanding of what malaria and *P.v* is |  |  | X |  |
| *P.v* epidemiology and burden in the local area | X | X | X | X |
| Perceived malaria incidence trends over past year, and contributing factors | X | X |  | X |
| Description and experience of care received for *P.v* |  |  | X |  |
| Description and experience of delivering care, including fidelity to the designed care pathway | X | X |  |  |
| Awareness of the VIGTARC radical cure programme |  |  | X | X |
| Strengths and challenges of radical cure programme | X | X | X | X |
| Understanding and perceptions of VMW role in the community | X | X |  | X |
| Perceived reasons for patients accepting/declining radical cure | X | X | X | X |
| Experience using G6PD tests | X |  |  |  |
| Capacity for VMWs to perform G6PD testing | X | X |  |  |
| Training of HCWs | X | X |  |  |
| Data management | X | X |  |  |
| Primaquine supply | X | X |  |  |
| Safety of primaquine and adverse events | X | X | X |  |
| Efficacy of primaquine | X | X | X |  |
| Effect of radical cure programme on HCW workload | X | X |  |  |
| Perceptions of DOT follow-up schedule | X | X | X | X |
| Tafenoquine treatment as an option to replace primaquine | X | X | X |  |
| Community sensitisation and education about the radical cure project | X | X | X | X |
| Private sector contribution to malaria management in the local area | X | X |  | X |

*P.v* = *Plasmodium vivax.* VIGTARC = *Plasmodium vivax* G6PD testing and radical cure study. G6PD = glucose-6-phosphate dehydrogenase. VMW = village malaria worker. HCW = healthcare worker. DOT = directly observed therapy.
