# Supplementary material for: G6PD testing and radical cure for Plasmodium vivax in Cambodia: A mixed methods implementation study
Source: PLoS One. 2022 Oct 20;17(10):e0275822. doi: 10.1371/journal.pone.0275822 (PMC9584508; doi:10.1371/journal.pone.0275822)
Supplement: S3 Table — (DOCX) [file pone.0275822.s003.docx]

**S3 Table:** Initial coding lists, key themes and discarded themes identified through thematic content analysis.

| Key themes | Codes* | Description |
| --- | --- | --- |
| 1. G6PD testing^†^ | Experience using tests | Experience of interviewee with using different G6PD tests, both performing and interpreting results. |
|  | Experience being tested | Experience of interviewee being tested for G6PD status. |
|  | Benefits of testing | Perceived benefits of testing *P.v* patients for G6PD status. |
|  | Challenges of testing | Perceived challenges to interviewee arising from G6PD testing. |
|  | Preference between different test types | Interviewee preference for one test type over another, if any, and rationale. |
|  | VMW capacity to perform G6PD tests | Perceived capacity for VMWs to perform G6PD testing for patients in the future, instead of/in addition to HC staff. |
| 2. Primaquine treatment^†^ | Aggregation of sub-theme codes | Aggregation of sub-theme descriptions. |
| 2a. Uptake^†^ | Patient motivations for uptake | Factors that motivated patients to agree to, or decline, G6PD testing +/- primaquine treatment. |
|  | HCW counselling to promote uptake | How HCWs promoted uptake through counselling, how it was received by patients, and influence in patients’ decision-making. |
|  | Referral process | Strengths and challenges of the referral process to HCs, to initiate G6PD testing and radical cure. |
|  | Accessibility of HCs | Ease of access to HCs for patients referred from VMWs, and any challenges such as travel barriers and cost. |
|  | Recommending peers for radical cure | Willingness of patients that were treated with radical cure to recommend to peers (including family, friends or colleagues) that they should also receive G6PD testing +/- radical cure. |
| 2b. Delivery in new care pathway^†^ | User experience | Experience of patients treated in the new care pathway. |
|  | Provider experience | Experience of service providers (HCWs) in delivering G6PD testing and radical cure in the new care pathway, including impact to workload. |
|  | Fidelity to the care pathway | Whether participants were managed in keeping with the proposed care pathway and associated guidelines, or whether participants were actually managed in alternative ways. |
|  | Strengths of the new care pathway | Strengths and highlights of the new care pathway, including G6PD testing and primaquine treatment. |
|  | Weaknesses of the new care pathway | Weaknesses and challenges of the new care pathway. |
|  | Project extension/expansion | Requests from interviewees for project extension in study areas or expansion to other areas of Cambodia. |
| 2c. Adherence^†^ | Directly observed therapy follow-up | Experience with directly observed therapy follow-up including what happened during follow-up appointments and any perceived challenges or strengths. |
|  | Reasons for non-adherence | Perceived reasons why some participants did not, or found it difficult to, adhere to the treatment course. |
| 2d. Efficacy^†^ | Mechanism of treatment | How primaquine is perceived to work in the body. |
|  | Efficacy in reducing relapses | Personal or second-hand experience of primaquine in preventing *P.v* relapses, or failing to prevent *P.v* relapses. |
|  | Effect on local malaria incidence | Perceived effect of the introduction of radical cure on local malaria incidence/burden, if any. |
|  | Effect on health and physical abilities | Perceived effect of primaquine treatment on health of patient(s), or their ability to perform physical activities, if any. |
|  | Socioeconomic impact | Perceived effect, if any, of primaquine treatment on patients’ social and economic activities, including ability to carry out productive work. |
| 2e. Safety^†^ | Safety | Perceived safety, or lack of safety, of giving primaquine to *P.v* patients in the PQ14 or PQ8W courses in the new care pathway. |
|  | Adverse events | Knowledge or experience (first- or second-hand) of adverse events (or suspected adverse events) perceived to have been precipitated by primaquine treatment. |
|  | Haemolysis | Acknowledgement/awareness of haemolysis as a possible adverse event, or description of haemolysis experienced by self or others due to primaquine. |
| 3. HCW training^‡^ | Amount of training/guidelines received | Amount of training/guidelines on G6PD testing and management of *P.v* patients with radical cure that was received by the interviewee, if interviewee was a HCW. |
|  | Adequacy of training/preparation | Whether or not training received was perceived as adequate in preparing the interviewee for managing patients in the new care pathway, if interview was HCW. |
|  | HCW understanding of radical cure for *P.v* | Interviewee understanding of benefits, risks or mechanism of action of radical cure for *P.v*, if interview was a HCW. |
| 5. Data management^‡^ | Smartphone/tablet app | HCW experience using the *P.v* module of the MIS smartphone/tablet app, including benefits and challenges. |
|  | G6PD patient card | HCW and patient experience and understanding of the G6PD patient card, including benefits and challenges. |
| 6. Primaquine supply and procurement^‡^ | Adequacy of primaquine supply | Perceived adequacy of primaquine supply, including issues with procurement and stock breaks. |
| 5. Tafenoquine^‡^ | Acceptability of tafenoquine | Acceptability of tafenoquine (single-dose radical cure), described to interviewees, as a possibility for introduction in the future. |
| **Discarded Themes** | | |
| Malaria history | Species | Which malaria species the interviewee (if a previous or current patient) had previously been diagnosed with during their lifetime. |
|  | Number of *P.v* relapses | Number of *P.v* episodes the interviewee (if a previous or current patient) had experienced during their lifetime. |
|  | Burden of *P.v* | Perceived burden of *P.v* infection on the interviewee’s life, including socioeconomic and health impacts. |
| Haemoglobin testing | Experience using tests | Experience of HCWs testing patients for haemoglobin. |
| Public awareness of *P.v*/radical cure | Existing awareness of malaria | Level of awareness of the local population regarding malaria including difference between species, risk factor, pathophysiology, and treatment options. |
|  | Healthcare-seeking behaviour | Healthcare-seeking behaviour of individuals with malaria/suspected malaria. |
|  | Promotional events | History and experience of events such as community meetings in promoting awareness of *P.v* and the radical cure programme. |
| Private sector contribution | Management of malaria in the private sector | Knowledge or experience of malaria cases that were treated in the private sector. |
|  | Referrals from the private sector | Knowledge or experience of malaria cases that were referred from the private sector to public HCs for treatment in the new care pathway. |
| Artesunate-mefloquine | Efficacy | Perceived efficacy of artesunate-mefloquine for acute malaria. |
|  | Adverse events | Adverse events perceived to be precipitated by artesunate-mefloquine. |

G6PD = glucose-6-phosphate dehydrogenase. *P.v* = *Plasmodium vivax*. PQ14 = 14-day primaquine course. PQ8W = 8-week primaquine course. HCW = healthcare worker. HC = health centre, VMW = village malaria worker.

*Code concepts and themes were identified both deductively and inductively through line-by-line coding by researcher RR (MBBS MSc DTM&H; clinical research fellow; female) and reviewed by SY (PhD MBBS FRCPCH MRCP DTM&H; Professor of infectious disease and global health; female).

^†^Key themes reported in main text results.

^‡^Key themes reported in supplementary materials (S4 Appendix).
