# Supplementary material for: G6PD testing and radical cure for Plasmodium vivax in Cambodia: A mixed methods implementation study
Source: PLoS One. 2022 Oct 20;17(10):e0275822. doi: 10.1371/journal.pone.0275822 (PMC9584508; doi:10.1371/journal.pone.0275822)
Supplement: S4 Table — (DOCX) [file pone.0275822.s004.docx]

**S4 Table:** The Consolidated Criteria for Reporting Qualitative Research (COREQ): 32-item checklist [39].

| **Item number** | **Item** | **Guide questions/description** | **Reported on page number** |
| --- | --- | --- | --- |
| **Domain 1: Research team and reflexivity** | | | |
| Personal Characteristics | | | |
| 1. | Interviewer/facilitator | Which author/s conducted the interview or focus group? | 10 |
| 2. | Credentials | What were the researcher's credentials? **E.g. PhD, MD** | 10-11, S4 Table |
| 3. | Occupation | What was their occupation at the time of the study? | 10-11, S4 Table |
| 4. | Gender | Was the researcher male or female? | 10, S4 Table |
| 5. | Experience and training | What experience or training did the researcher have? | 10-11, S4 Table |
| Relationship with participants | | | |
| 6. | Relationship established | Was a relationship established prior to study commencement? | 10-11 |
| 7. | Participant knowledge of the interviewer | What did the participants know about the researcher? e**.g. personal goals, reasons for doing the research** | 11 |
| 8. | Interviewer characteristics | What characteristics were reported about the interviewer/facilitator? e.g. **Bias, assumptions, reasons and interests in the research topic** | 10-11, 22 |
| **Domain 2: study design** | | | |
| Theoretical framework | | | |
| 9. | Methodological orientation and Theory | What methodological orientation was stated to underpin the study? **e.g. grounded theory, discourse analysis, ethnography, phenomenology, content analysis** | 11, S4 Table |
| Participant selection | | | |
| 10. | Sampling | How were participants selected? **e.g. purposive, convenience, consecutive, snowball** | 10, Table 1 |
| 11. | Method of approach | How were participants approached? e**.g. face-to-face, telephone, mail, email** | 11 |
| 12. | Sample size | How many participants were in the study? | Table 1 |
| 13. | Non-participation | How many people refused to participate or dropped out? Reasons? | Table 1 |
| Setting | | | |
| 14. | Setting of data collection | Where was the data collected? e**.g. home, clinic, workplace** | 11 |
| 15. | Presence of non-participants | Was anyone else present besides the participants and researchers? | No |
| 16. | Description of sample | What are the important characteristics of the sample? **e.g. demographic data, date** | 10, Table 1 |
| Data collection | | | |
| 17. | Interview guide | Were questions, prompts, guides provided by the authors? Was it pilot tested? | 10-11, S3 Table |
| 18. | Repeat interviews | Were repeat interviews carried out? If yes, how many? | No |
| 19. | Audio/visual recording | Did the research use audio or visual recording to collect the data? | 11 |
| 20. | Field notes | Were field notes made during and/or after the interview or focus group? | 11 |
| 21. | Duration | What was the duration of the interviews or focus group? | 11 |
| 22. | Data saturation | Was data saturation discussed? | No |
| 23. | Transcripts returned | Were transcripts returned to participants for comment and/or correction? | No |
| **Domain 3: analysis and findings** | | | |
| Data analysis | | | |
| 24. | Number of data coders | How many data coders coded the data? | 11, S4 Table |
| 25. | Description of the coding tree | Did authors provide a description of the coding tree? | S4 Table |
| 26. | Derivation of themes | Were themes identified in advance or derived from the data? | 11, S4 Table |
| 27. | Software | What software, if applicable, was used to manage the data? | 11 |
| 28. | Participant checking | Did participants provide feedback on the findings? | No |
| Reporting | | | |
| 29. | Quotations presented | Were participant quotations presented to illustrate the themes / findings? Was each quotation identified? e**.g. participant number** | Yes (15-19, 22, S4 Appendix) |
| 30. | Data and findings consistent | Was there consistency between the data presented and the findings? | Yes (S4 Table, S4 Appendix) |
| 31. | Clarity of major themes | Were major themes clearly presented in the findings? | Yes (S4 Table, S4 Appendix) |
| 32. | Clarity of minor themes | Is there a description of diverse cases or discussion of minor themes? | Yes (S4 Table, S4 Appendix) |
