# Supplementary material for: G6PD testing and radical cure for Plasmodium vivax in Cambodia: A mixed methods implementation study
Source: PLoS One. 2022 Oct 20;17(10):e0275822. doi: 10.1371/journal.pone.0275822 (PMC9584508; doi:10.1371/journal.pone.0275822)
Supplement: S6 Table — (DOCX) [file pone.0275822.s006.docx]

**S6 Table:** Recommended* weight-based primaquine doses.

| Treatment course | Weight at presentation (kg) | Primaquine dose (mg) |
| --- | --- | --- |
| PQ8W (8-week primaquine)^†^ | 10 – 17 | 7.5 |
|  | 18 – 25 | 15.0 |
|  | 26 – 35 | 22.5 |
|  | 36 – 45 | 30.0 |
|  | 46 – 55 | 37.5 |
|  | 56 – 75 | 45.0 |
|  | ≥ 76 | 60.0 |
| PQ14 (14-day primaquine)^‡^ | 20 – 30 | 7.5 |
|  | 31 – 45 | 15.0 |
|  | 46 – 60 | 22.5 |
|  | 61 – 99 | 30.0 |

*Table adapted from guidelines provided to HC staff, designed by VIGTARC researchers.

^†^Primaquine dose to be taken once weekly for eight weeks by individuals with G6PDd, based on weight at presentation.

^‡^Primaquine dose to be taken once daily for 14 days by individuals with G6PDn, based on weight at presentation.
