# Supplementary material for: G6PD testing and radical cure for Plasmodium vivax in Cambodia: A mixed methods implementation study
Source: PLoS One. 2022 Oct 20;17(10):e0275822. doi: 10.1371/journal.pone.0275822 (PMC9584508; doi:10.1371/journal.pone.0275822)
Supplement: S7 Table — (DOCX) [file pone.0275822.s007.docx]

**S7 Table:** Coverage and results of different baseline haemoglobin tests in study population.

| **Category** | **Variable** | **Test type** | **Number tested (%)** | **Mean haemoglobin, g/dL [SD]** | **Prevalence of anaemia*, %  (95%CI)** |
| --- | --- | --- | --- | --- | --- |
| All participants (N=626) | | | | | |
| Haemoglobin testing | |  |  |  |  |
|  | No |  | 173 (27.6) | - | - |
|  | Yes | HemoCue® or STANDARD^TM †^ | 453 (72.4) | 13.3 [1.8] | 35.8 (31.5-40.3) |
|  |  | HemoCue® | 318 (50.8) | 13.3 [1.7] | 36.2 (31.0-41.6) |
|  |  | STANDARD^TM^ | 215 (34.3) | 13.2 [2.0] | 42.3 (35.9-49.1) |
| Male participants (N=576) | | | | | |
| Haemoglobin testing | |  |  |  |  |
|  | No |  | 173 (30.0) | - | - |
|  | Yes | HemoCue® or STANDARD^TM^ ^†^ | 403 (70.0) | 13.5 [1.8] | 33.3 (28.8-38.0) |
|  |  | HemoCue® | 306 (53.1) | 13.3 [1.7] | 34.6 (29.5-40.2) |
|  |  | STANDARD^TM^ | 176 (30.6) | 13.5 [1.9] | 40.3 (33.3-47.8) |
| Female participants (N=50) | | | | | |
| Haemoglobin testing | |  |  |  |  |
|  | No |  | 0 (0.0) | - | - |
|  | Yes | HemoCue® or STANDARD^TM †^ | 50 (100.0) | 12.0 [1.9] | 56.0 (42.1-69.0) |
|  |  | HemoCue® | 12 (24.0) | 11.5 [1.4] | 75.0 (44.7-91.8) |
|  |  | STANDARD^TM^ | 39 (78.0) | 12.0 [2.1] | 51.3 (35.9-66.4) |

*According to World Health Organization criteria for anaemia in adult males (haemoglobin <13.0 g/dL) and adult non-pregnant females (haemoglobin <12.0 g/dL) [35].

^†^For participants where both investigations were used, HemoCue® result was selected preferentially for analysis of mean haemoglobin and anaemia prevalence.
