# Supplementary material for: G6PD testing and radical cure for Plasmodium vivax in Cambodia: A mixed methods implementation study
Source: PLoS One. 2022 Oct 20;17(10):e0275822. doi: 10.1371/journal.pone.0275822 (PMC9584508; doi:10.1371/journal.pone.0275822)
Supplement: S1 Fig — (DOCX) [file pone.0275822.s008.docx]

**S2 Figure:** The total number of G6PD tests performed, by month, and the proportion that were performed on patients from a villages within the catchment area of the HC, outside the catchment area, or whose villages are unknown (undocumented).

G6PD = glucose-6-phosphate dehydrogenase. HC = health center.
