# Supplementary material for: G6PD testing and radical cure for Plasmodium vivax in Cambodia: A mixed methods implementation study
Source: PLoS One. 2022 Oct 20;17(10):e0275822. doi: 10.1371/journal.pone.0275822 (PMC9584508; doi:10.1371/journal.pone.0275822)
Supplement: S2 Fig — Number of P.v episodes and G6PD testing rate by month, for A) Kravanh HC, B) Promoy HC, C) Samrong HC, and D) Prongil HC. (DOCX) [file pone.0275822.s009.docx]

**S1 Figure:** Number of *P.v* episodes and G6PD testing rate by month, for A) Kravanh HC, B) Promoy HC, C) Samrong HC, and D) Prongil HC.

A)


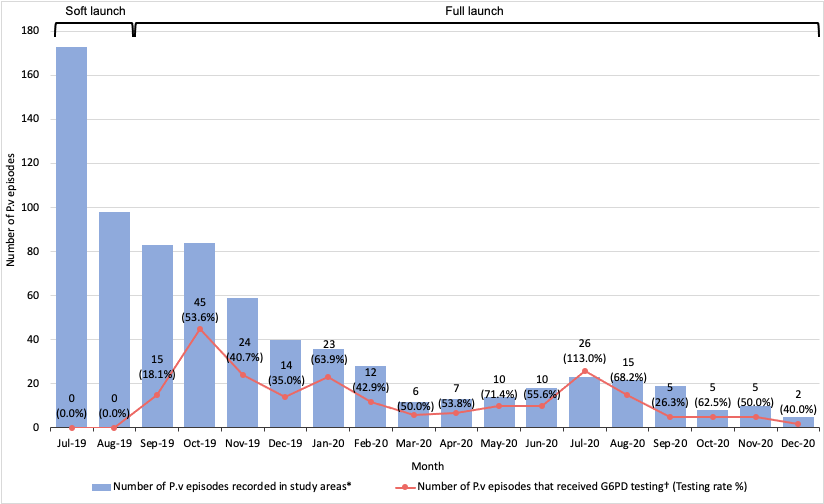


B)


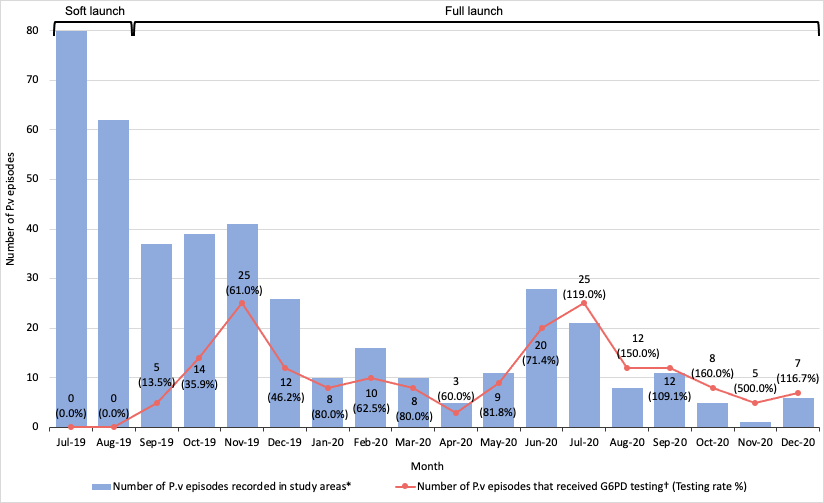


C)


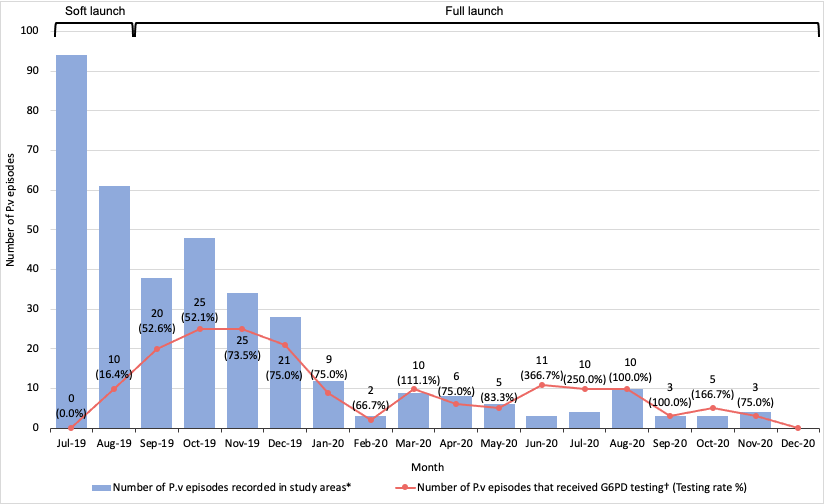


D)


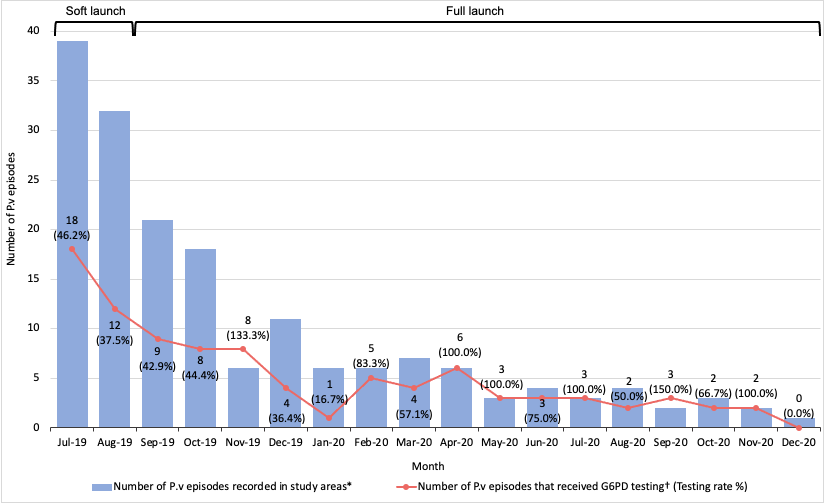


P.v = *Plasmodium vivax*. G6PD = glucose-6-phosphate dehydrogenase. HC = health center.

*According to national MIS data.

^†^G6PD testing using qualitative test (CareStart^TM^ rapid diagnostic test) for males and quantitative test (CareStart^TM^ Biosensor or STANDARD^TM^ Biosensor) for females.
