# Supplementary material for: G6PD testing and radical cure for Plasmodium vivax in Cambodia: A mixed methods implementation study
Source: PLoS One. 2022 Oct 20;17(10):e0275822. doi: 10.1371/journal.pone.0275822 (PMC9584508; doi:10.1371/journal.pone.0275822)
Supplement: S3 Fig — (DOCX) [file pone.0275822.s010.docx]

**S3 Figure: Comparison of qualitative and quantitative G6PD test results.**

197 male participants received both qualitative and quantitative G6PD testing. Qualitative testing was with CareStart^TM^ rapid diagnostic test. Quantitative testing was performed with either STANDARD^TM^ Biosensor, or CareStart^TM^ Biosensor with separate haemoglobin testing (using HemoCue^®^) to provide results in U/g Hb (units per gram of haemoglobin). Fig A shows the frequency distribution of quantitative results for different qualitative outcomes.

**Fig A:** Violin plot showing results of 197 male patients who received both quantitative and qualitative G6PD testing.

6 U/g Hb threshold used to define “normal” G6PD activity

CareStart^TM^ Biosensor test results.

STANDARD^TM^ Biosensor test results.

Mean average.

Box and whisker plots indicate median and interquartile range.

G6PD = glucose-6-phosphate dehydrogenase.

*Obtained using quantitative G6PD tests (CareStart^TM^ Biosensor combined with haemoglobin measurement from HemoCue^®^ test or STANDARD^TM^ Biosensor).

^†^Obtained using qualitative CareStart^TM^ rapid diagnostic test, which detects ≥30% G6PD activity as “normal” and <30% G6PD activity as “deficient”.
