# Supplementary material for: G6PD testing and radical cure for Plasmodium vivax in Cambodia: A mixed methods implementation study
Source: PLoS One. 2022 Oct 20;17(10):e0275822. doi: 10.1371/journal.pone.0275822 (PMC9584508; doi:10.1371/journal.pone.0275822)

**S1 Appendix:** Healthcare worker training and education materials, and methods of delivery.

Training and education were provided for healthcare workers and project staff. Schedule of training and workshop events delivered to staff are shown in Table A. Educational materials designed and utilised included a poster showing instructions for qualitative G6PD test use (Fig A), table of cut-off values to interpret quantitative G6PD tests (Table B), a treatment flow chart (Fig B) and a script to advise HC staff on how to counsel patients when initiating primaquine (Fig C). In addition, a more extensive 40-page manual which detailed diagnosis and interpretation of G6PD test results and management of patients within the care pathway was written and distributed (Fig D).

**Table A:** Schedule of training events and workshops for healthcare workers and project staff.

| **Event** | **Date/ frequency** | **Duration and location** | **Number and type of participants** | **Description/Objective** |
| --- | --- | --- | --- | --- |
| App training | 15^th^ – 18^th^ January 2020 | 1-day session meeting held at each of the 4 HCs | 71 VMWs  2 CNM staff  2 Project staff and 2 HC staff join the training held at each of the 4 HCs. | - Training on the use of the *P.v* app on mobile phones and tablets by VMWs and HC staff |
| SD Biosensor training | 16^th^ March 2020 | 1-day session meeting held at each of the 4 HCs | 30 HC staff  6 PHD/OD staff  2 Project staff  2 Other partners. | - Conduct training on the G6PD testing using the SD G6PD Biosensor analyser machine for health facilities workers as well as counterpart and partners. |
| Review and evaluation of tools, SOP and framework | 15^th^ June 2020 | 1-day session meeting held at each of the 4 HCs | 16 HC staff  4 PHD and OD staff  6 Project staff  7 Other partners. | - Refresher training on primaquine, G6PD testing, follow-up and use of the tablet/smartphone app - Feedback from users. |
| Provincial technical working group for Health | Regular-Monthly | Held at the PHD office | 10 PHD  20 Local and International NGO partners. | - Organised by the PHD to have an update on malaria control and elimination. |
| VMW bi-monthly meetings | Bi-monthly regularly | 1-day session meeting held at each of the 4 HCs | 71 VMWs and  2 HC staff join the meeting held at each of the 4 HCs. | - Collect malaria data - Verify RDT use - Supply RDTs, ASMQ and IEC materials - Refresher training and support on different aspects of care pathway and use of smartphone app. |
| Community mobilisations | Regular-Monthly | 1-day session meeting held at each villages of the 4 HCs. | 2 VMW/MMWs  1 HC staff  2 local authorities join the meeting held at each of the 4 HCs. | - Monitoring, coaching and discussion with HC/ local authorities and VMW/MMWs to share information on *P.v* radical cure study in their HC catchment areas. - Based on the current lesson learnt, for patients who repeatedly positive for *P.v* are being actively passion information on *P.v* radical cure study to their neighbourhoods and their community. |
| Field supervisions | Quarterly | 1-2-day session field visit held at each village of the 4 HCs. | 1 Central HSD, 1 Project staff and 2 CNM staff. | - Monitoring, coaching and discussion with project team, OD, HC and VMW/MMWs. - In term of follow up treatment side effect, G6PD testing, DOT follow up by using mobile/smart phone by VMW and tablet by health centres staff. |

HC = health centre. *P.v* = *Plasmodium vivax.* VMW = village malaria worker. CNM = National Malaria Center of Cambodia. PHD = provincial health department. SD = STANDARD^TM^. G6PD = glucose-6-phosphate dehydrogenase. SOP = standard operating protocol. NGO = Non-governmental organisation. RDT = rapid diagnostic test. ASMQ = artesunate-mefloquine. IEC = information, education and communication. MMW = mobile malaria worker. OD = operational district. DOT = directly observed therapy.

**Fig A:** 2-Page document/poster showing instructions of how to use G6PD RDTs, for HC staff.
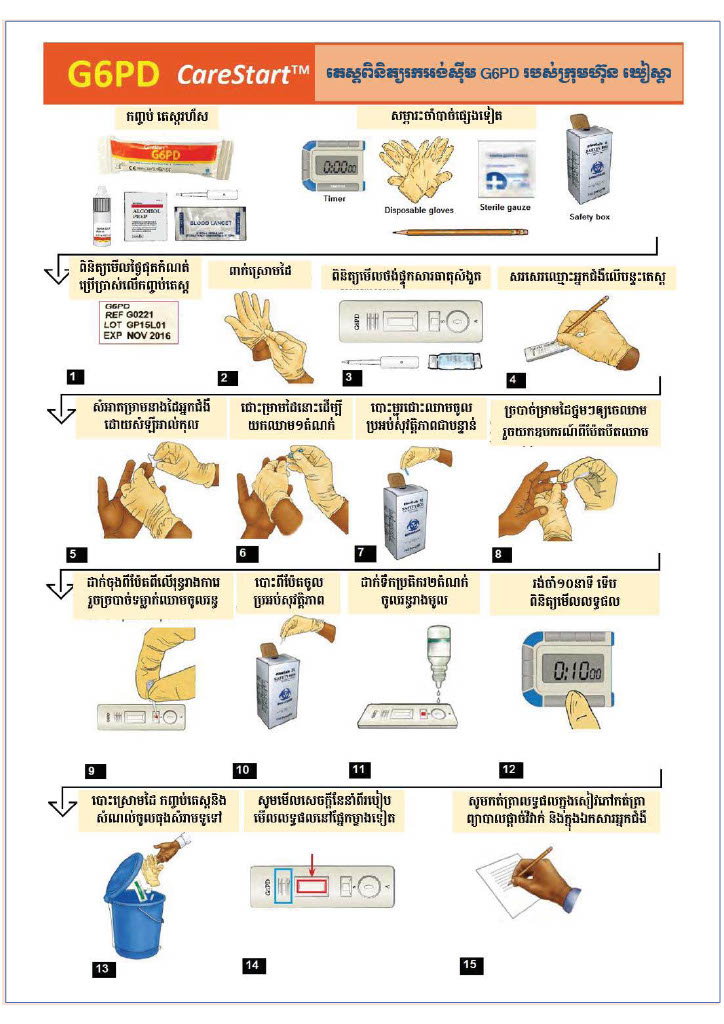

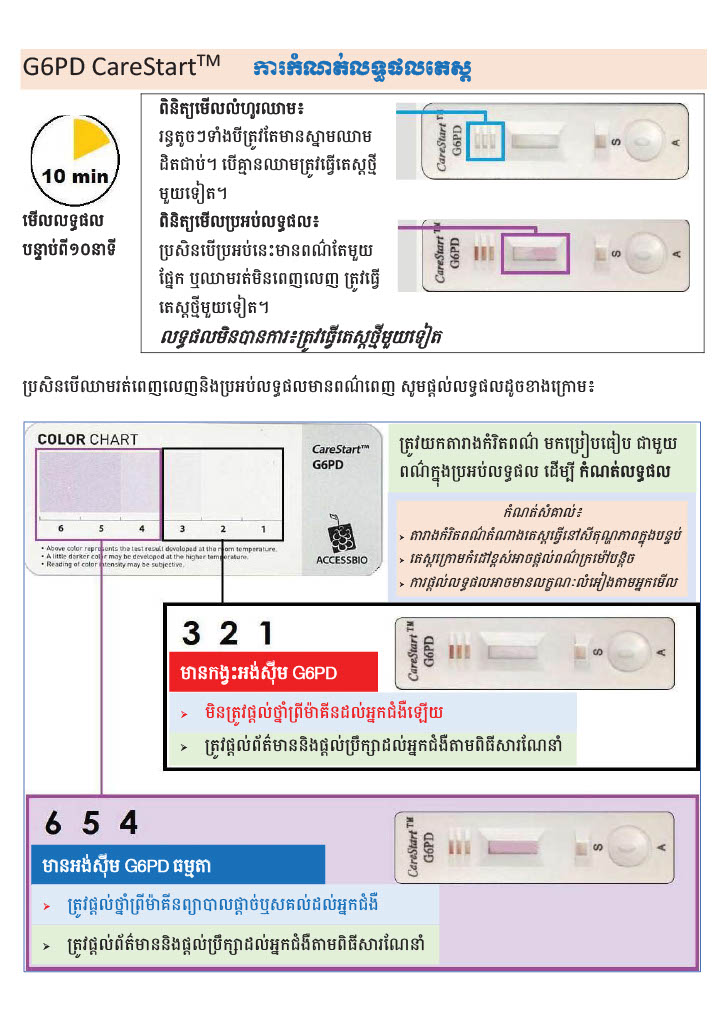


**Table B:** G6PD activity thresholds used to determine whether quantitative test results should be interpreted as normal, intermediate, or deficient G6PD status based on the thresholds in the decision of the CNM technical working group.

| Test types | Patient sex | Threshold value(s) for G6PD status (u/g Hb) | | |
| --- | --- | --- | --- | --- |
|  |  | Normal G6PD status (equivalent to ≥80% activity) | Intermediate G6PD status (equivalent to 30-80% activity) | Deficient G6PD status (equivalent to <30% activity) |
| Quantitative G6PD tests, including STANDARD^TM^ Biosensor and CareStart^TM^ Biosensor | Male | ≥6.0 | N/A* | ≤5.9 |
|  | Female | ≥6.0 | 4.0 – 5.9 | ≤3.9 |

G6PD = glucose-6-phosphate dehydrogenase. u/g Hb = units per gram of haemoglobin. N/A = not applicable.

*Males do not exhibit intermediate activity but are only classified as normal or deficient G6PD activity as a result of X-linked disease aetiology.

**Fig B:** Poster of treatment flow chart, designed to guide HC staff.

**Fig C:** Script for counselling of patients, designed for HC staff to use prior to primaquine initiation.
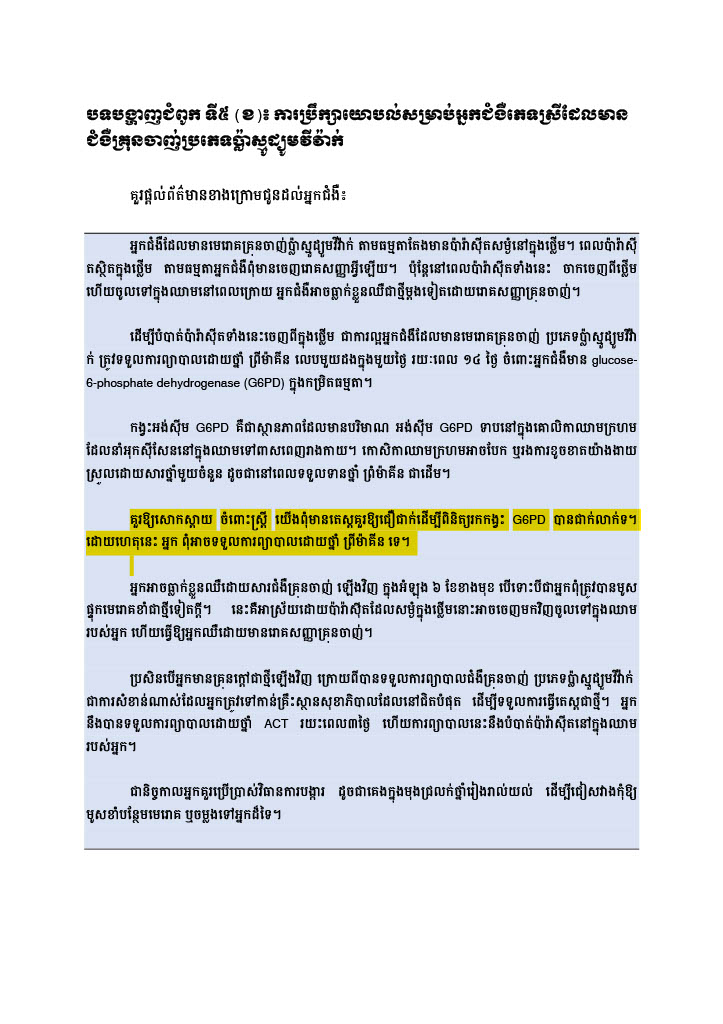

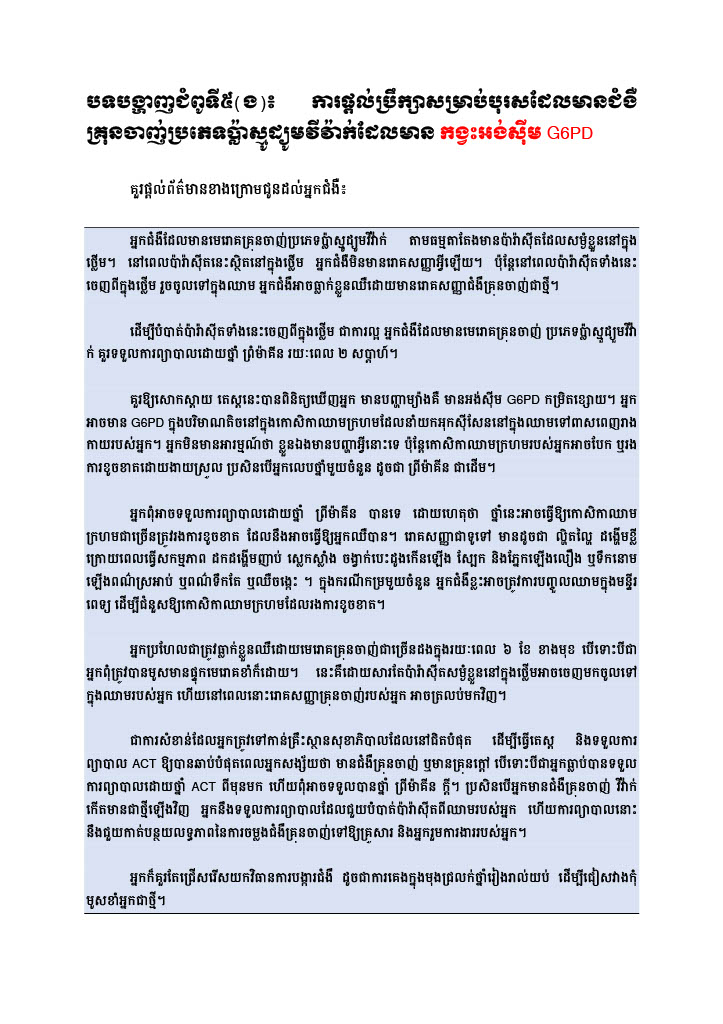

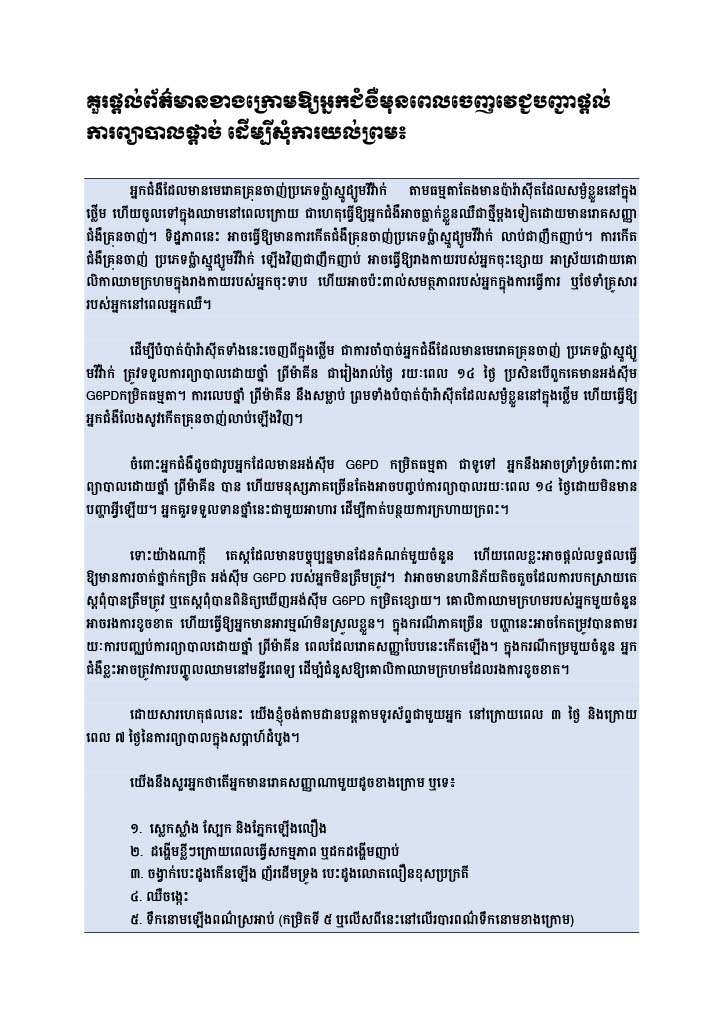

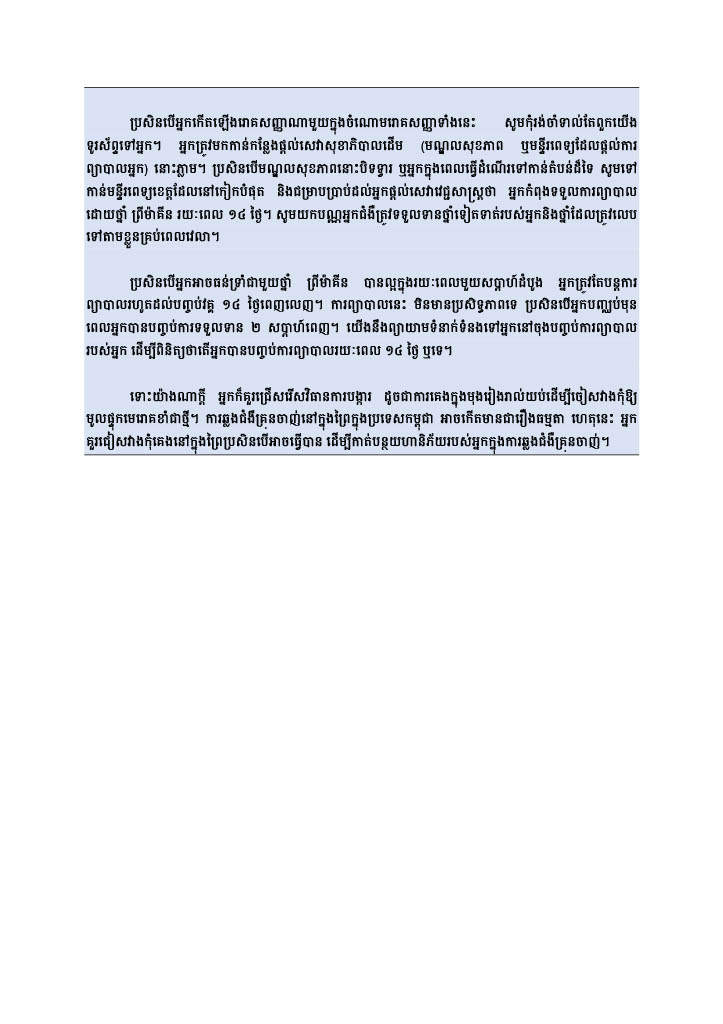


Fig D: Manual for diagnosis and interpretation of G6PD test results, and management of patients within the care pathway.
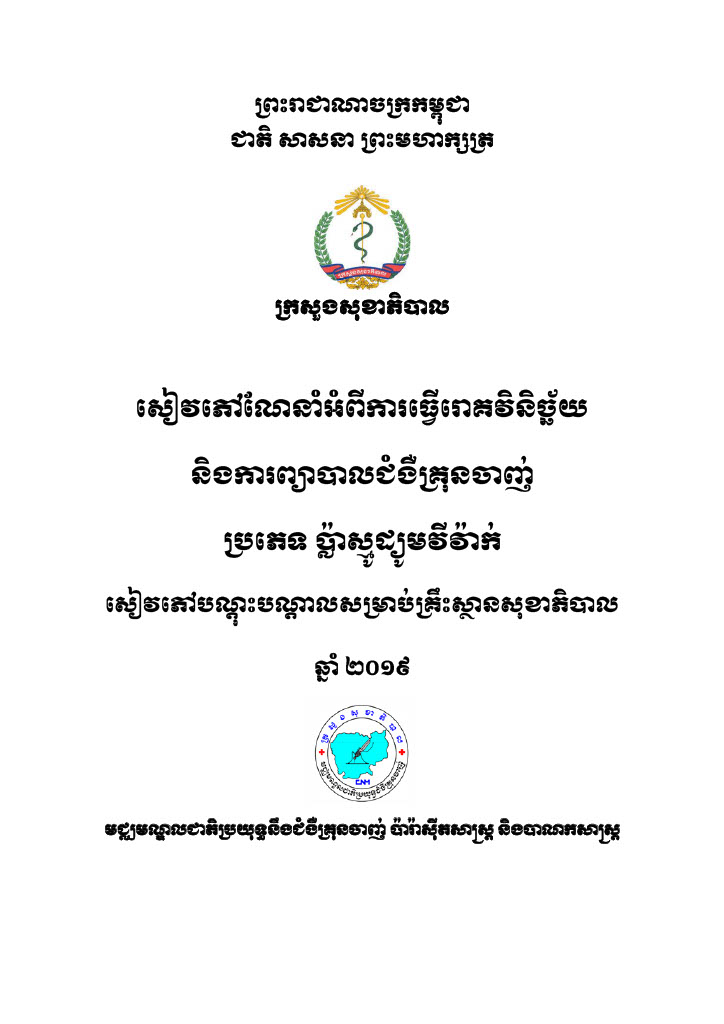

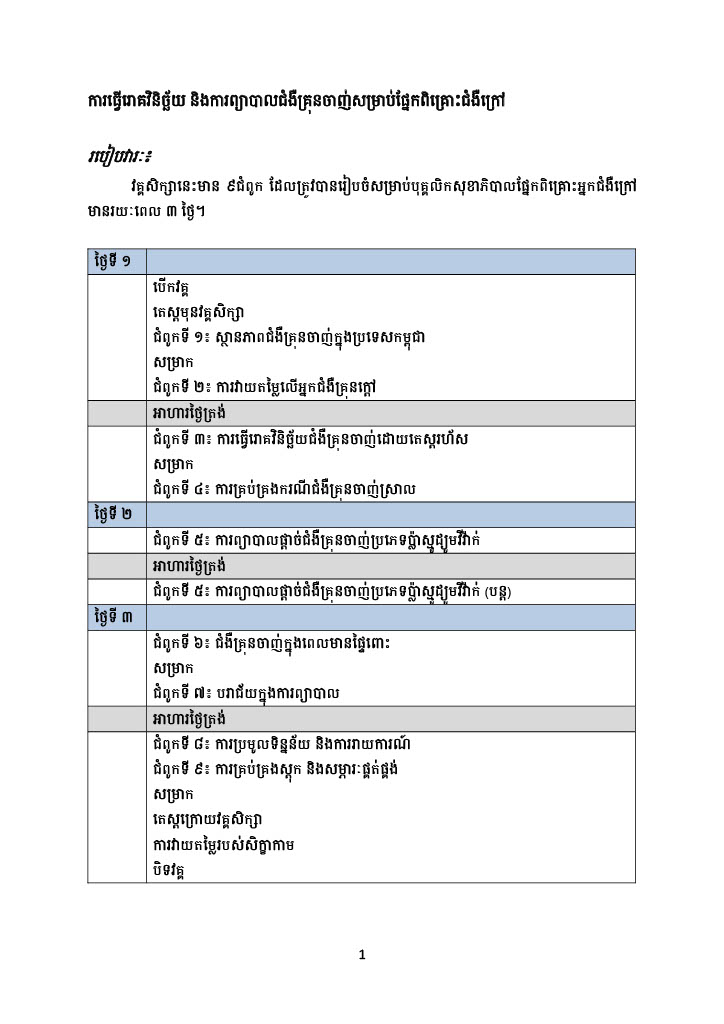

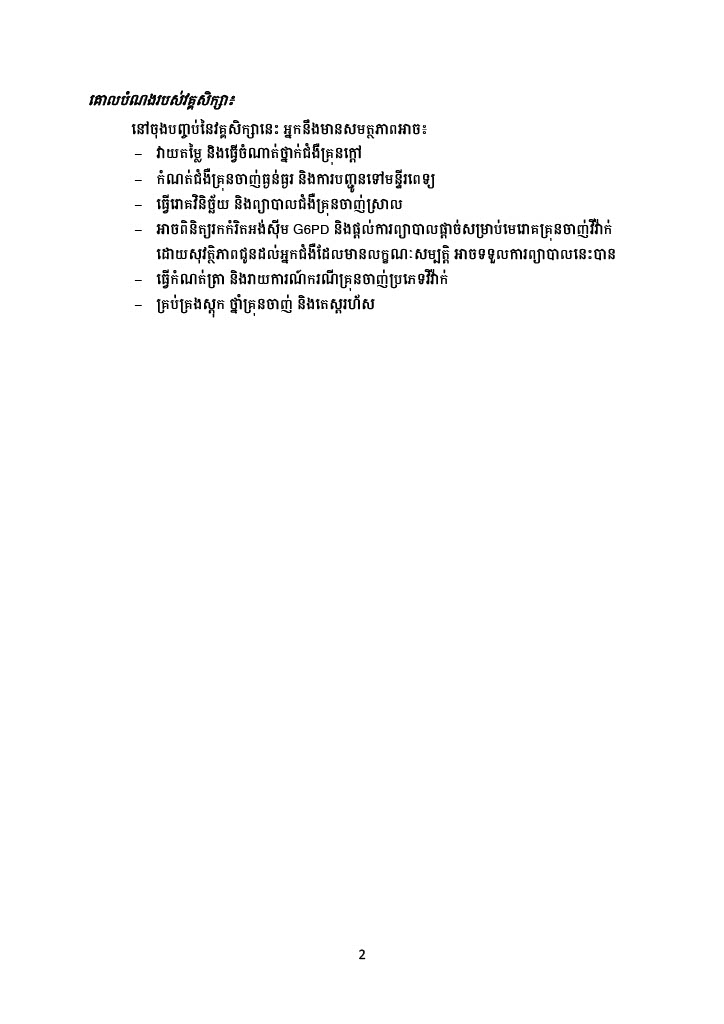

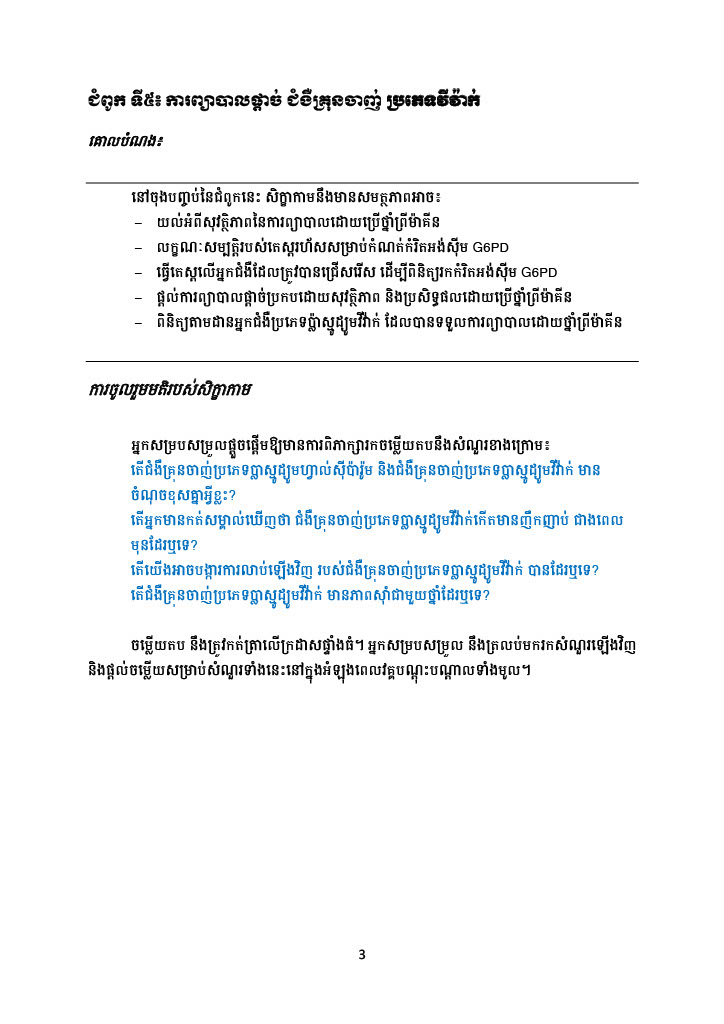

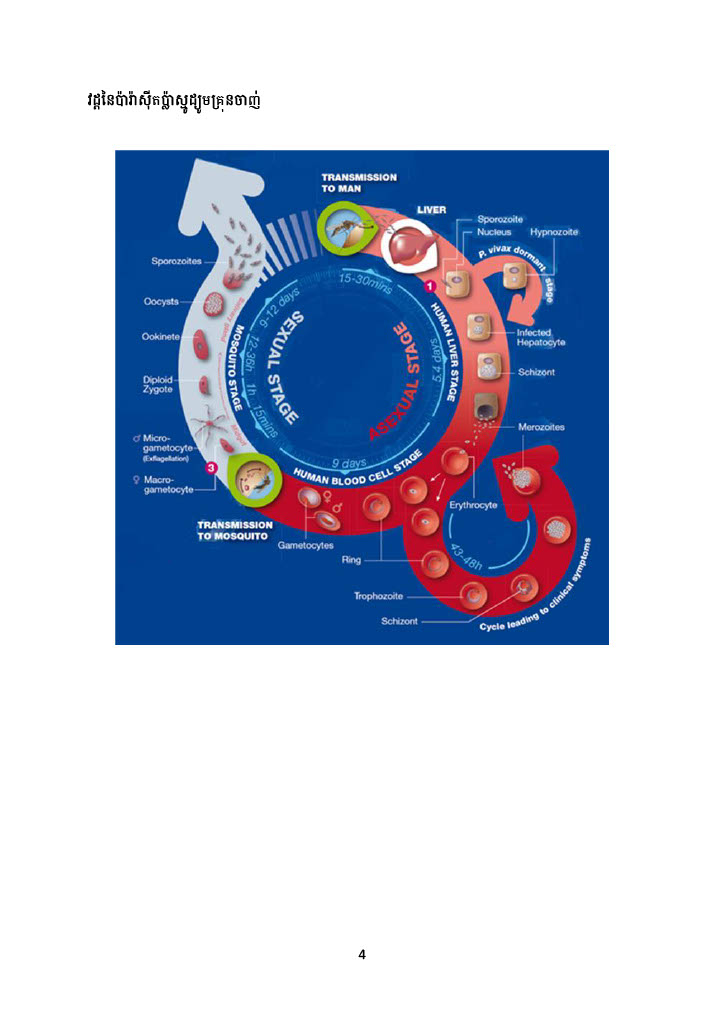

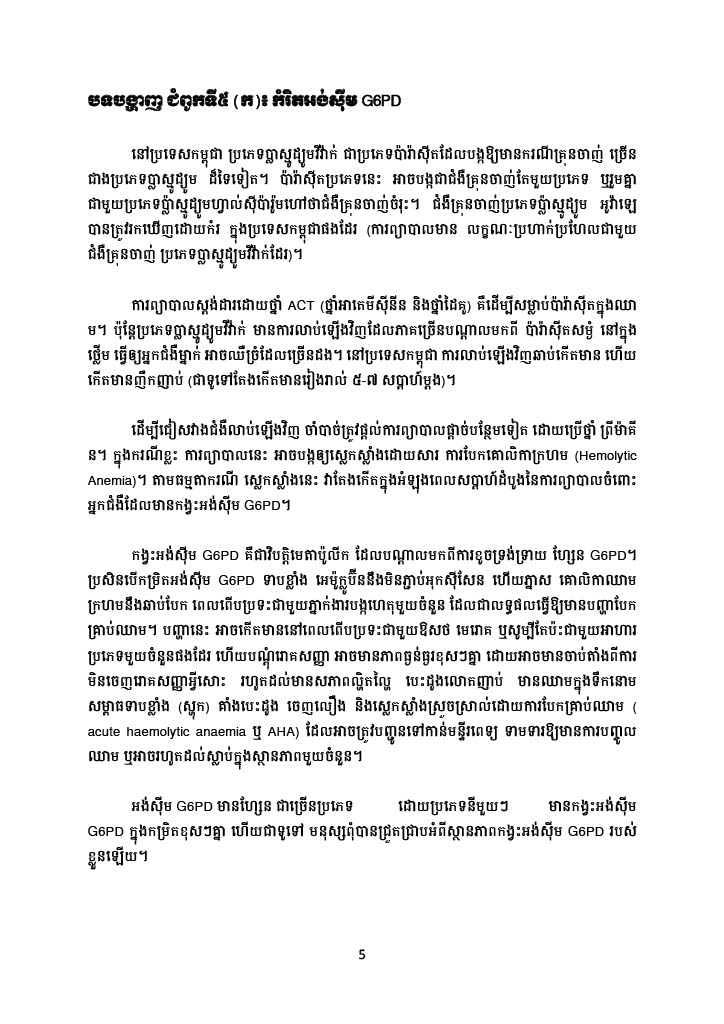

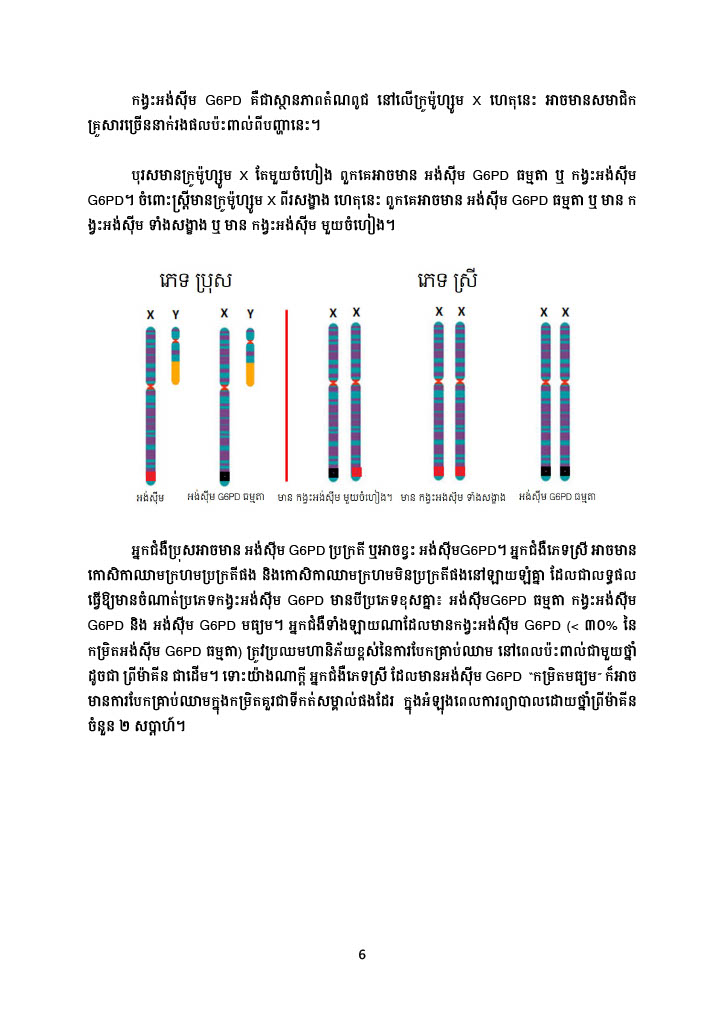

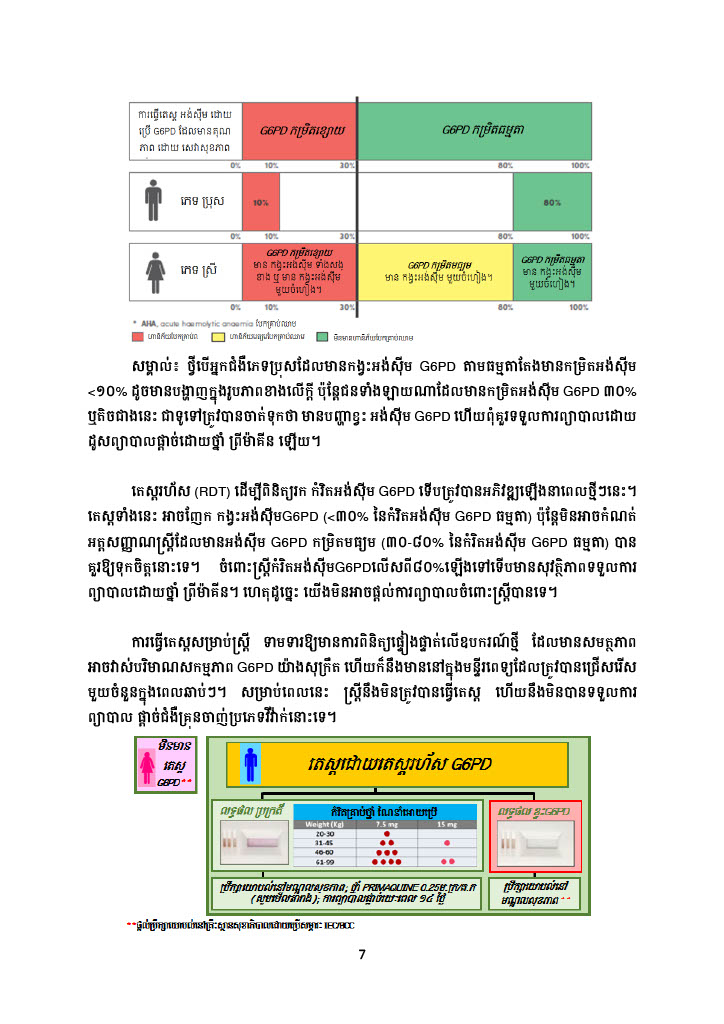

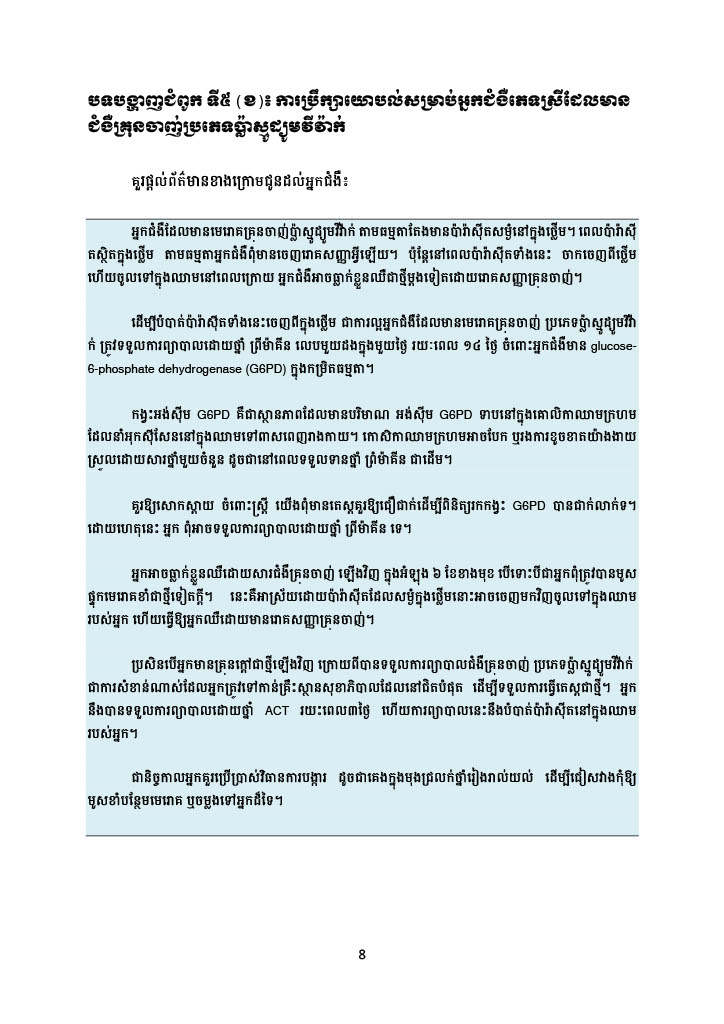

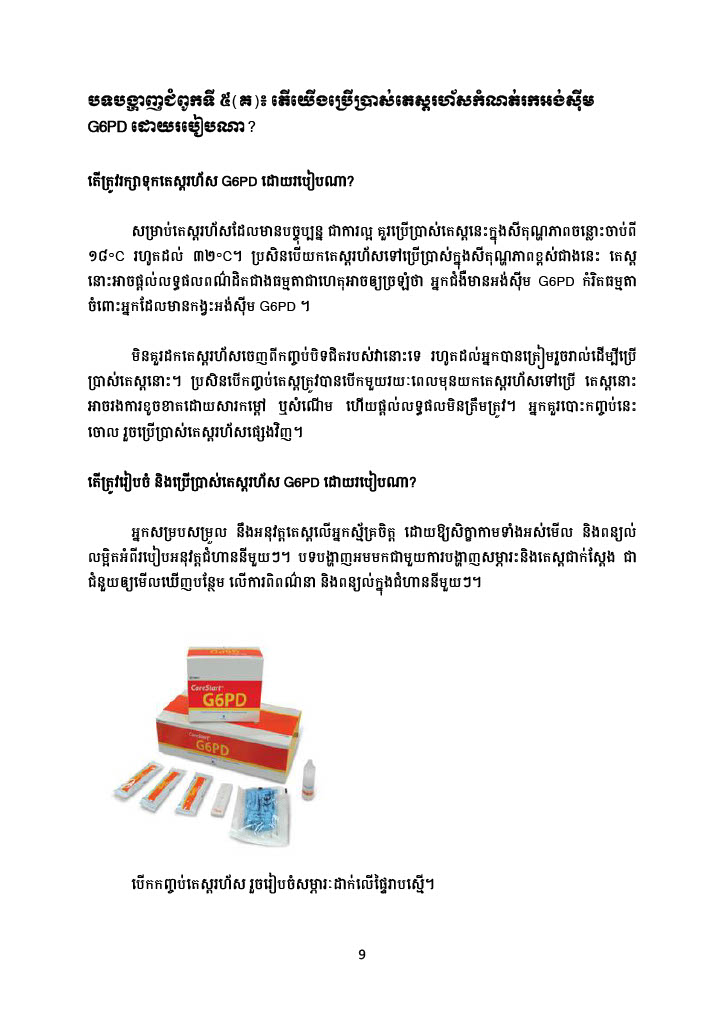

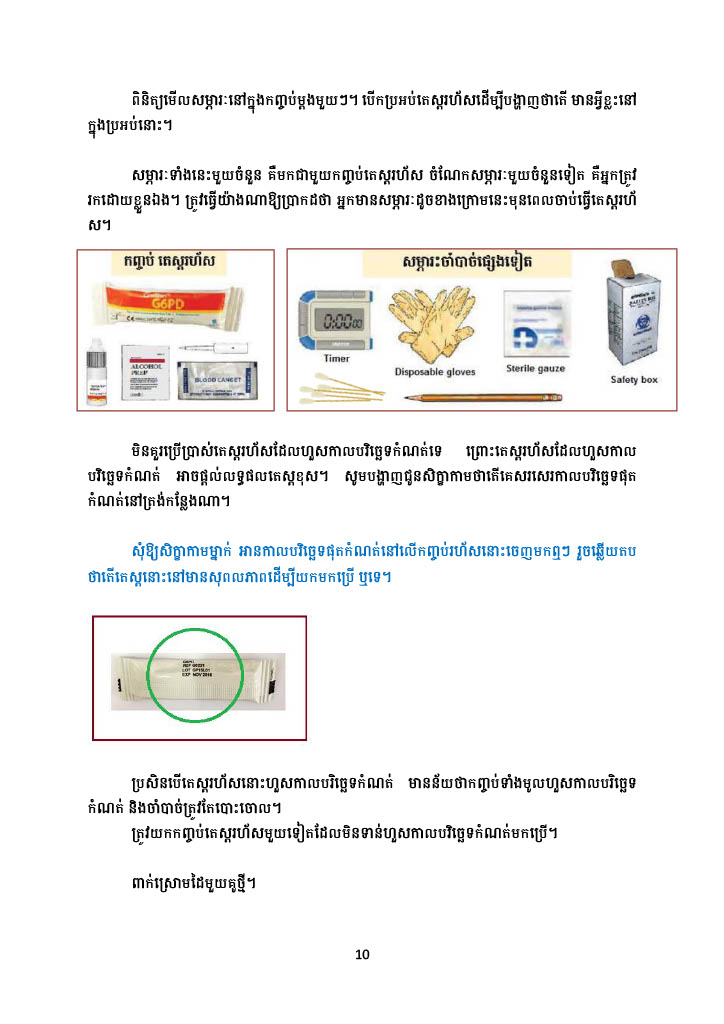

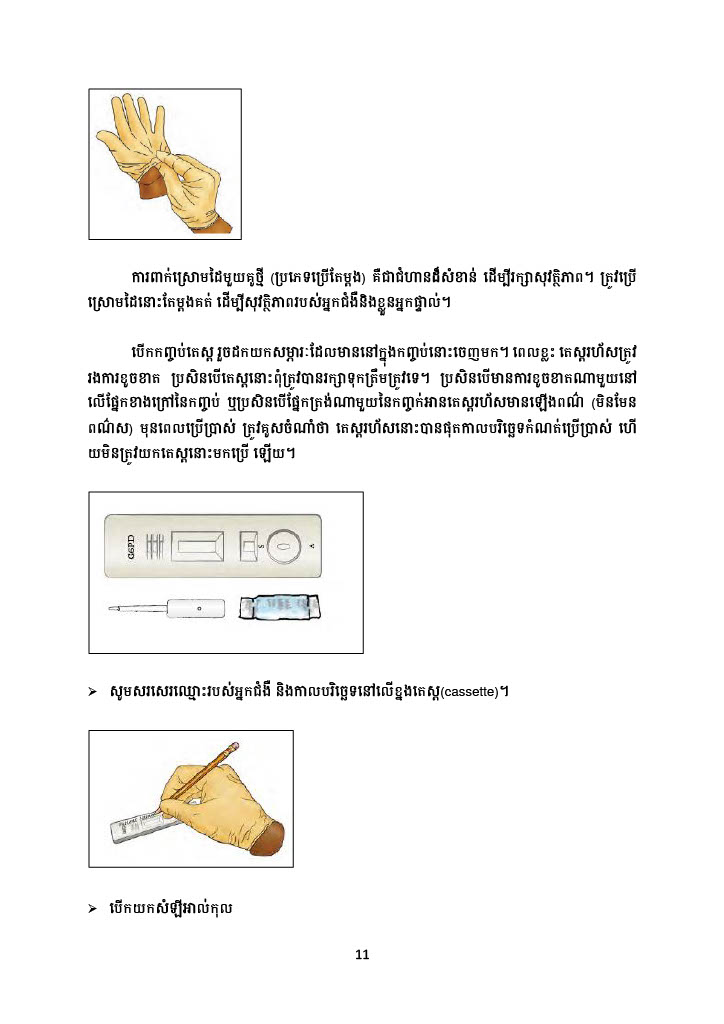

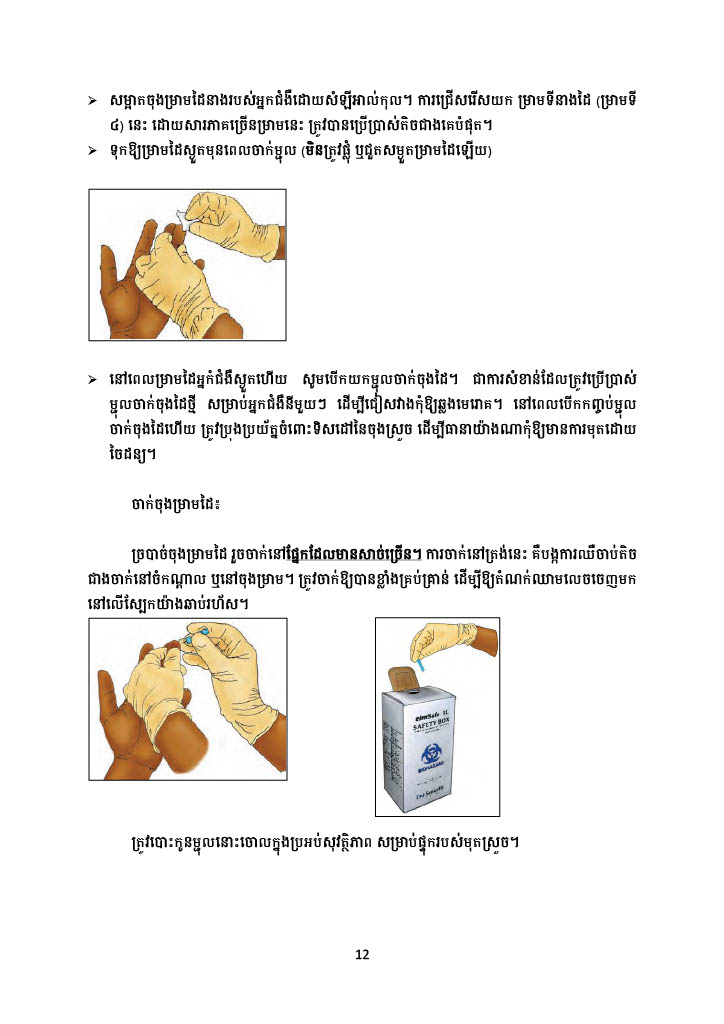

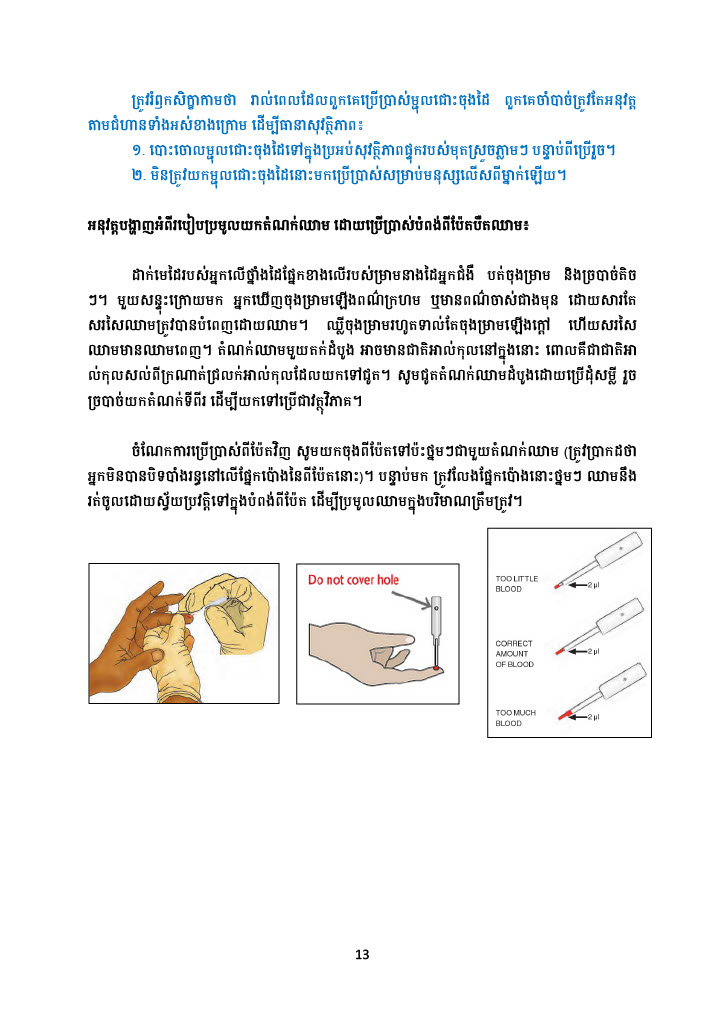

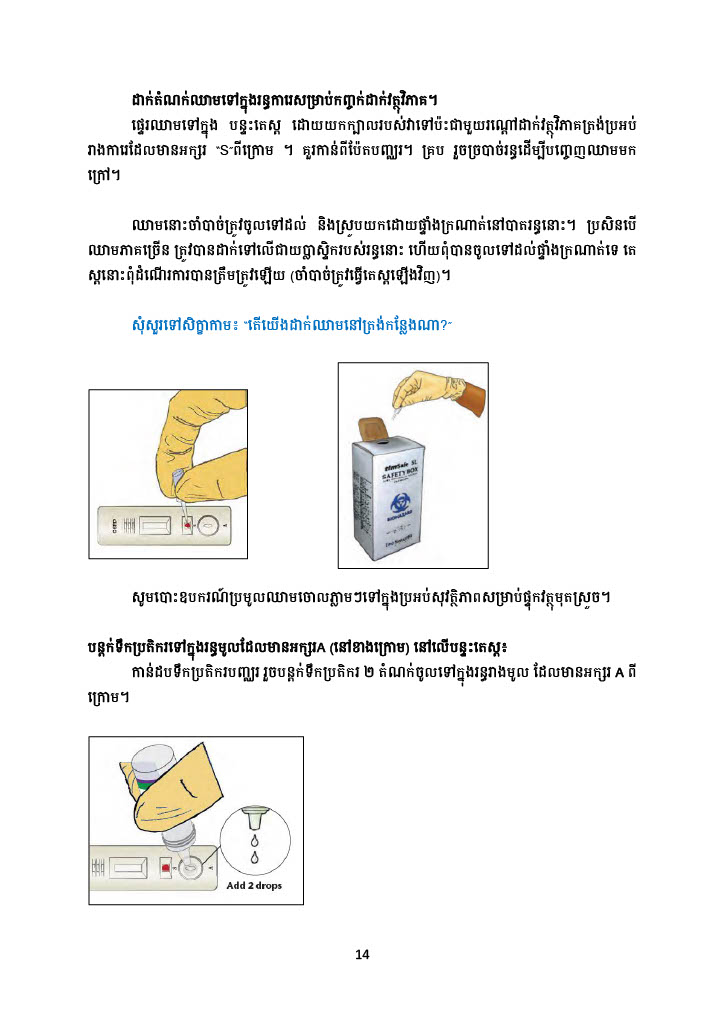

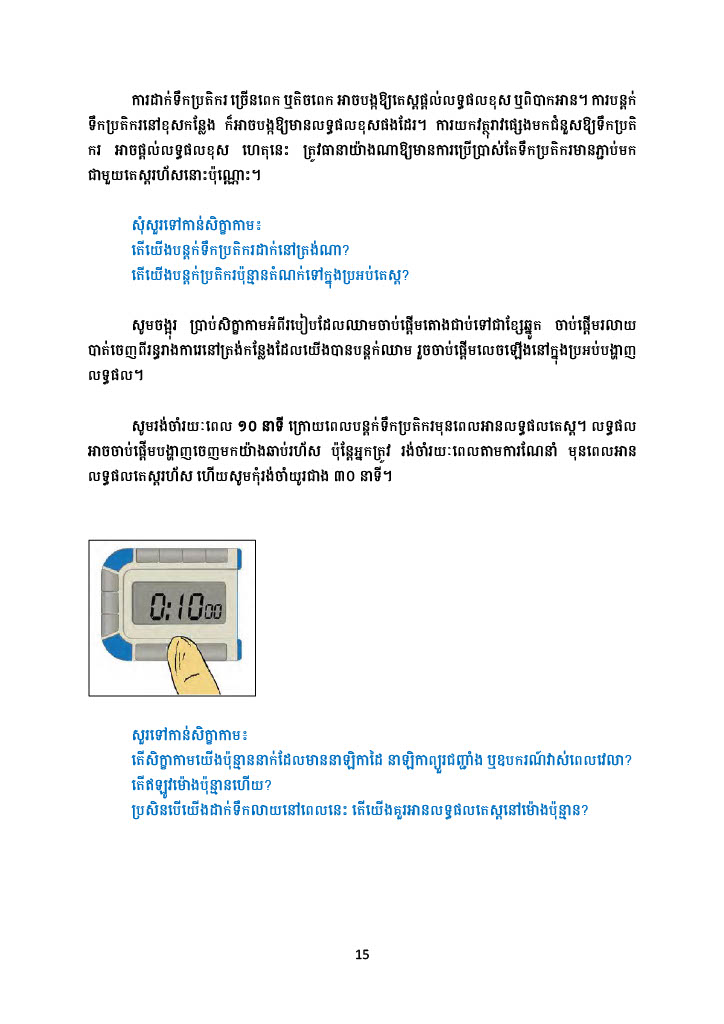

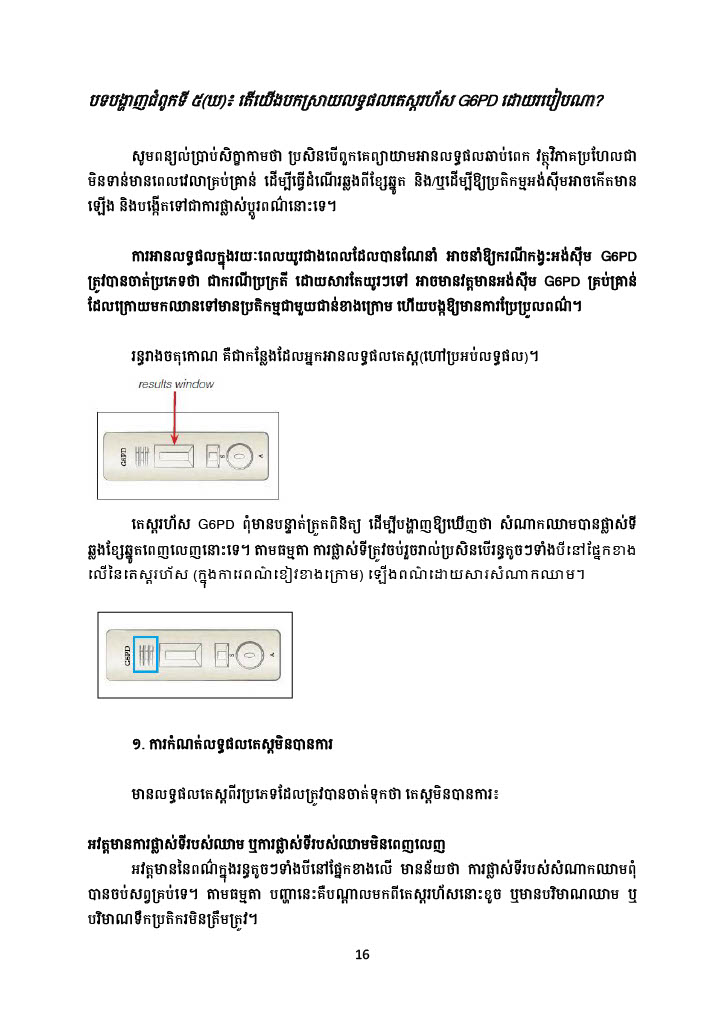

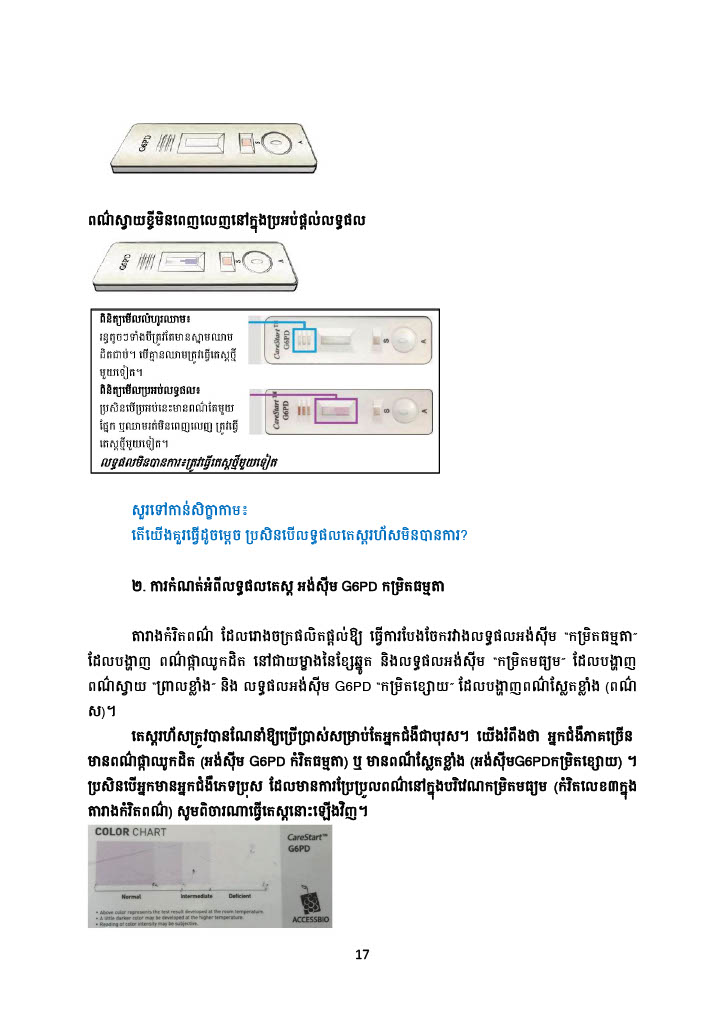

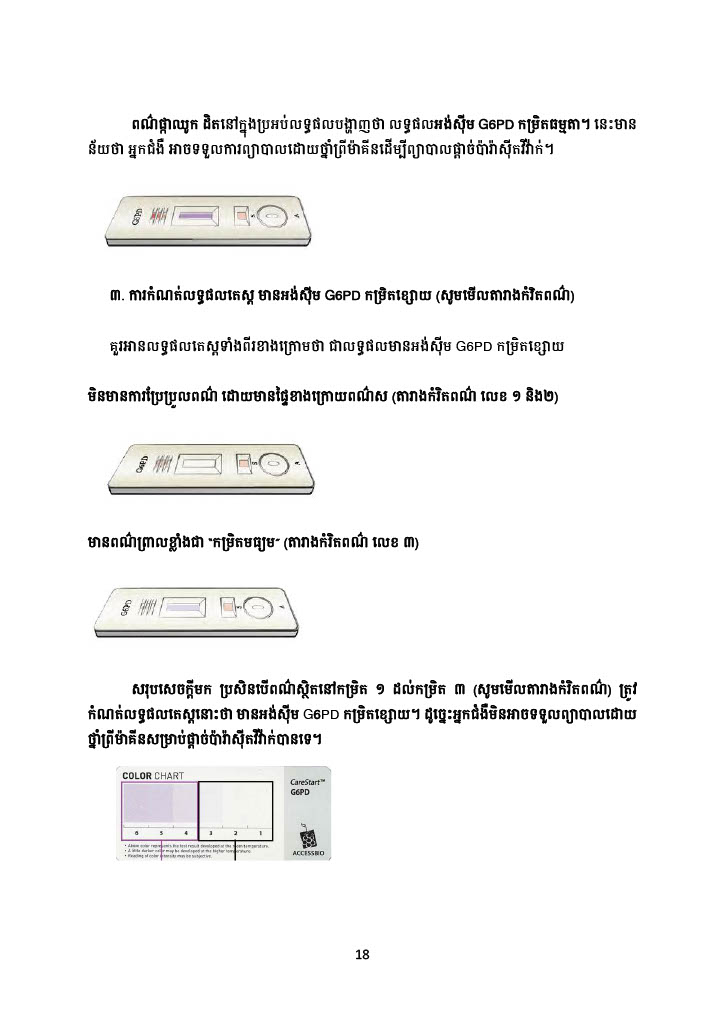

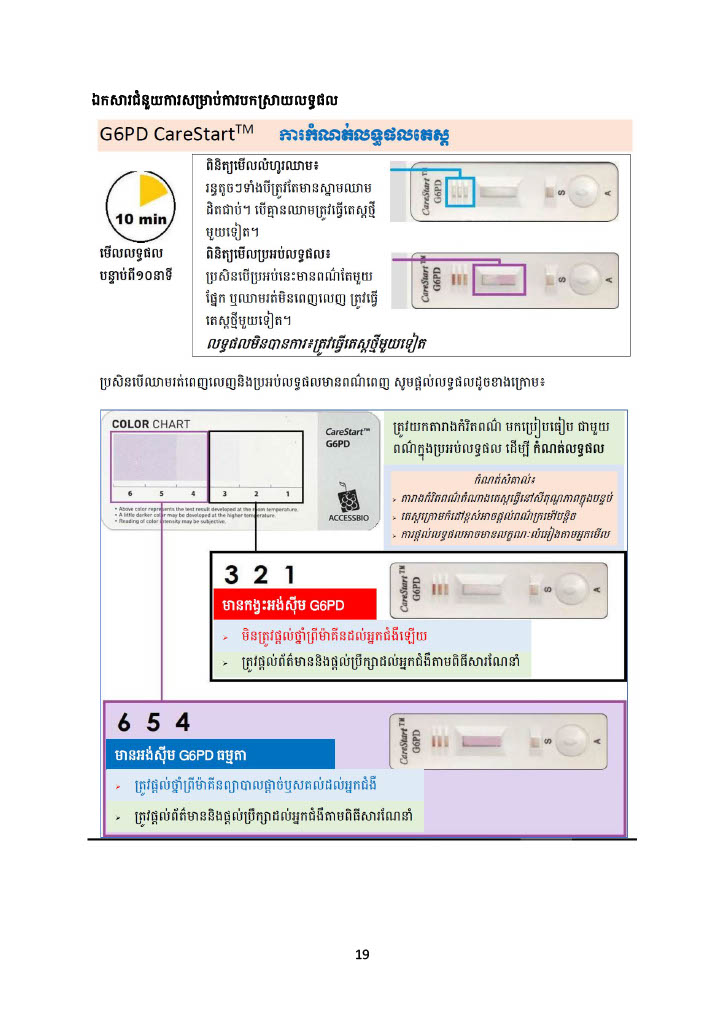

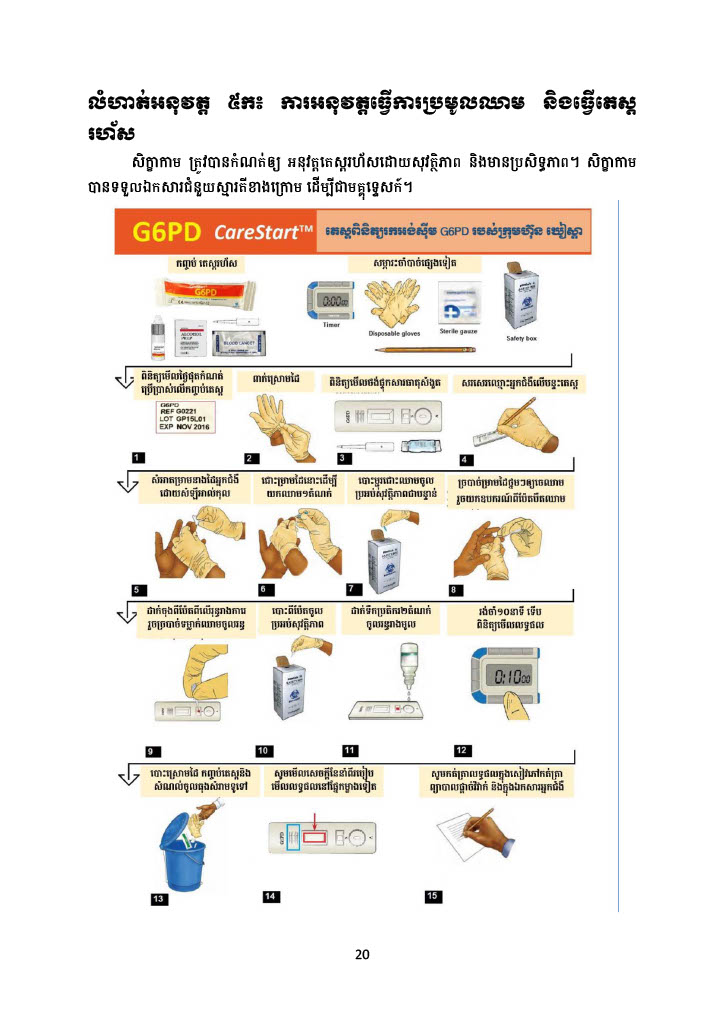

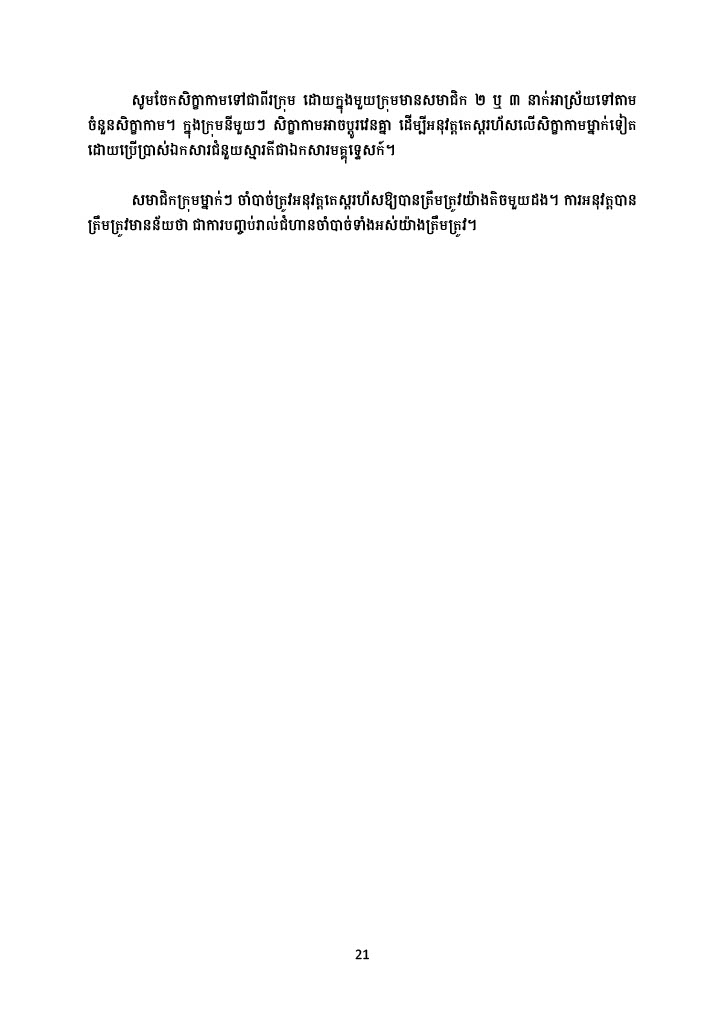

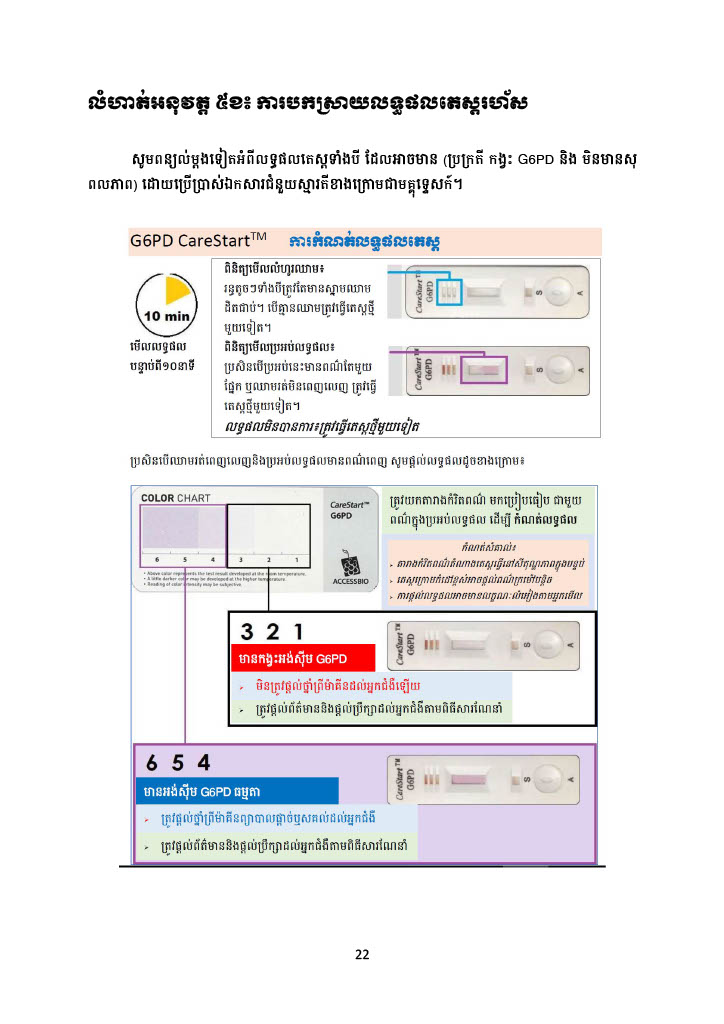

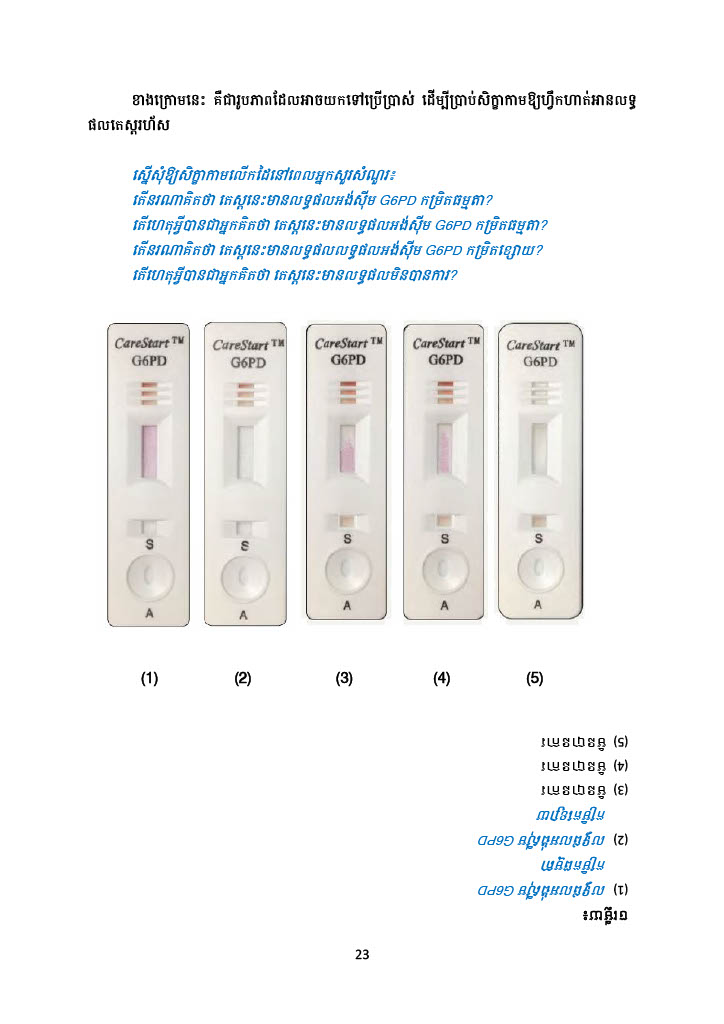

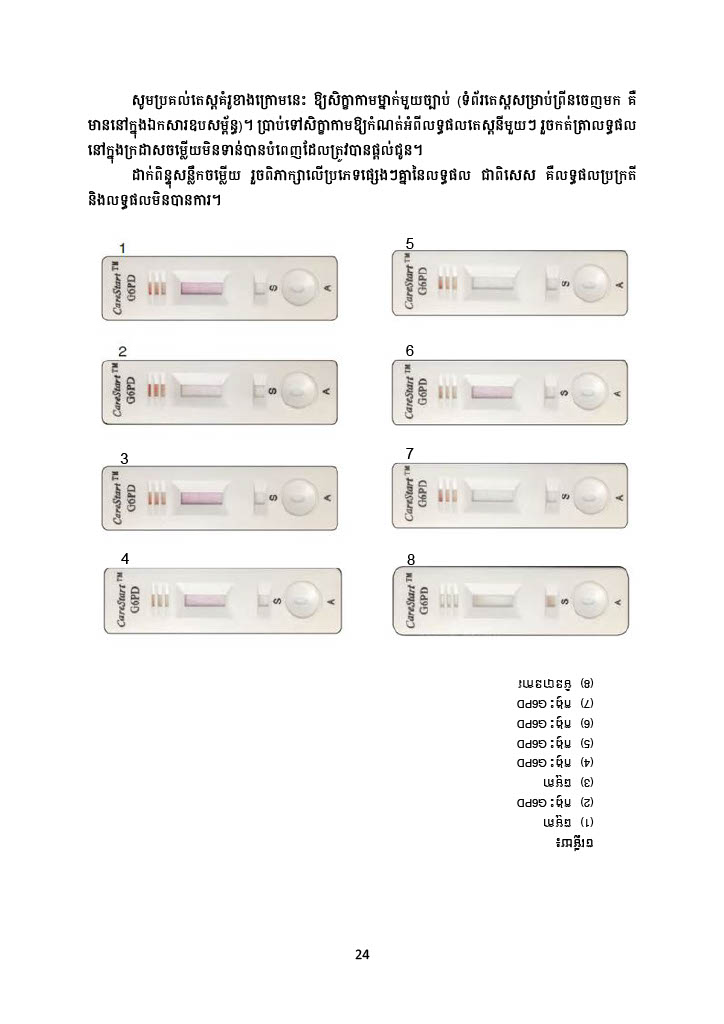

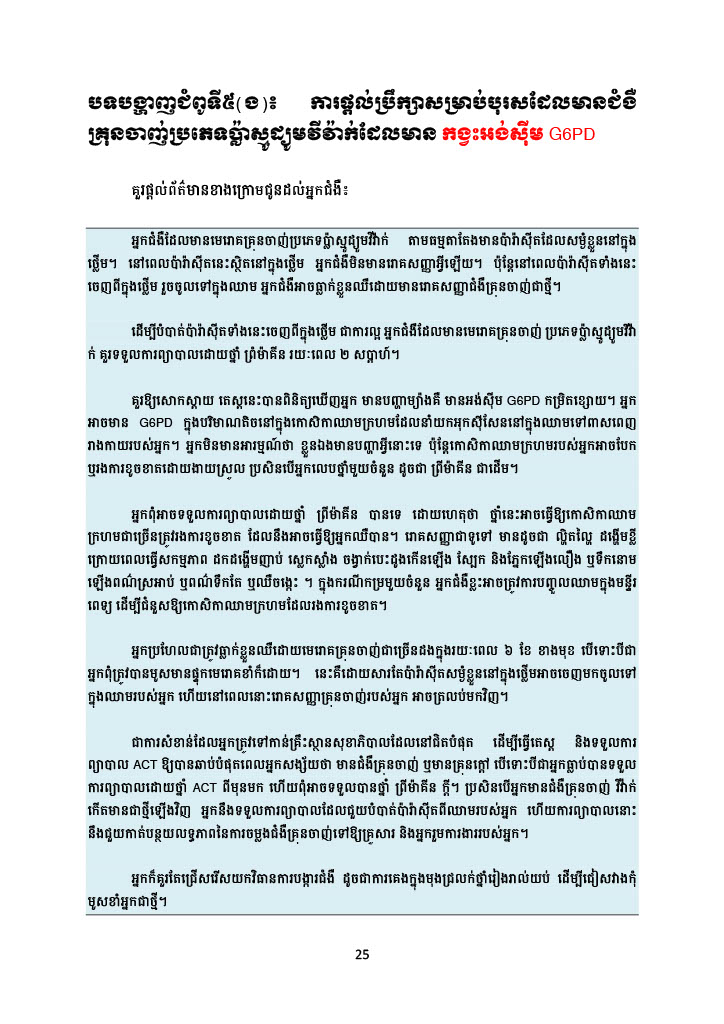

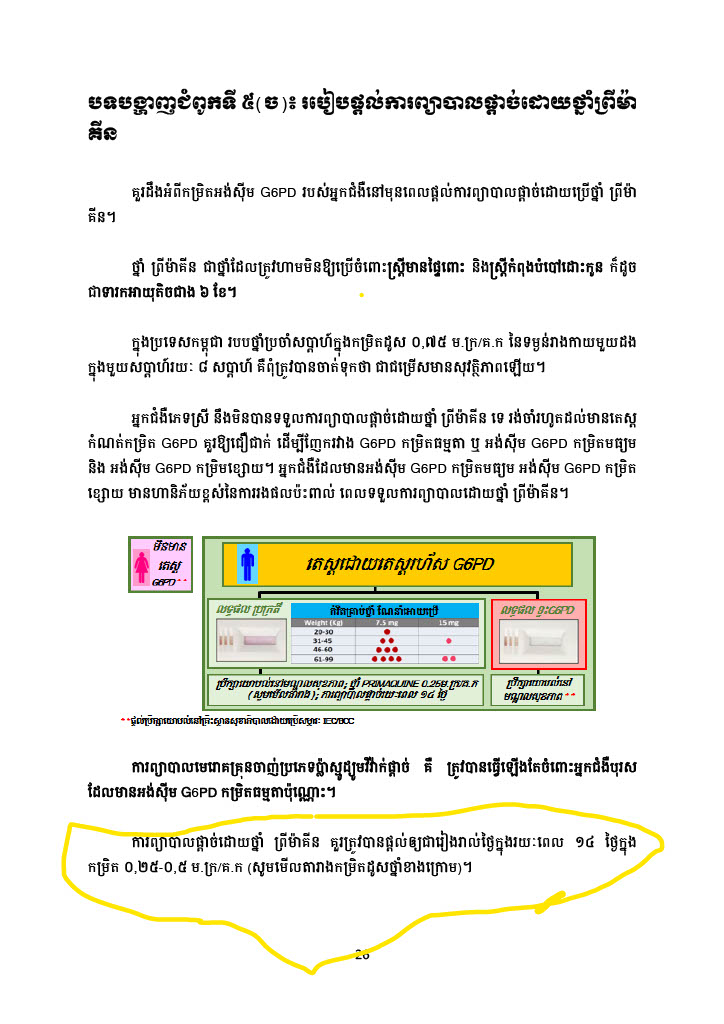

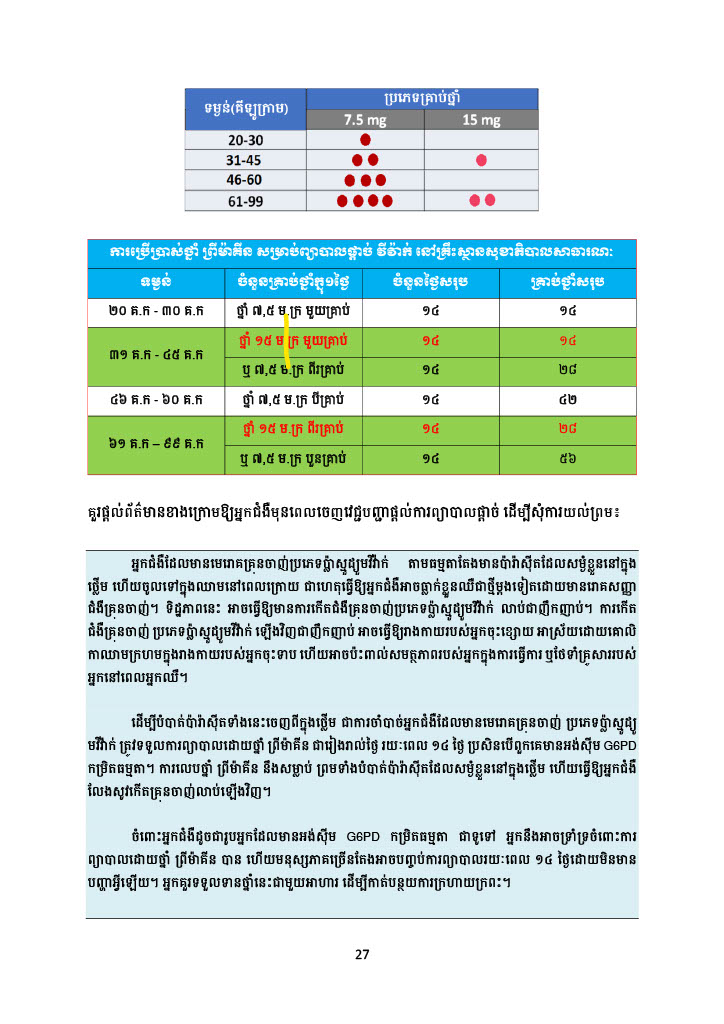

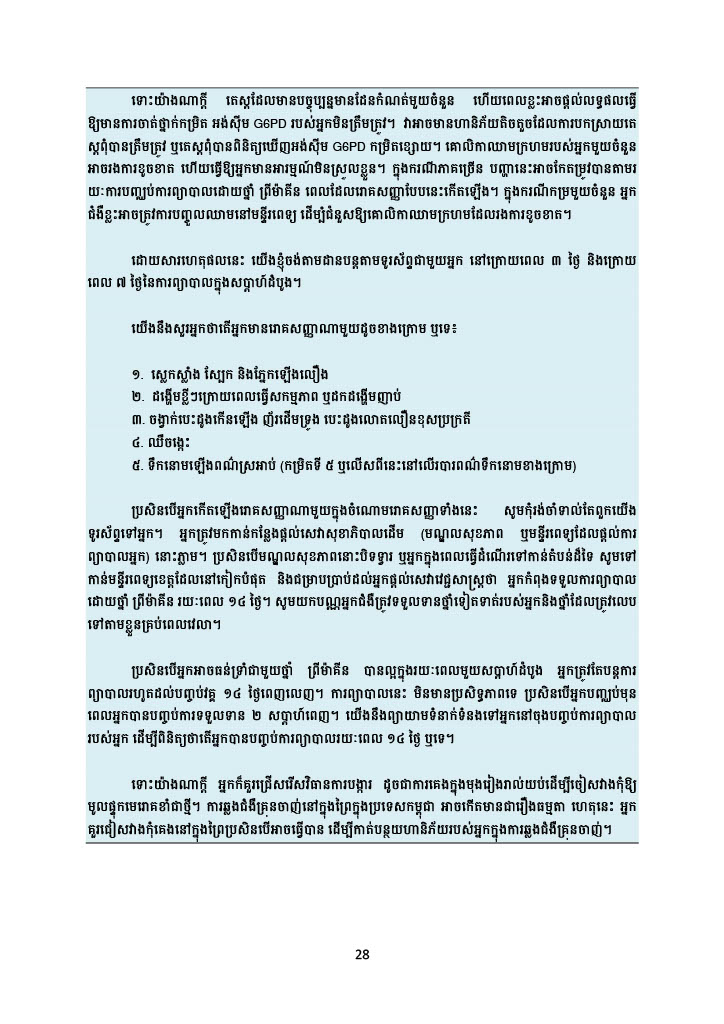

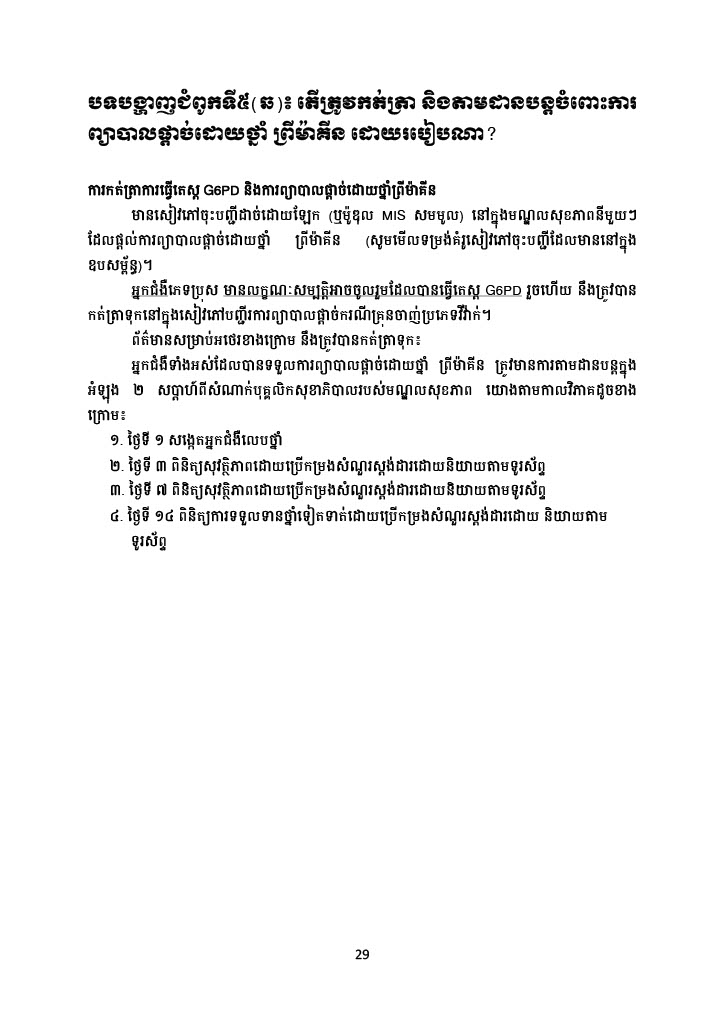

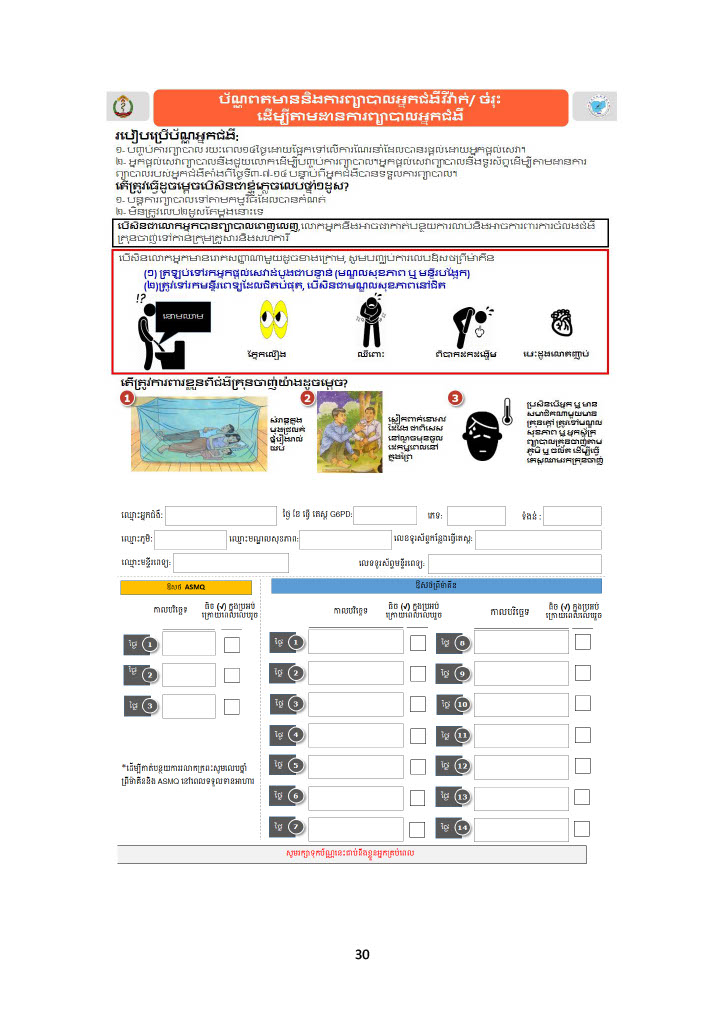

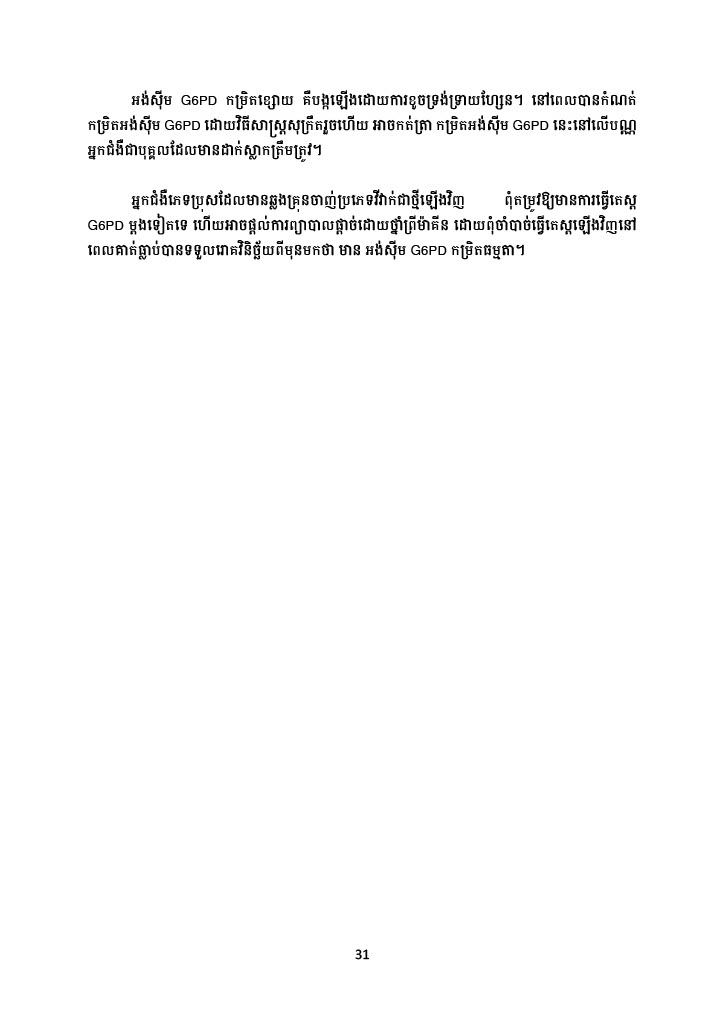

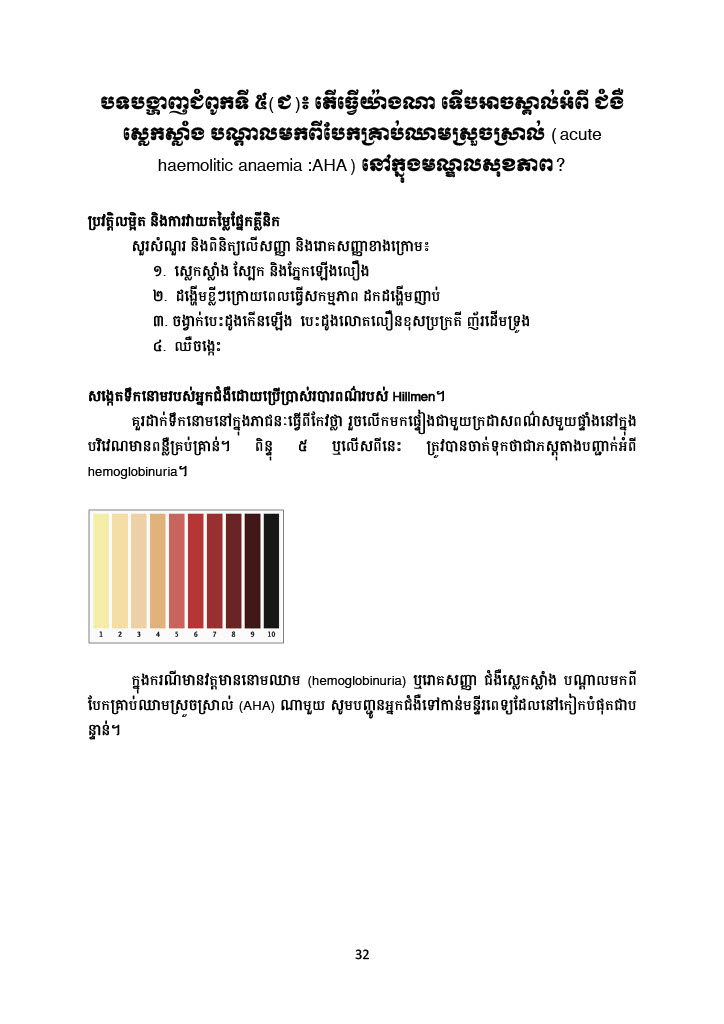

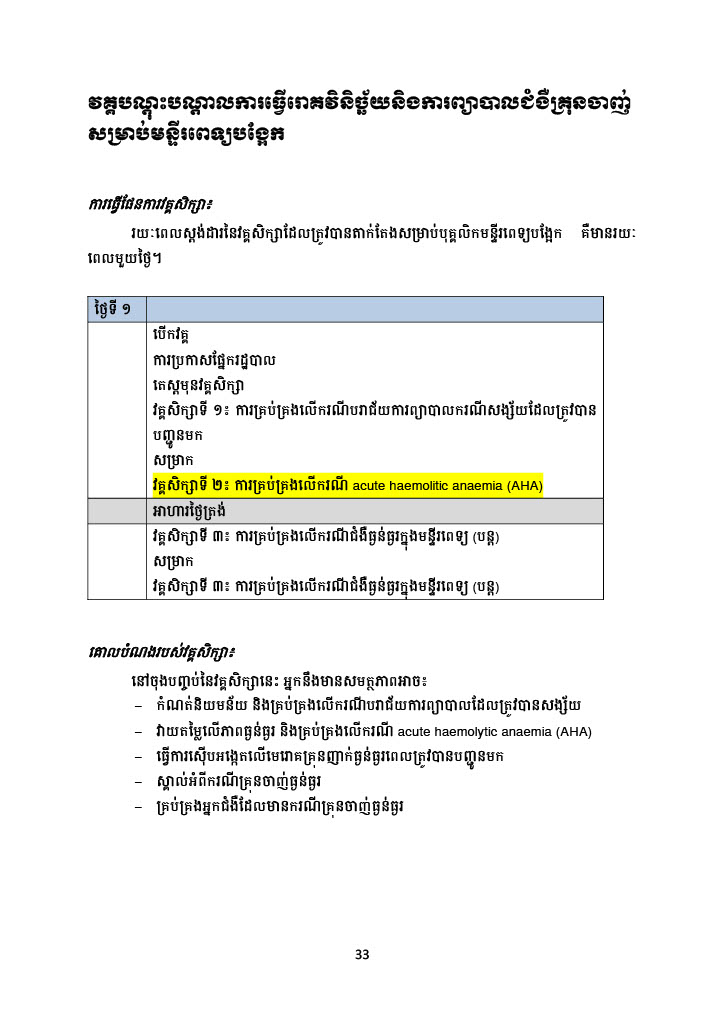

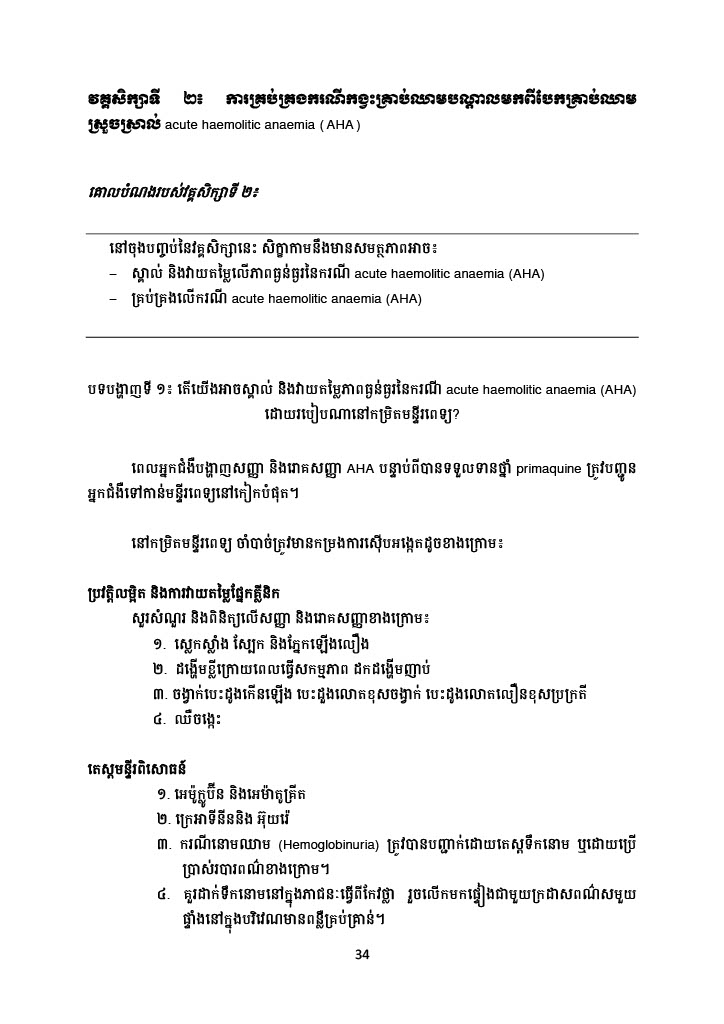

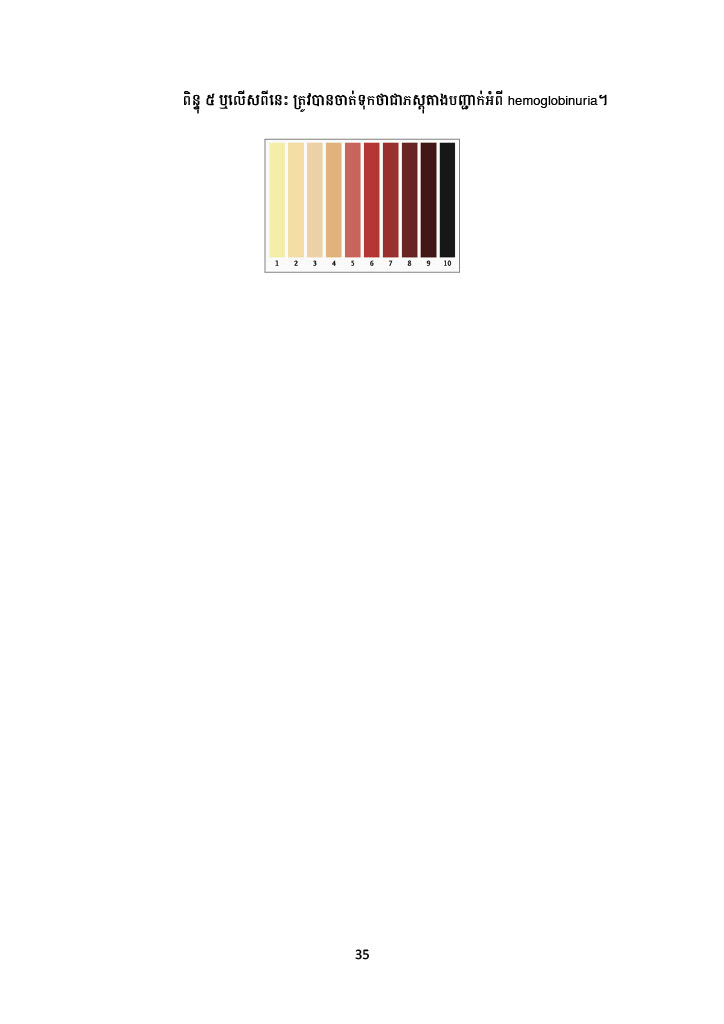

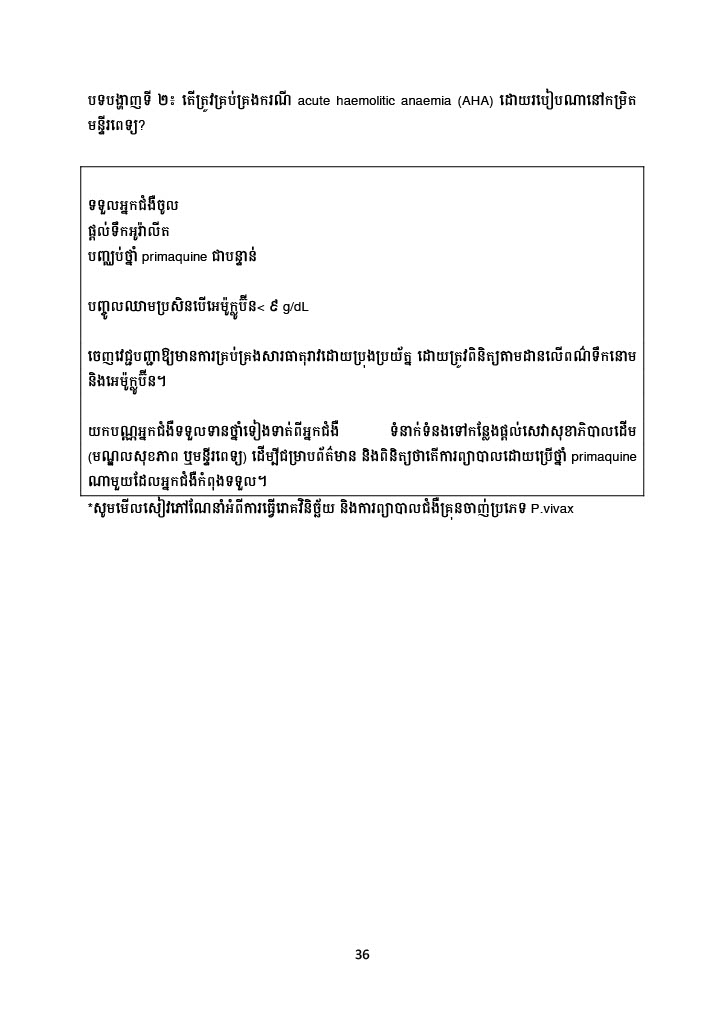

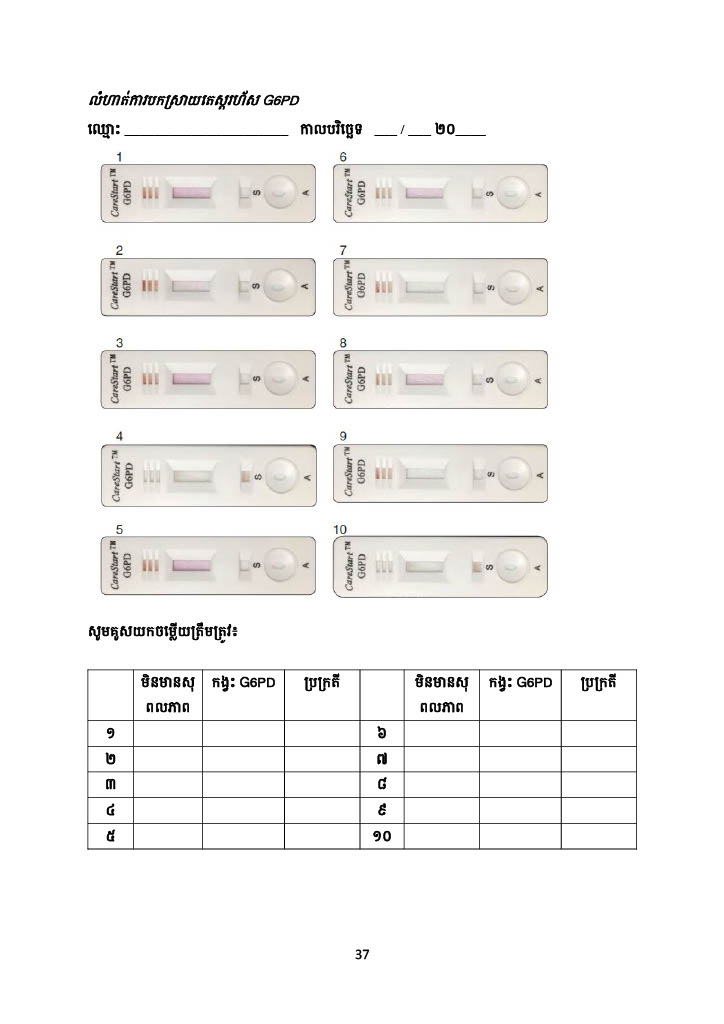

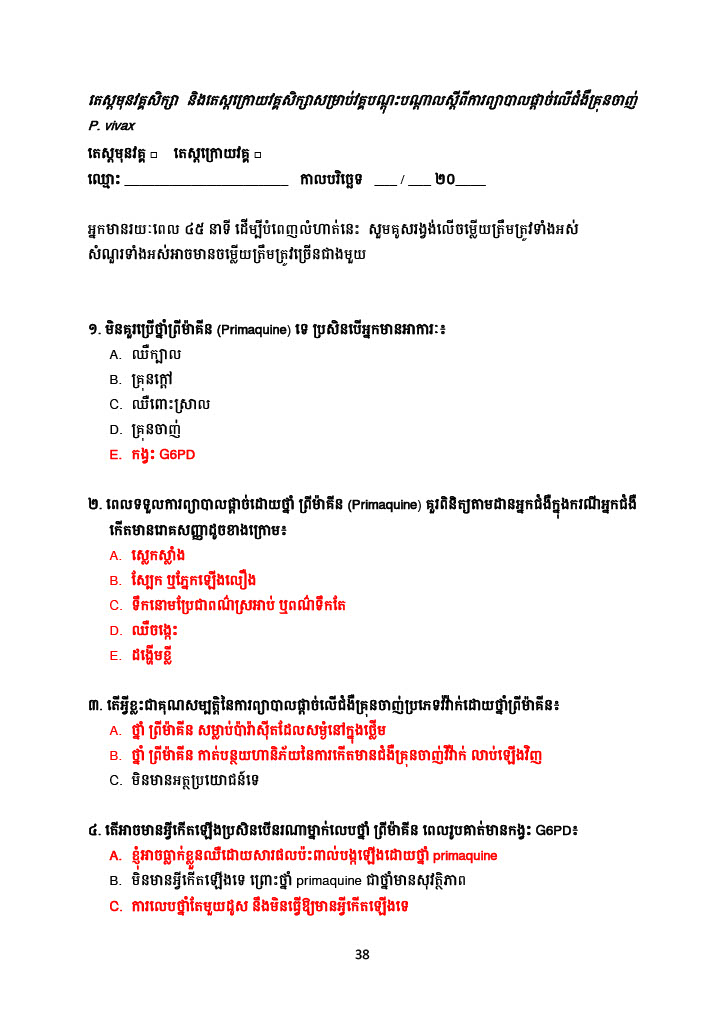

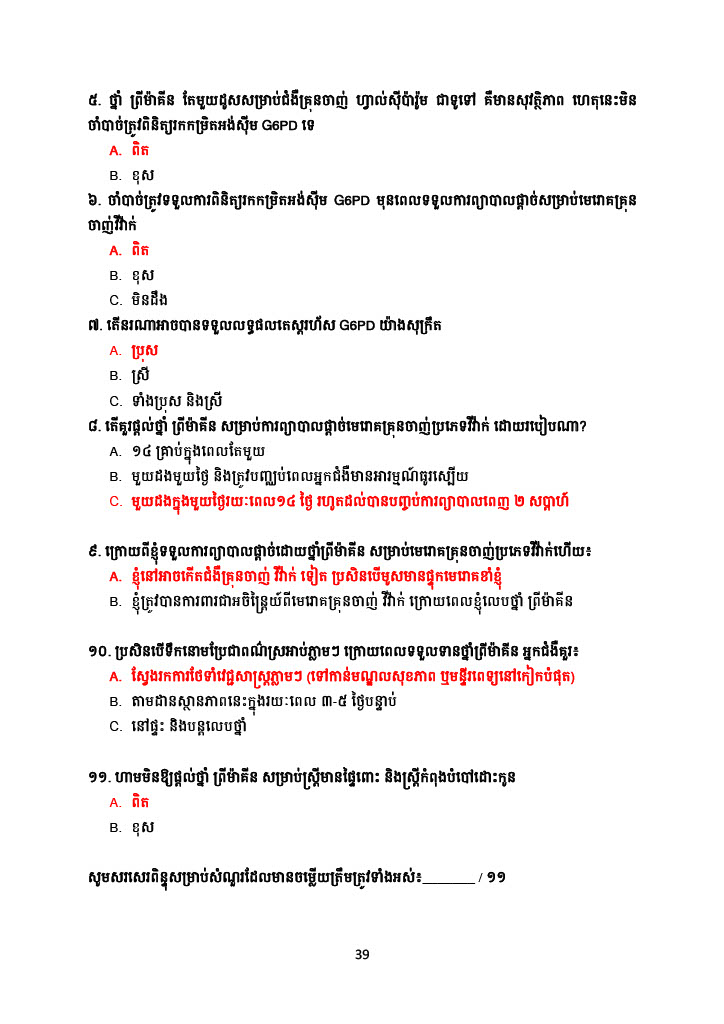

Supplement: S1 Appendix — (DOCX) [file pone.0275822.s011.docx]
