# Supplementary figures and images for: G6PD testing and radical cure for Plasmodium vivax in Cambodia: A mixed methods implementation study
Source: PLoS One. 2022 Oct 20;17(10):e0275822. doi: 10.1371/journal.pone.0275822 (PMC9584508; doi:10.1371/journal.pone.0275822)

**S2 Appendix:** G6PD patient card. Front and back pages are differentiated by the red dotted line.

--------------------------------------------------------------------------------------------------------------------------


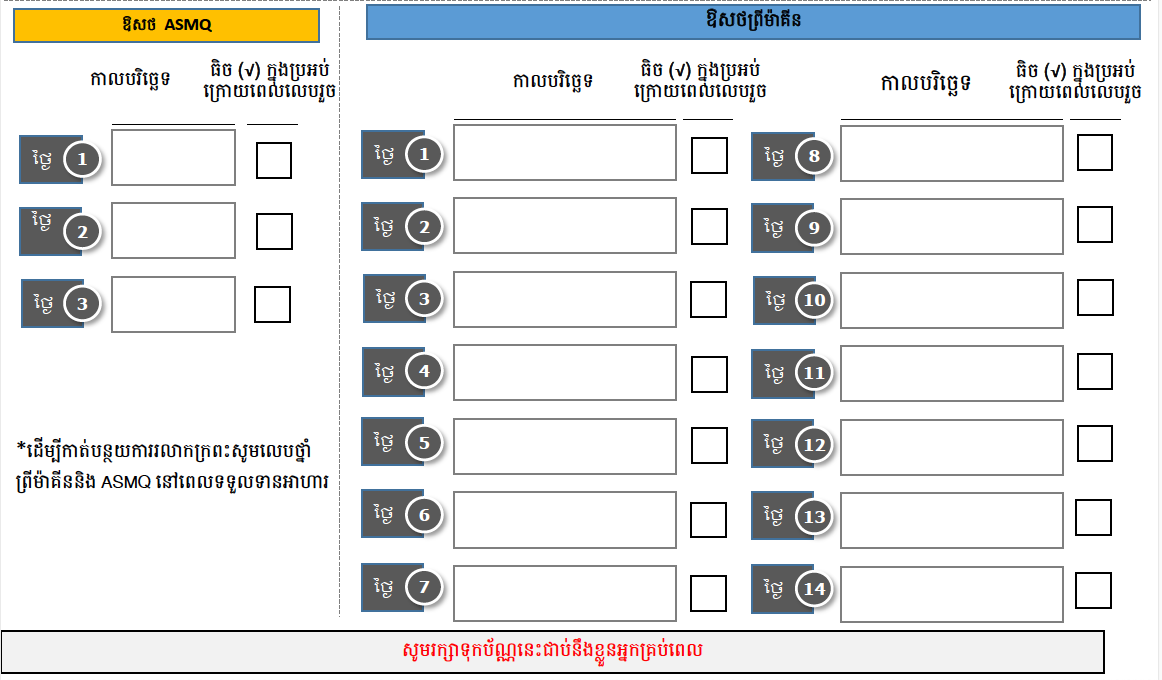

Supplement: S2 Appendix — (DOCX) [file pone.0275822.s012.docx]
