# Supplementary material for: G6PD testing and radical cure for Plasmodium vivax in Cambodia: A mixed methods implementation study
Source: PLoS One. 2022 Oct 20;17(10):e0275822. doi: 10.1371/journal.pone.0275822 (PMC9584508; doi:10.1371/journal.pone.0275822)
Supplement: S3 Appendix — (DOCX) [file pone.0275822.s013.docx]

**S3 Appendix:** Paper forms used by HC staff and VMWs to collect and store patient information.

Paper forms were used by healthcare workers to record patient information when they registered for G6PD testing (Fig A) and to document events at follow-up sessions, including adherence and reported side effects (Fig B).

**Fig A:** List of patients registered for G6PD testing, with patient information and baseline investigations on page 1 and treatment pathway outcomes on page 2. Pages are differentiated by the red dotted line.

---------------------------------------------------------------------------------------------------------------------------


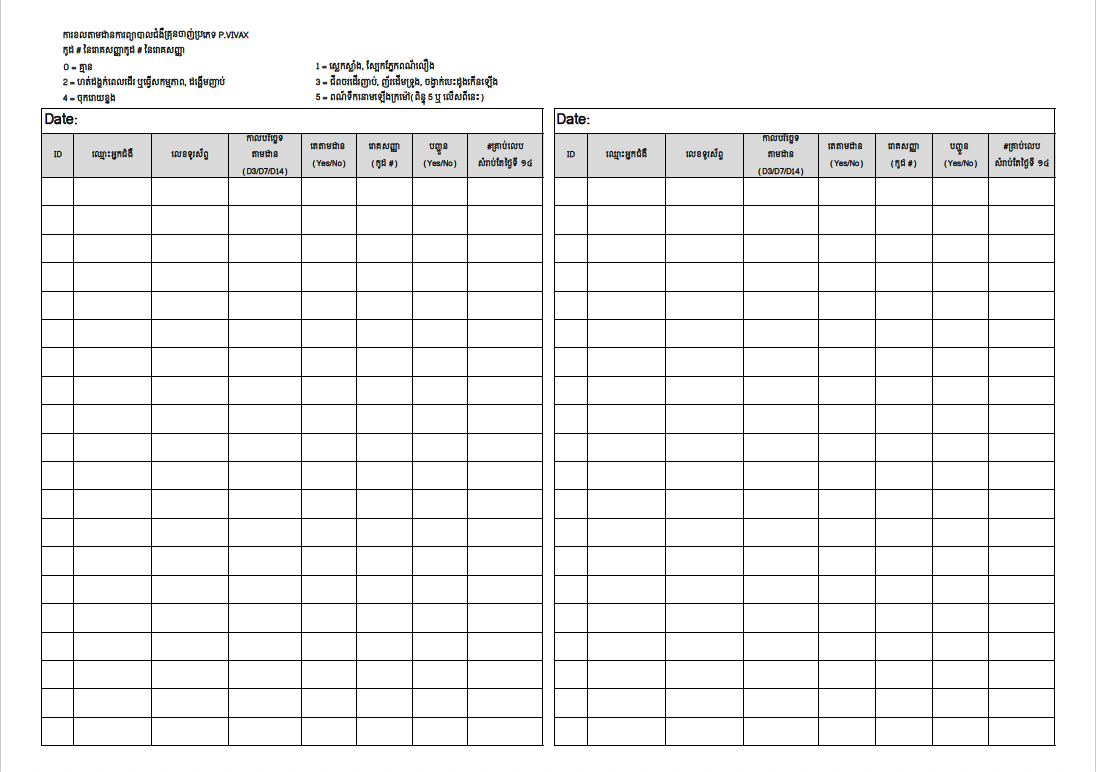


**Fig B:** Paper form used by VMWs at each follow-up session to record patient details, adherence and potential side effects. Side effects are listed in rows 1-15, and include: fever, chills/shivering, headache, nausea and/or vomiting, abdominal pain, dizziness, fatigue, shortness of breath, skin rash or itchiness, jaundice or pallor, urine discolouration, back pain, treatment interruption due to possible side effects, other.
