# Supplementary material for: G6PD testing and radical cure for Plasmodium vivax in Cambodia: A mixed methods implementation study
Source: PLoS One. 2022 Oct 20;17(10):e0275822. doi: 10.1371/journal.pone.0275822 (PMC9584508; doi:10.1371/journal.pone.0275822)
Supplement: S4 Appendix — (DOCX) [file pone.0275822.s014.docx]

**S4 Appendix:** Screenshots of the *P.v* module, integrated into the National Center for Malaria Control’s MIS (Malaria Information Systems) mobile app.

Figures A-C show the mobile app at different stages of use, displayed on a health center tablet.

**Fig A:** MIS app home screen with notification alert message appearing at the top of screen.


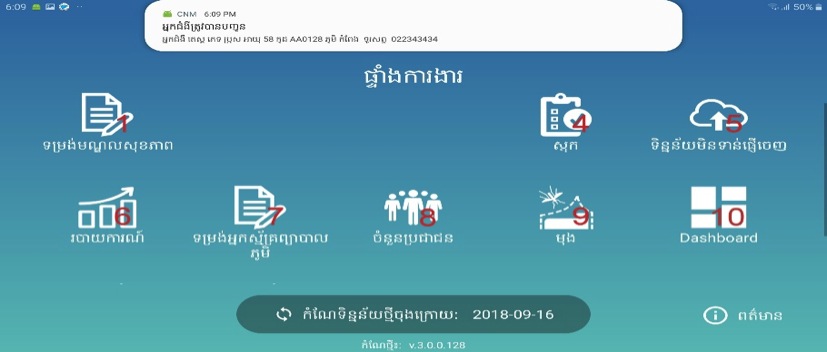


**Fig B:** List of patients in the *P.v* module, displaying unique patient ID and other patient details. The red circle indicates the button that staff can press to open the page shown in Fig C (below).


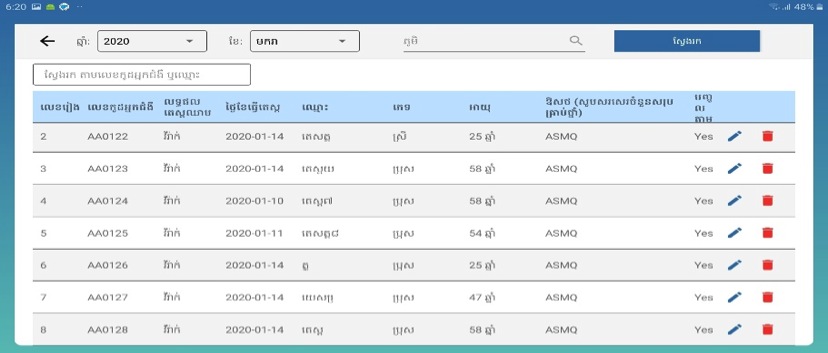


**Fig C:** Screenshot of page where patient details can be edited by healthcare staff, including areas where G6PD test result and type of treatment initiated can be entered and/or selected.


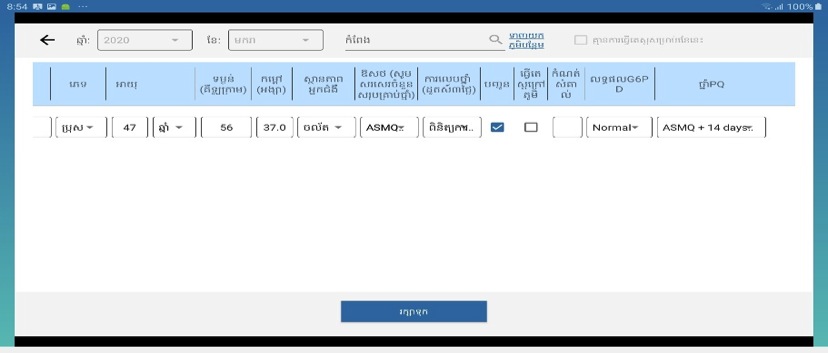


*P.v* = *Plasmodium vivax*. ID = identification code. G6PD = glucose-6-phosphate dehydrogenase.
