# Supplementary material for: G6PD testing and radical cure for Plasmodium vivax in Cambodia: A mixed methods implementation study
Source: PLoS One. 2022 Oct 20;17(10):e0275822. doi: 10.1371/journal.pone.0275822 (PMC9584508; doi:10.1371/journal.pone.0275822)
Supplement: S5 Appendix — (DOCX) [file pone.0275822.s015.docx]

**S5 Appendix:** Trends in malaria incidence in study areas.

Trends in malaria incidence were described from 2 years before study launch until end of study period (Fig A). Rapid decline in cases of all species was observed following VIGTARC interventions, taking seasonal trends into consideration. Population is expected to have remained fairly constant during this period. From July – December 2020, study areas recorded just 14.2% of the *P.v*/mixed malaria cases observed in July – December 2019 (1252 vs 178 cases; an 85.8% reduction). Seasonal peak for transmission in Cambodia occurs during the rainy season, from June to November annually [5]. A large seasonal peak corresponding to this period can be observed in 2018; this is much lower in 2019 and minimal in 2020.

**Fig A:** Number of malaria cases registered in the public sector of study areas, over time.


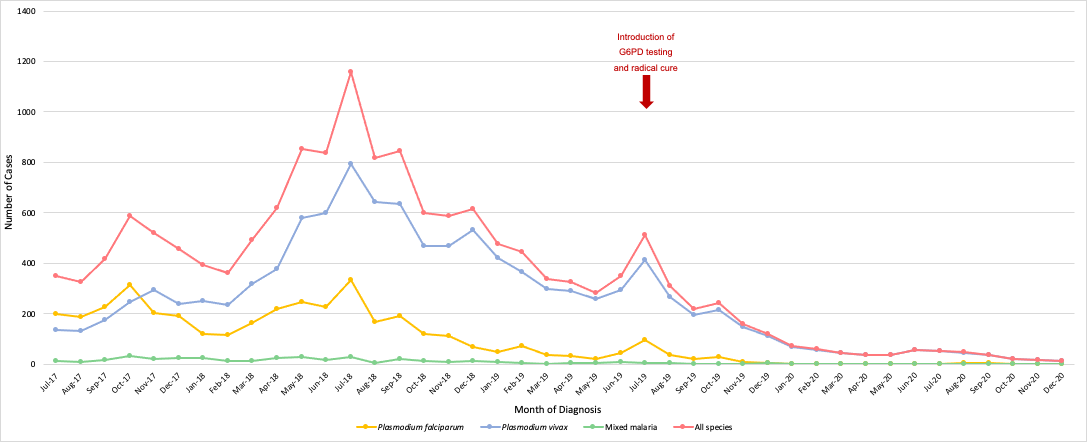


Red arrow indicates the time when the new care pathway was implemented, which introduced G6PD testing and radical cure for *P.v*/mixed malaria cases.
