# Supplementary material for: G6PD testing and radical cure for Plasmodium vivax in Cambodia: A mixed methods implementation study
Source: PLoS One. 2022 Oct 20;17(10):e0275822. doi: 10.1371/journal.pone.0275822 (PMC9584508; doi:10.1371/journal.pone.0275822)
Supplement: S6 Appendix — (DOCX) [file pone.0275822.s016.docx]

**S6 Appendix:** Findings from thematic content analysis of qualitative interviews.

Key recurring themes and sub-themes, identified through thematic analysis of qualitative data (in addition to those discussed in the main text), are described below. Themes and initial coding lists have been demonstrated in S3 Table.

### *G6PD testing*

All HC staff interviewed had performed G6PD testing, but experience varied considerably between HC roles. Overall, quantitative G6PD tests were preferred over qualitative. Qualitative tests took longer, could present ambiguous colour results that were difficult to interpret and were insufficient for females; these disadvantages outweighed the fact that qualitative tests were often reported as technically easier to perform.

Actually, the RDT test is not clear like biosensor test. Sometimes, when we tested using it, the result showed the colour unclearly. So, sometimes it is hard for us to diagnose whether the result is deficient or normal. But biosensor test is good because it shows G6PD clearly and haemoglobin clearly as well.

HC05, HC Staff

When asked if VMWs would be capable of safely performing G6PD testing and initiating radical cure, participants widely reported that this would not be possible due to high variation in VMW capacity/knowledge. Reliable cold storage of G6PD tests in the community was also considered unachievable.

In this part, I don’t want to say. Because we don’t have specialisation. The health facility staffs(s) have the specialisation for the treatment more than us… I don’t have any specialisations. We can’t do as the same as them.

V[Unknown], VMW

***Primaquine treatment***

### *Uptake*

Participants reported that most vivax-affected individuals were highly motivated to initiate treatment, especially due to negative socioeconomic impact of frequent relapses.

The reason that I received P.v radical cure medicine [was] because it [P.v] could make me [have] difficulty which could reoccur or relapse during my work when I hadn’t received radical cure yet.

P03, Ex-patient

All the patients interviewed had accepted treatment, however all participant groups reported knowledge of cases where primaquine had been declined. This was perceived to result from individuals prioritising short-term economic benefits of returning to work quickly or, to a lesser extent, difficulty travelling to HCs.

They didn’t [accept radical cure]… Because they needed to drink medicine daily for 14 days and during this treatment, they weren’t allowed to go forest. Therefore, it was relating to their economic [situation] and their livelihood.

HC08, HC Staff

There was a patient who said that he committed not to be treated with it [primaquine]. He was in debt… Therefore, he needed to go to forest every day [to work]… They don’t have times for this medicine drinking.

V[Unknown], VMW

Patient counselling/education was frequently described as an important factor in encouraging uptake.

There were some patients who hesitated to go there, too; but VMW explained them more such as “If you aren’t treated with its doses or its levels, you can’t be radical[ly] cured unless you are treated with radical cure”... VMW explained like that until the patients decided to go there.

CL07, Community Leaders

Patients were highly willing to encourage others to participate in the radical cure programme. This primarily resulted from a desire to see others cured like themselves. Furthermore, it benefited their own work if colleagues were not repeatedly relapsing. There were numerous examples of individuals who had been encouraged by peers to receive G6PD testing and radical cure, when they were hesitant before.

After that [the treated patient] told another patient to come here for radical cure like him/her as well. This is benefit. Before radical cure, he/she was relapsed frequently once a month and after radical cure with primaquine, he/she forced their relatives and co-worker who was sick for treatment as well. This is lot of cases.

HC11, HC Staff

Accessing HCs for investigation/treatment was difficult for a very small minority due to extreme poverty and/or challenging roads impacting travel. Many VMWs routinely accompanied patients to HCs, to ensure they followed through and to simplify referral/enrolment processes.

There isn’t any difficulty [travelling to the health centre]. There is no difficulty because the distance is just near here and nowadays, everyone has motorbike to drive to there.

CL08, Community Leader

They [patients] don’t have money for travelling [to the health centre] including motorbike, etc. And they don’t have enough money for food during traveling to there.

CL05, Community Leader

There was evidence that after patients were diagnosed with *P.v*, treated with ASMQ by the VMW and referred to HCs for G6PD testing, there was sometimes a delay in patients attending the HCs. This was especially common if patients were diagnosed by VMWs late in the evening, in which case they would often wait until the next day before attending HCs. During the rainy season, travel difficulties could result in longer delays, up to five days as reported by HCWs. This may have resulted in some mismatch between the monthly numbers of patients diagnosed with *P.v* and those tested for G6PD status, since patients may have been diagnosed and tested for G6PD status in different months.

However, I don’t give him the medicine [ASMQ] to drink if it is at noon time; vice versa, I give him the medicine [ASMQ] to drink if it is in the evening and I will go to health center with him on tomorrow morning.

V[Unknown], VMW

For example, a patient [remains] already 5 days in his/her area before s/he [is] refer[red] for G6PD testing here. It is because the distance is far away or floating. This is the obstacle. For example, at the moment a case is coming from Anlong Reap health center, the obstacle is road condition, so s/he wait and wait until 5 days.

HC12, HC Staff

### *Delivery in the new care pathway*

Overall, the care pathway was perceived as extremely beneficial and manageable for HCWs and patients alike. DOT follow-up was a major strength of the programme, highlighted by participants for improving adherence and safety and strengthening VMW-patient relationships. Patients were happy to be managed at home rather than in hospitals/HCs, as they could perform some work.

I like the part of having people to look after me at my home, to follow-up my disease and I had time for doing work at my home.

P[Unknown, Ex-patient

*Adherence*

Patients and staff reported good compliance with treatment in order to prevent relapses. Because recurrent relapses caused substantial opportunity costs across patients’ lifetimes, the long treatment duration was acceptable to patients interviewed, even if they couldn’t work during the course. However, some reported that remembering to adhere could be challenging for some patients, who needed to be reminded regularly.

The PQ14 DOT follow-up schedule (day 4, day 7, day 14) was described to be adequate and sufficient by 10/12 HC staff and 18/18 VMWs; 2 HC staff felt that more sessions would be beneficial. Notably, extra follow-up by VMWs in addition to the 3-day DOT schedule was frequently described, either via phone or in person with the aim of ensuring adherence and/or safety. Therefore, patients actually received varying amounts of follow-up. This could suggest that VMWs were not fully confident with 3-day DOT.

The DOT has the benefits because we know whether the patients drink medicine enough or not because sometimes, they missed and/or forgot. So, we can check their medicine. We can know the side effect of the patients… When we go to visit them, they are happy. They thought that after giving medicine, just leave from them without visiting them. Therefore, we go to ask them, they are happy.

V04, VMW

Travel to follow-up sessions was sometimes challenging for VMWs due to costs and poor road conditions, especially during rainy season. Some VMWs suggested reimbursement of transportation fees to VMWs who needed to travel considerable distances.

The care pathway may not be optimal for minority subgroups such as mobile and remote populations. VMWs expressed difficulty travelling to remote areas for DOT, and that mobile patients were less likely to stay in one place to adhere to treatment and follow-up. Suggestions for improvement in very remote locations included protocol change to allow follow-up via phone and establishing specific VMWs for such areas.

The weakness is [the] mobile patient. It is hard to convince them to use radical cure 14 days, 8 weeks. Because of the duration, they could not adhere. Therefore, this is the weakness of the project.

HC12, HC Staff

###

Aside from occasional phone follow-up and extra DOT follow-up sessions delivered by VMWs, high fidelity to the proposed care pathway was described by HCWs and patients. When describing the steps that were taken by HCWs in managing patients, there was mostly no deviation from the care pathway protocol and guidelines. There were some instances of stages being omitted by HCWs when describing the steps taken, however most were able to recall all the steps when prompted for further details in an area of the pathway.

### *Perceived efficacy*

All participants interviewed felt that primaquine was very effective in preventing *P.v* relapse; because of this, many enthusiastically expressed that they liked/supported this medication, and the whole radical cure programme. An extremely high level of confidence in primaquine efficacy was described and attributed to first- or second-hand experience with individuals who had been treated and remained relapse-free. Gaining perceived proof of efficacy through personal or shared experience was an important factor, as some reported that it had alleviated their pre-existing skepticism. Perceived efficacy was also described through its indirect impact on other areas of life, especially in reducing the negative effects on socioeconomic status and health.

I think primaquine has high effectiveness for P.v malaria. I’ve seen that it is good, good, good.

HC05, HC Staff

At the beginning, I didn’t believe this medicine. I heard from VMW who said that nowadays [we have] the medicine for P.v radical cure for 14 days treatment, but we hadn’t believed yet. Now we treat with it and [it] can heal [for] many months now, from 2019 until now. I never reoccurred or relapsed with it. Therefore, I just want to say “[I] Like this medicine which can make me heal from malaria and I have time to work to support my life”.

P01, Ex-patient

A significant reduction in malaria incidence (both *Plasmodium falciparum; P.f* and Plasmodium *vivax; P.v*) in study areas was widely reported by village malaria workers (VMWs), health centre (HC) staff and community leaders. Local incidence trends were not discussed in patient interviews. The majority felt that the reasons for the perceived decrease were multifactorial. Four key contributing factors were identified: restrictions on forest-going, availability of radical cure, establishment of VMWs and utilisation of bite-avoidance (prevention) methods.

V02: 1st [the reduction in malaria cases is] because of radical cure. 2nd they rarely go to forest presently because the government banned for entering.

V04: They have lot awareness. They sleep using mosquito net.

VMW discussion

Healthcare workers (HCWs; including both VMWs and HC staff) felt that a large proportion of their cases before the radical cure programme comprised a small number of individuals who relapsed repeatedly and often. They extrapolated that by treating a relatively small number of patients to prevent relapses, a dramatic reduction in incidence had resulted.

The cases usually were the relapsed cases. Each patient were the same cases. After receiving the radical cure treatment, s/he never relapse.

V[Unknown], VMW

In previous times, the rate of the malaria increased because of the same cases which relapsed one time a month which accounted for 12 times or 7 or 8 times per year; so, these cases were only one patient; therefore, the cases had increased. Nowadays, when we treat patient(s) with radical cure, we don’t see the same person. It means that we see only the new case(s) or new infection(s) because we don’t see the former P.v patients who already were treated… Therefore, we have seen that the cases have dropped down a lot; and the programme is good for them.

HC08, HC Staff

### *Safety*

Primaquine was widely described as safe by patients, VMWs and HC staff. Safety was not discussed with community leaders. Potential adverse events including haemolysis were acknowledged, but no participants had witnessed or experienced serious adverse events. Many HCWs expressed that correct usage of glucose-6-phosphate dehydrogenase (G6PD) testing and timely drug administration mitigated primaquine-associated risks. Patient counselling and directly observed therapy (DOT) follow-up were highlighted as important for improving safety, as they allowed early identification of potential adverse events. Patient groups could recall ‘red flag’ symptoms to monitor for and need to contact HCWs if they developed.

It has benefit and it is dangerous, too if we don’t perform the enough testing and correctly by giving patient to drink without specific confirmation which is a problem that is dramatically notice.

HC01, HC Staff

He [HCW] told me that “you must keep medicine well because if your younger brother/sister take it to drink, then s/he will be haemolysis.”

P[Unknown], Ex-patient

Mild adverse events were felt to only occur initially and self-resolve by days 3-5 of 14-day primaquine (PQ14) treatment. Many considered these early symptoms to be caused by artesunate-mefloquine (ASMQ), rather than primaquine, which they recognised to frequently cause mild but unpleasant symptoms from previous experience. Patients were also mostly asymptomatic whilst taking primaquine exclusively. Overall, no serious safety concerns were reported.

When I drank the medicine until day 3, I feel asleep but nothing problem. I just tiredness. After these days, I feel okay.

P03, Ex-patient

In my opinion for a P.v patient, I think that I have ever seen its side effects only day 1, day 2 and day 3 which relating with ASMG use such as fatigue, tiredness and can’t walk. But for the primaquine… I have never seen any side effects which is dramatically notice which the patients have the problem with primaquine use. The majority of the people who we asked said that “For day 4, s/he doesn’t have side effects”.

HC03, HC Staff

A frequently recurring suggestion from all participant groups was that the radical cure programme should be continued. Many were concerned about the programme ending too soon, before elimination is achieved.

If this pathogen/parasite or this disease isn’t eliminated yet, please continue to run this programme. Please, still support it progressively.

CL08, Community Leader

### *HCW training*

Overall, most HCWs felt that the training content was of good quality but that further training/refresher sessions would improve or maintain service delivery. HCWs reported receiving very variable amounts of training, ranging from 0-8 for HC staff. This was considered a barrier to optimal sharing of workload in HCs. 8/12 HC staff (66.7%) felt they would benefit from additional training. VMWs expressed that monthly meetings with refresher training improved their ability to carry out their roles. Most VMWs expressed the desire for further/continued training.

### *Data management*

HC staff felt that paperwork was excessive and time-consuming. However, they also expressed it was important and had no suggestions for reducing quantity. An alternative suggestion for improving workload was to provide/train more staff.

HC staff and VMWs reported that the mobile/smartphone app provided value by allowing fast sharing of information, reminding VMWs of when to perform DOT and by saving time for documentation. These benefits were felt to outweigh the technical challenges, such as poor signal in certain areas and slow tablet devices. Suggestions for improvement included replacing tablets with newer devices and providing an extra tablet for each HC (two total).

The tablet doesn’t work well. It is stuck. It’s not easy for using as phone. I feel tired of using it… It [tablet/smartphone app] could help us for on time reporting. After entry data, the data will transfer. We don’t need to fill the document (report) by hand writing… It has more benefit than difficulty.

HC04, HC Staff

A recurring view was that some VMWs, particularly those of older age, had difficulty with using smartphone apps due to inexperience with devices and poor eyesight. Further training was suggested.

The older aged VMWs are hard to see [the smartphone]. If in the future has this programme, we request a bigger to make them easy to see from their eyes. And the majority of the older aged VMWs when they see it, they don’t even understand… Therefore, I request to train the smartphone more, especially in the monthly meeting to remind them.

V09, VMW

### *Primaquine supply and procurement*

### All HC staff interviewed stated that they had no issues with primaquine supply during their experience of the radical cure programme; no stock-outs were reported. Many of the HC staff were unsure of where the primaquine came from, and how it was procured, but were happy with the supply.

Relating to the medicine, there is no problem because we never lack of medicine. The medicine is enough.

HC01, HC Staff

One HC staff described congestion in the drug depot due to an excess of primaquine medications. They felt this had resulted from an overestimation of primaquine quantity needed, based on historical case incidence. However, the number of cases had declined rapidly resulting in slow use of supplies and congestion.

HC05: I think that the drug depot in our health center will be congested because of many medicines… This congestion is because in the previous time there were lots of cases; however, the cases become less now… When the cases decrease, the medicine becomes congested.

Interviewer: When it remains the medicine for [a] long time, it makes the medicine expired.

HC05: Yes. This medicine will be expired after 2 years or 3 years. Therefore, when the cases remain less, it will [be] face[d] with congestion. So, we need to solve this problem.

HC Staff discussion

### *Tafenoquine*

It was widely felt that single-dose radical cure would be better than 14-day or 8-week courses, as it would prevent poor adherence and reduce the need for resource-intensive DOT follow-up. A minority of HCWs expressed some concern about serious adverse events, which they believed would be more difficult to manage following single-dose treatment compared to longer courses which they trusted through experience.

HC01: The important [thing] is that one time [radical cure] is “all the 14 [doses] combined into one”. If it already goes into [the patient’s body], how about the patient who has the problem with the medicine? What should we do? If we put it in, it will [go] into them, we can’t take it out.

Interviewer: If there is this medicine, you would like to use this medicine?

HC01: If there is the programme to heal for only one-dose treatment for radical cure, I am happy to receive, too, [if] the ministry of health or government authorized that medicine nationally. If it is authorized, we don’t reject. It is also good; it can reduce so much if it really has that medicine.

HC Staff discussion

However, all agreed that if it was deemed safe and authorised by senior healthcare advisors then they would be keen to implement it. HCWs felt patients would be less likely to decline treatment, and patients expressed they would be even happier to take single-dose treatment as it would save them time and allow a quicker return to work.

P02: It doesn’t waste our time as the same as the 14 days medicine.

P[Unknown]: I want it because it can save more time than the 14 days medicine.

Ex-patients’ discussion
