# Supplementary material for: G6PD testing and radical cure for Plasmodium vivax in Cambodia: A mixed methods implementation study
Source: PLoS One. 2022 Oct 20;17(10):e0275822. doi: 10.1371/journal.pone.0275822 (PMC9584508; doi:10.1371/journal.pone.0275822)
Supplement: S7 Appendix — (DOCX) [file pone.0275822.s017.docx]

**S7 Appendix:** Analysis of haemoglobin trends in the 8-week primaquine (PQ8W) course.

Haemoglobin was measured on days 1, 4, 5 and 8 of the PQ8W course. At each testing point, a varied number of individuals received testing (range 52-61). Average haemoglobin decreased initially but showed a small improvement on day 8. Haemoglobin trends are shown in Fig A in and Table A.

**Fig A:** Haemoglobin trends in the 8-week primaquine (PQ8W) course over time.
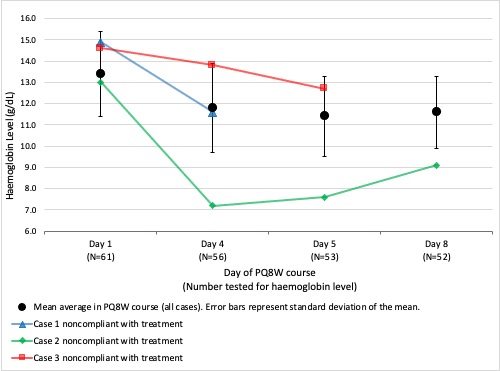


**Table A:** Haemoglobin trends for during the 8-week primaquine course.

| Day | Number of Participants Tested | Mean Hb [SD], g/dL | Change in mean Hb from previous, g/dL | Change in mean Hb from day 1, g/dL | Mean fractional Hb change from day 1 (%) |
| --- | --- | --- | --- | --- | --- |
| Day 1 | 61 | 13.4 [2.0] | -- | -- | -- |
| Day 4 | 56 | 11.8 [2.1] | - 1.6 | - 1.6 | - 11.9 |
| Day 5 | 53 | 11.4 [1.9] | - 0.4 | - 2.0 | - 14.9 |
| Day 8 (week 2) | 52 | 11.6 [1.7] | + 0.2 | - 1.8 | - 13.4 |
| Hb = haemoglobin, measured on day 1 (using HemoCue® or STANDARD^TM^ Biosensor test) and days 4, 5 and 8 (using HemoCue® test). | | | | | |

52 participants received haemoglobin testing on both day 1 and day 8. Of those, the majority experienced a decrease in haemoglobin between day 1 and 8, however 8/52 (15.4%) experienced an increase. There was good evidence for a strong negative correlation between baseline haemoglobin and fractional change in haemoglobin from baseline at day 8 (r = -0.7184, p<0.0001), after removal of three outliers; those with higher baseline haemoglobin experienced a larger decrease in haemoglobin than those with lower baseline haemoglobin. Relationship between baseline haemoglobin and fractional chance from day 1 to day 9 is shown in Fig B.

**Fig B:** Baseline haemoglobin and fractional change in haemoglobin from day 1 to day 8 of the 8-week primaquine course.

• Data points (excluding outliers).

• Indicates outlier.

••••••• Line of best fit, assuming linear relationship and excluding outliers.

Findings are consistent with those of Commons et al. who observed initial haemoglobin falls in non-anaemic patients taking primaquine, which was absent in anaemic patients who instead experienced steady increase (Fig C) [44]. Crucially, both patient groups had increased haemoglobin levels at day 42, indicating long-term benefits in all [44].

**Fig C:** Mean haemoglobin-time profiles in patients with *Plasmodium vivax* treated with chloroquine alone, or chloroquine and primaquine, adapted from Commons et al. [44].

B) Individuals with baseline haemoglobin ≥11.5 g/dL. C) Individuals with baseline haemoglobin <11.5g/dL. Profiles for CQ alone and CQ+PQ adjusted to the same baseline haemoglobin. Shaded regions show 95% confidence intervals. 0.1% and 1.2% of individuals had G6PD deficiency in the CQ+PQ and CQ groups respectively.


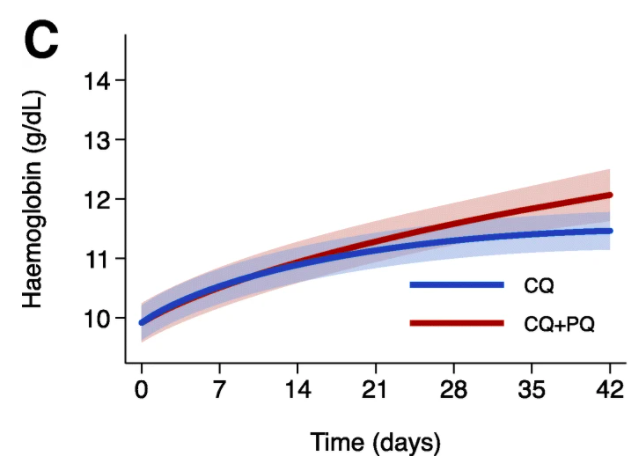

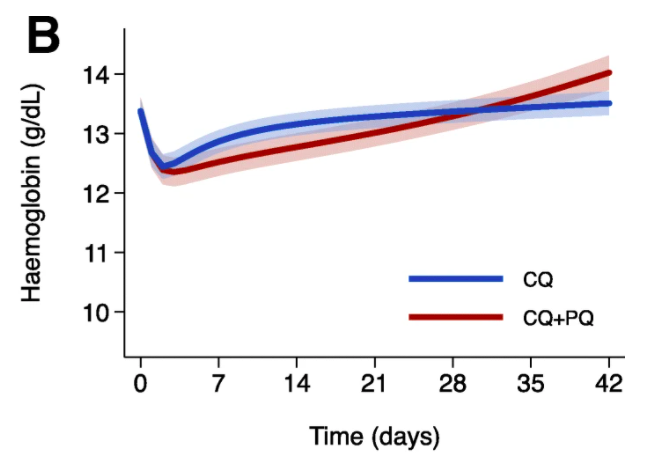


CQ = chloroquine. PQ = primaquine. G6PD = glucose-6-phosphate dehydrogenase.
